# New Dimension in Magnetism and Superconductivity: 3D and Curvilinear Nanoarchitectures

**DOI:** 10.1002/adma.202101758

**Published:** 2021-10-27

**Authors:** Denys Makarov, Oleksii M. Volkov, Attila Kákay, Oleksandr V. Pylypovskyi, Barbora Budinská, Oleksandr V. Dobrovolskiy

**Affiliations:** ^1^ Helmholtz‐Zentrum Dresden ‐ Rossendorf e.V. Institute of Ion Beam Physics and Materials Research 01328 Dresden Germany; ^2^ Kyiv Academic University Kyiv 03142 Ukraine; ^3^ Superconductivity and Spintronics Laboratory Nanomagnetism and Magnonics Faculty of Physics University of Vienna Vienna 1090 Austria

**Keywords:** curvilinear magnetism, curvilinear superconductivity, nanomagnetism, shapeable magnetoelectronics, superconductivity

## Abstract

Traditionally, the primary field, where curvature has been at the heart of research, is the theory of general relativity. In recent studies, however, the impact of curvilinear geometry enters various disciplines, ranging from solid‐state physics over soft‐matter physics, chemistry, and biology to mathematics, giving rise to a plethora of emerging domains such as curvilinear nematics, curvilinear studies of cell biology, curvilinear semiconductors, superfluidity, optics, 2D van der Waals materials, plasmonics, magnetism, and superconductivity. Here, the state of the art is summarized and prospects for future research in curvilinear solid‐state systems exhibiting such fundamental cooperative phenomena as ferromagnetism, antiferromagnetism, and superconductivity are outlined. Highlighting the recent developments and current challenges in theory, fabrication, and characterization of curvilinear micro‐ and nanostructures, special attention is paid to perspective research directions entailing new physics and to their strong application potential. Overall, the perspective is aimed at crossing the boundaries between the magnetism and superconductivity communities and drawing attention to the conceptual aspects of how extension of structures into the third dimension and curvilinear geometry can modify existing and aid launching novel functionalities. In addition, the perspective should stimulate the development and dissemination of research and development oriented techniques to facilitate rapid transitions from laboratory demonstrations to industry‐ready prototypes and eventual products.

## Graphical abstract

The rapidly developing field of curvilinear magnetism and superconductivity is reviewed. The covered topics range from novel physical concepts for material science through first proof‐of‐concept device demonstrations to established technologies of flexible magnetoelectronics. The review is relevant for a broad community of physicists, material scientists, and engineers, targeting fundamental research and technology transfer activities.

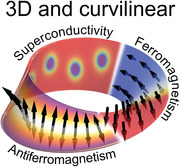

## 1 Introduction

The interplay between geometry and topology of the order parameter is one of the fundamental properties in soft and condensed matter physics, including cell membranes,^[^ ^1^ ^]^ nematic crystals,^[^ ^2^, ^3^ ^]^ superfluids,^[^ ^4^ ^]^ semiconductors,^[^ ^5^, ^6^, ^7^, ^8^ ^]^ ferromagnets,^[^ ^9^ ^]^ and superconductors.^[^ ^10^, ^11^ ^]^ Currently, much attention is paid to strongly correlated electronic systems, for example, ferromagnets and superconductors, as they provide a unique tool to manipulate the topology of coexisting vector and scalar fields, associated with geometries of conventional systems.

Until recently, in the case of magnetism, the influence of the geometry on the spin vector fields was addressed primarily by the design of the sample boundaries. This approach naturally brings the shape anisotropy to the system and leads to the formation of specific spin textures, for example, magnetic vortices^[^ ^12^ ^]^ and antivortices^[^ ^13^ ^]^ as well as provides control over the dynamics of the topologically nontrivial magnetic solitons.^[^ ^14^, ^15^, ^16^, ^17^, ^18^ ^]^ This discussion also tackles the state of the art in modern experimental antiferromagnetism, where the design of the sample topography and boundaries allows to control the domain wall states.^[^ ^19^, ^20^, ^21^, ^22^ ^]^ With the development of novel fabrication techniques allowing to realize complex 3D architectures, not only the boundary effects but also the extrinsic geometrical properties (e.g., local curvatures) can be addressed rigorously for the case of ferromagnets^[^ ^23^, ^24^, ^25^, ^26^, ^27^ ^]^ as well as superconductors.^[^ ^28^, ^29^, ^30^ ^]^ The explored effects are directly related to the interplay between the topology and chirality of manifolds and the order parameters. The topology characterizes the connectivity and boundaries of the concrete geometry. For example, in contrast to a ring, the Möbius ribbon has only one boundary curve due to a characteristic twist. It is also a chiral object which cannot be reconciled with its mirror image. The chirality of the Möbius ribbon determines the sense of the ribbon rotation as clockwise or counter‐clockwise with respect to the symmetry axis. These geometrical properties are reflected in the behavior of magnetic textures present in Möbius ribbons, making the magnetic domain walls to be topologically protected and determining their sense of rotation.^[^ ^31^ ^]^ The topology of the order parameter determines its irreducibility from the ground state by the presence of specific features which are characterized by the topological charge (Pontryagin index) *Q*.^[^ ^32^ ^]^ The difference of the geometrical topology of a curvilinear object, like between a spherical shell and a plane, results in the change of the topological charge of the magnetic texture living on the shell. For instance, magnetic skyrmions on a sphere are topologically trivial *Q* = 0 due to the shift of the magnetic topological charge caused by the genus of the spherical shell.^[^ ^33^ ^]^ The geometrical chirality can be linked with the chirality of the magnetic texture as it happens with the properties of domain walls^[^ ^34^ ^]^ and magnetoelectric responses^[^ ^35^ ^]^ of ferromagnetic helices.

The curvilinear geometry of the magnetic samples leads to the change of the magnetic responses, which acquire symmetries specific to the geometry.^[^ ^36^ ^]^ The geometrically broken symmetry provides a new toolbox to tailor magnetic responses of the material by providing additional, curvature‐induced anisotropy, and chiral responses.^[^ ^36^ ^]^ These effects are generic and independent of the choice of the magnetic material: any intrinsically achiral isotropic curvilinear ferromagnet could have responses typical for chiral and anisotropic materials.^[^ ^25^ ^]^ This could lead to the stabilization of chiral nontrivial magnetic textures, for example, magnetic domain walls (DWs),^[^ ^25^, ^37^ ^]^ skyrmions,^[^ ^38^ ^]^ or skyrmioniums^[^ ^39^ ^]^ localized at curvilinear defects. The possibility to tune magnetic responses by designing the geometry of a wire or magnetic thin film, is one of the main advantages of the curvilinear magnetism, which has a major impact on physics, material science and technology. At present, under its umbrella, the fundamental field of curvilinear magnetism includes curvilinear ferro‐ and antiferromagnetism, curvilinear magnonics and curvilinear spintronics. Shapeable magnetoelectronics is the application counterpart of the fundamental field of curvilinear spintronics,^[^ ^40^, ^41^ ^]^ which is focused on the development of wearable electronics for the smart skin applications, artificial magnetoception as six‐sence technology, motion control and touchless human–machine interaction.^[^ ^42^, ^43^, ^44^ ^]^ The nonreciprocity and asymmetric 3D geometries offered by curvilinear magnetic architectures are used in numerous biomedical applications for targeted drug delivery,^[^ ^45^, ^46^, ^47^, ^48^ ^]^ lab‐on‐chip applications^[^ ^49^, ^50^, ^51^, ^52^ ^]^ and artificial fertilization.^[^ ^48^, ^53^, ^54^, ^55^, ^56^, ^57^, ^58^ ^]^ Furthermore, the curvilinear magnetic wires with the controlled transport of DWs offer a dynamic reconfigurable architecture for prospective quantum information processing applications,^[^ ^59^, ^60^ ^]^ which are typically addressed using superconducting architectures.

The key difference in the impact of the curvilinear geometry on superconductors in comparison with (anti‐)ferromagnets lies in the underlying nature of the order parameter. In contrast to magnetic materials, for which energy functionals contain spatial derivatives of vector fields, the description of superconductors relies on the analysis of energy functionals containing spatial derivatives of scalar fields. While in magnetism the order parameter is the magnetization (vector), for a superconducting state the absolute value of the order parameter has a physical meaning of the superconducting energy gap (scalar). By extending hybrid (anti‐)ferromagnet/superconductor structures into the third dimension, it should be possible to explore the interplay between curvature effects in the system possessing vector and scalar order parameters. This progress strongly relies on the development of theoretical methods and the improvement of computation capabilities. In particular, a self‐consistent micromagnetic framework of curvilinear magnetism has recently been put forward.^[^ ^61^ ^]^ For superconductors, effects of curvature and torsion on the electronic system are not expected to be relevant as long as their characteristic scale is larger than the superconducting coherence length.^[^ ^62^ ^]^ At the same time, even in this case the state of a superconductor can be notably affected by geometry‐induced topologically nontrivial screening currents.^[^ ^63^ ^]^

It should be mentioned that confinement and manipulation of superconducting vortices have become a matter of extensive investigations in fluxonics^[^ ^64^, ^65^, ^66^ ^]^—the research domain emerged at the interface between superconductivity and nanotechnology. The geometrically broken symmetry leads to the appearance of effective fields, that allow the coexistence and interplay of topological defects of different types (vortices and slips of phase of the superconducting order parameter).^[^ ^67^ ^]^ For instance, superconducting planar nanostructures are widely exploited in diverse applications such as single‐photon detectors,^[^ ^68^, ^69^ ^]^ THz and GHz radiation emitters,^[^ ^70^, ^71^ ^]^ as well as circuits for quantum electrodynamics^[^ ^72^, ^73^ ^]^ and quantum computing.^[^ ^74^, ^75^ ^]^ Accordingly, extending superconducting low‐dimensional systems into the third dimension and curvilinear geometries provides access to physical phenomena, which do not occur in planar geometries and advances novel functionalities on the basis of curvature‐ and topology‐induced effects.^[^ ^76^ ^]^

The structure of the review is as follows. In Section 2, we introduce the basic terms of analytical geometry, which are relevant for the following discussion of the effects in curvilinear magnets and superconductors. In Section 3, we introduce the state of the art and perspectives in the field of curvilinear ferromagnetism. Much attention is dedicated to the emerging field of curvilinear antiferromagnets, which is described in Section 4. In Section 5, we present the state of the art and perspectives of curvilinear superconductors. Each of these sections summarizes the current state and future tasks in four directions of the prospective research: theoretical investigations, computer simulations, methods of sample fabrication and characterization. The perspectives of the application‐oriented research are given as well.

## 2 Vector Order Parameter in Curvilinear Manifolds

Before the discussion of physical effects, which are specific to curvilinear magnets and superconductors, it is instructive to introduce a conventional approach to describe curved geometries of nanowires and shells. They can be associated with different low‐dimensional manifolds such as curves (described by one coordinate) and surfaces (described by two coordinates).

**Figure 1:**
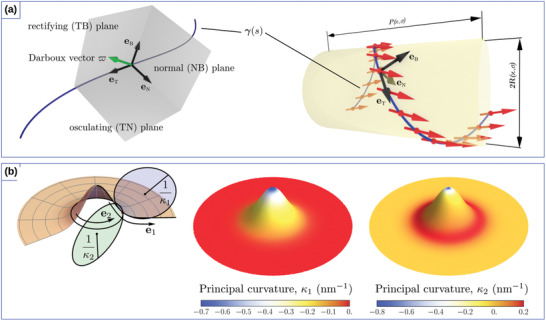
Curvilinear geometries. a) Moving trihedron (left): tangential (T), normal (N), and binormal (B) vectors defined at a space curve γ, and schematics of a helix‐shaped ferromagnetic wire (right). Unit vectors of the reference frame are shown in black and the magnetization direction is shown by red arrows. The helix has the pitch *P* and radius *R*. b) Gaussian bump with osculating circles that define principal curvatures, κ_1_ and κ_2_ at a specific point (left). The directions in the normal plane where the curvature takes its maximum and minimum values define the principal directions, $e_{1}$ and $e_{2}$. Spatial distribution of the principal curvatures: κ_1_ and κ_2_ (right).

The geometry of a nanowire can be described as the space domain $r=\gamma +x_{1}e_{N}+x_{2}e_{B}$ built around a flat or a space curve γ(s), where the perpendicular cross‐section is parameterized by the coordinates *x*_1,2_. Here, *s* is the arc length and the unit vectors of the so‐called moving trihedron (TNB reference frame) are used. If γ(s) is twice differentiable, they can be defined as^[^ ^77^ ^]^

(1)

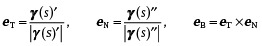

where $e_{T,N,B}$ represent the tangential (T), normal (N), and binormal (B) directions, see **Figure** **1**a. Here, prime means the derivative with respect to *s*. Their differential properties are described by the Frenet–Serret formulas:^[^ ^77^ ^]^

(2)

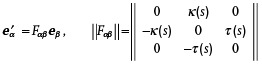

whereas Greek indices α, β = T, N, B enumerate curvilinear coordinates and components of a vector field, and the Einstein notation is used. The quantities κ(*s*) and τ(*s*) are curvature and torsion of the curve, respectively. For space curves, the definition κ > 0 with the arbitrary sign of τ is usually used, while flat curves are described by the signed curvature and zero torsion. The relation (2) can be shortened using the Darboux vector $\varpi =\tau e_{T}+\kappa e_{B}$. Then, 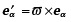.

Similarly, shells located in 3D space can be built as an extrusion of the surface $\varsigma (\xi_{1},\xi_{2})=r_{x}(\xi_{1},\xi_{2})\hat{x}+r_{y}(\xi_{1},\xi_{2})\hat{y}+r_{z}(\xi_{1},\xi_{2})\hat{z}$ by a constant value *h* along its normal. Here, ξ_1_ and ξ_2_ are curvilinear coordinates, which parameterize this surface. If ς is smooth enough, it is possible to introduce two tangential and one normal vector:

(3) $$g_{\alpha }=\partial_{\alpha }r(\xi_{1},\xi_{2}),\;\;\partial_{\alpha }=\frac{\partial }{\partial \xi_{\alpha }},\;\;\hat{n}=\frac{g_{1}\times g_{2}}{\left|g_{1}\times g_{2}\right|},\;\alpha =1,2$$

The metric properties of ς (and respective shell) are determined by the metric tensor (the first fundamental form), which is the second rank symmetric tensor^[^ ^77^, ^78^ ^]^

(4) $$\begin{array}{c} \left\|g_{\alpha \beta }\right\| \end{array}=\left\|\begin{array}{cc} g_{11} & g_{12}\\ g_{21} & g_{22} \end{array}\right\|,\;\;g_{\alpha \beta }=g_{\alpha }\;\cdot \;g_{\beta }$$

Together with the first fundamental form, the second fundamental form (shape tensor) should be introduced, which determines the way how the curved surface is embedded into the surrounding space^[^ ^77^, ^78^ ^]^

(5) $$\left\|b_{\alpha \beta }\right\|=\left\|\begin{array}{cc} b_{11} & b_{12}\\ b_{21} & b_{22} \end{array}\right\|,\;\;b_{\alpha \beta }=\hat{n}\;\cdot \;\partial_{\beta }g_{\alpha }$$

Both the first and second fundamental forms allow to determine the local geometrical invariants of the surface through the definition of the shape operator^[^ ^78^ ^]^

(6) $$\begin{array}{l} \left\|S_{\alpha \beta }\right\|=\;\frac{1}{g}\;\left\|\begin{array}{cc} b_{11}g_{22}-b_{12}g_{12} & b_{12}g_{22}-b_{22}g_{12}\\ b_{12}g_{11}-b_{11}g_{12} & b_{22}g_{11}-b_{12}g_{12} \end{array}\right\| \end{array}$$

where $g=\det(g_{\alpha \beta })$. As the shape operator can be diagonalized, its eigenvalues determine the geometrical invariants of the surface called the principal curvatures $\kappa_{1}(r)$, $\kappa_{2}(r)$, which represent the maximal and minimal values of the curvature at a given point of the surface, see Figure 1b. The product of the principal curvatures determines the Gaussian curvature $\tilde{K}=\kappa_{1}\kappa_{2}$, while their mean value $\tilde{H}=(\kappa_{1}+\kappa_{2})/2$ is called the mean curvature. The eigenvectors of the shape operator (6) uniquely determine the orthogonal basis vectors on the curved surface also known as principal directions $e_{1}(r)$ and $e_{2}(r)$. Together with the surface normal, they define the local curvilinear frame of reference also known as the Darboux reference frame. If the metric tensor *g*_*αβ*_ is in the diagonal form, then $e_{\alpha }=g_{\alpha }/\left|g_{\alpha }\right|$.^[^ ^78^ ^]^

To consider the given geometry as a thin nanowire or shell, the respective cross‐sections should be of the order or smaller than the characteristic length scales introduced by the magnetic sublattice in magnets or spatial extend of screening current loops in superconductors. This allows to eliminate the spatial variation of the order parameter within the cross‐section and describe nanowires and shells as effectively quasi‐1D and quasi‐2D objects, respectively.

The interplay between the curved geometry of the ordered matter and the vector field of the order parameter ultimately interconnects their topologies, which results in the appearance of the effective interactions and various geometrically induced topological defects in the order parameter field.^[^ ^79^, ^80^, ^81^ ^]^ In a mathematical perspective, topology is the property of geometrical objects or vector fields, that is conserved for any continuous deformation (homotopy),^[^ ^82^ ^]^ that is, stretching, twisting, crumpling, and bending. Any continuous normalized vector field l being defined on the geometrical surface could realize a map of the surface into a sphere *S* ^2^, where the degree of this map denotes an integer topological invariant (Pontryagin index)^[^ ^83^, ^84^ ^]^ *Q*, is also known as the topological charge.^[^ ^85^ ^]^ The degree *Q* of the map defined on a 2D curved shell reads^[^ ^33^, ^82^ ^]^ 
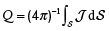
, where $dS=\sqrt{g}d\xi_{1}d\xi_{2}$ is the curvilinear surface element. In this particular case, the mapping Jacobian J can be presented in the form of the triple product:^[^ ^33^, ^82^ ^]^

(7) $$J=-\frac{1}{2}\frac{\varepsilon_{\alpha \beta }}{\sqrt{g_{\alpha \alpha }g_{\beta \beta }}}l\;\cdot \;\left[\left(\partial_{\alpha }l)\times (\partial_{\beta }l\right)\right]$$

The direction of the mapping l bypass on a sphere *S* ^2^ represents the chirality of the vector field *C*. Namely, the vector field is called chiral if it cannot be transformed to its mirror image by applying translate and rotate operations alone. Any two mappings with different *C* but with the same *Q* belong to the same homotopy class and they can be transformed into each other by means of a continuous deformation of the vector field.^[^ ^33^ ^]^

The discontinuity of the order parameter field l in the geometrical space is energetically unfavorable, which results in the formation of different solutions with a specific *Q* being separated by a high energy barrier, that causes topological stability.^[^ ^33^ ^]^ Remarkably, that for a strictly normal distribution of the vector field $l=\pm \hat{n}$ (Rodrigues–Gauß map) one obtains the well‐known^[^ ^82^, ^84^, ^86^, ^87^, ^88^ ^]^ result $J=\mp \tilde{K}$. Applying the Gauß–Bonnet theorem, we obtain the famous relation Q=∓(1−g) between the degree of the Rodrigues–Gauß map and genus g of the surface. Thus, *Q* = ±1 for a normal distribution of l on a sphere (hedgehog state),^[^ ^33^ ^]^ *Q* = 0 for a normal distribution on a torus,^[^ ^33^ ^]^ etc.

## 3 Curvilinear Ferromagnets

### 3.1 State of the Art

Far below the Curie temperature, the time evolution of the magnetization distribution in magnets can be described as the solution of the phenomenological Landau–Lifshitz–Gilbert (LLG) equation of motion (here and below in CGS units)^[^ ^90^, ^91^ ^]^

(8) $$\frac{\partial m}{\partial t}\;\;=\;\;\underset{\text{precession}}{\underset{︸}{\frac{\gamma_{0}}{M_{s}}m\;\times \;\frac{\delta E}{\delta m}}}\;\;+\;\;\underset{\text{damping}}{\underset{︸}{\alpha_{G}m\;\times \;\frac{\partial m}{\partial t}}}$$

where $m=M/M_{s}$ is the normalized magnetization vector, *M*_s_ is the saturation magnetization, γ_0_ > 0 is the electron's gyromagnetic ratio, *E* is the total energy, symbol δ means the variational derivative, and α_*G*_ is the Gilbert damping constant. The Gilbert's relaxation term^[^ ^91^ ^]^ is derived within the Lagrangian formalism and preserves the length of the magnetization. This is a formal possibility to write the equation of motion for the unit vector only. We note that other forms of the damping term in Equation (8) can be used. For example, it is convenient to describe the magnetization dynamics at finite temperatures using the Bloch relaxation term (Landau–Lifshitz–Bloch equation, see review^[^ ^92^ ^]^). The longitudinal relaxation is also important at the ultrafast dynamics induced, for example, by laser pulses, which can be addressed by the Bar'yakhtar's approach.^[^ ^93^, ^94^, ^95^, ^96^, ^97^ ^]^

The total magnetic energy *E* can include various contributions.^[^ ^98^, ^99^ ^]^ Here, we mention the most common energy terms relevant for the state of the art in curvilinear magnetism. The strongest interaction in common materials is the exchange interaction, which determines the type of magnetic ordering (e.g., ferro‐ or antiferromagnetic). At the spin level, it is described by the energy of neighboring spins, represented by vectors $S_{1,2}$ as $H_{12}=J_{12}S_{1}\;\cdot \;S_{2}$ with *J*_12_ being the exchange integral (with this definition, *J*_12_ < 0 for ferromagnets and *J*_12_ > 0 for antiferromagnets). The continuum counterpart of this Heisenberg Hamiltonian for $\left|M\right|=\text{const}$ penalizes spatial inhomogeneities of magnetic textures. It reads^[^ ^99^ ^]^

(9) $$E_{\text{ex}}=-A\int m\;\cdot \;\nabla^{2}mdr$$

with *A* ∝ *J*_12_ being the exchange stiffness and ∇^2^ being the Laplace operator. The exchange energy density can be written in alternative forms, for example, *w*_ex_ = ∑_*i* =*x*, *y*, *z*_(∇*m*_*i*_)^2^.

While the exchange energy does not determine the global orientation of the magnetization, the preferable direction of spins within the magnet is determined by weaker relativistic interactions. The phenomenological model of the anisotropy can be built using symmetry considerations.^[^ ^100^ ^]^ For example, the anisotropy energy of uni‐ and biaxial magnets reads^[^ ^99^, ^100^ ^]^

(10) $$E_{\text{an}}=\int \left[-K_{1}{\left(m\;\cdot \;e_{\text{an1}}\right)}^{2}+K_{2}{\left(m\;\cdot \;e_{\text{an2}}\right)}^{2}\right]dr$$

where *K*_1,2_ are the anisotropy constants and $e_{\text{an1,2}}$ are the anisotropy axes. If both constants are positive, then $e_{\text{an1}}$ and $e_{\text{an2}}$ are called the easy and hard axes (of magnetization), respectively. The hard axis also determines the so‐called easy plane of magnetization, perpendicular to $e_{\text{an2}}$. The value of the anisotropy constants is determined by the local surrounding of magnetic ions and includes spin‐orbit, magnetoelastic and other contributions. In this way, the spin‐orbit interaction determines the energetically preferable spin direction with respect to the crystallographic axes. At interfaces of ultrathin films, it can lead to the uniaxial anisotropy with the easy axis of magnetization perpendicular to the interface.^[^ ^101^ ^]^ The internal strain of the sample can induce or change the anisotropy. We note, that the effective anisotropic contributions can be also associated with symmetries of magnetic textures, for example, configurational anisotropy.^[^ ^102^ ^]^ As will be discussed in the following, the coordinate‐dependent anisotropy in curvilinear magnets can be created by means of strain engineering.^[^ ^23^ ^]^

In many practical cases, it is important to take into account the dipolar interaction between spins. Being relativistic, it can make a major contribution to the statics and dynamics of ferromagnetic textures. At the spin level, it is described by the Hamiltonian^[^ ^98^, ^99^ ^]^

(11) $$H_{\text{dip}}=\frac{g^{2}\mu_{B}^{2}}{2}\sum_{i\neq j}\left[\frac{S_{i}\;\cdot \;S_{j}}{r_{ij}^{3}}-3\frac{\left(S_{i}\;\cdot \;r_{ij})(S_{j}\;\cdot \;r_{ij}\right)}{r_{ij}^{5}}\right]$$

where *g* is the Landé factor, μ_B_ is the Bohr magneton and $r_{ij}$ is the radius‐vector between *i*th and *j*th spins. To determine its micromagnetic counterpart, it is convenient to replace spins in (11) by respective magnetic moments and split the sum into two terms, $H_{\text{dip}}^{\text{loc}}$ and $H_{\text{dip}}^{\text{nonloc}}$.^[^ ^103^, ^104^ ^]^

The “local” term $H_{\text{dip}}^{\text{loc}}$ represents a sum running over the vicinity of each spin within the spherical volume *V*_0_ whose radius is much larger than the interatomic distance and still significantly smaller than the characteristic length of the spatial variation of the magnetization. This sum is determined by the local crystallographic surrounding of each magnetic ion and for ferromagnets it can be taken into account within the common phenomenological description (10). Being strongly dependent on the local crystal symmetry, it can be a significant part of the total anisotropy in antiferromagnets,^[^ ^104^, ^105^ ^]^ see also Section 4.

The “nonlocal” term $H_{\text{dip}}^{\text{nonloc}}$ can be written in a continuous form as^[^ ^103^, ^104^ ^]^

(12) $$E_{\text{ms}}=-\frac{1}{2}\int \left[\frac{4\pi }{3}M^{2}+(M\cdot H_{d})\right]dr$$

where the magnetic field $H_{d}=-\nabla \Phi (r)$ is the demagnetizing field determined by the Maxwell magnetostatic equations with $\Phi (r)=\int d\tilde{S}(M(\tilde{r})\cdot \hat{n})/\left|r-\tilde{r}\right|-\int d\tilde{r}(\tilde{\nabla }\cdot M(\tilde{r}))/\left|r-\tilde{r}\right|$ being the magnetostatic potential, $\hat{n}=\hat{n}(\tilde{r})$ being the normal to the sample's surface and $\tilde{\nabla }$ being the nabla operator for $\tilde{r}$. The first term in Equation (12) can be omitted in case of constant magnetization length. The second term with the demagnetizing field can be in special cases reduced to the effective shape anisotropy^[^ ^106^, ^107^, ^108^ ^]^ (e.g., easy‐plane one), which recovers a local formulation of the total energy.

The properties and responses of low‐dimensional magnetic systems are strongly dependent on their structural and geometrical symmetries, due to the dominating influence of the sample topology and local geometrical curvatures on the 3D magnetization vector fields. In particular, systems with low structural symmetry and large spin‐orbit coupling (SOC) are crucial for magnetism as they have strong influence on both magnetic and electronic properties.^[^ ^109^, ^110^, ^111^ ^]^ For instance, the broken inversion symmetry in crystals^[^ ^112^, ^113^ ^]^ or layer stacks^[^ ^109^, ^110^, ^111^ ^]^ can lead to the stabilization of topologically nontrivial magnetic textures, including skyrmions^[^ ^114^, ^115^, ^116^ ^]^ and chiral domain walls.^[^ ^117^, ^118^ ^]^ Due to their appealing properties, for example, topological stability even at room temperatures,^[^ ^119^, ^120^, ^121^ ^]^ nanoscale size, dynamical properties,^[^ ^116^ ^]^ and low pinning,^[^ ^122^ ^]^ these chiral structures are in the heart of novel concepts for spin‐orbitronics,^[^ ^123^, ^124^ ^]^ oxitronics,^[^ ^125^, ^126^ ^]^ antiferromagnetic,^[^ ^127^ ^]^ and magnon spintronics.^[^ ^128^, ^129^, ^130^, ^131^ ^]^

The type of chiral effects as well as the magnetochiral properties are mainly determined by the intrinsic Dzyaloshinskii–Moriya interaction (DMI).^[^ ^109^, ^110^, ^111^, ^112^, ^113^ ^]^ This interaction arises in bulk magnetic crystals with a broken inversion symmetry^[^ ^112^, ^113^ ^]^ or at the interfaces between a ferromagnet and a nonmagnetic material with a strong SOC.^[^ ^109^, ^110^, ^111^ ^]^ The energy functional of the DMI can be written as a combination of the Lifshitz invariants $L_{i,j}^{\left(k\right)}=m_{i}\partial m_{j}/\partial k-m_{j}\partial m_{i}/\partial k$ with *i*, *j*, *k* = *x*, *y*, *z* (no summation over indices). In a particular case of ultrathin films of thickness *h*, it reads^[^ ^115^, ^132^ ^]^

(13) $$E_{\text{DMI}}=D\int \left[m_{z}(\nabla \;\cdot \;m)-(m\;\cdot \;\nabla )m_{z}\right]dr$$

where *D* ∝ 1/*h* is the Dzyaloshinskii constant and the film normal is directed along the $\hat{z}$ axis. At present, tailoring of the intrinsic DMI is done by optimizing materials either by doping bulk single crystals^[^ ^133^, ^134^ ^]^ or by adjusting interface properties of thin films and multilayer stacks.^[^ ^135^ ^]^ A viable alternative to the tuning of the intrinsic properties of materials aiming to impose or modify chiral responses relies on the break of the local inversion symmetry appearing in curvilinear structures of conventional materials.^[^ ^24^, ^136^, ^137^, ^138^ ^]^

In this respect, the combination of theoretical investigations, micromagnetic simulations, experimental fabrication, and characterization methods paves the way to a novel material science platform of designed artificial 3D structures with unique topological properties, that could be utilized in novel logic and memory devices. We refer the reader to excellent reviews summarizing the state of the art in curvilinear magnetism from fundamentals through fabrication to characterization of 3D architectures.^[^ ^9^, ^76^, ^136^, ^138^, ^139^, ^140^, ^141^, ^142^, ^143^, ^144^ ^]^ The perspective of the development of curvilinear magnetism is outlined in the 2017 and 2020 Magnetism Roadmaps.^[^ ^145^, ^146^ ^]^ In the following, we will summarize the most important predictions and methods to study magnetic samples with curvilinear geometry.

#### 3.1.1 Theoretical Studies

**Figure 2:**
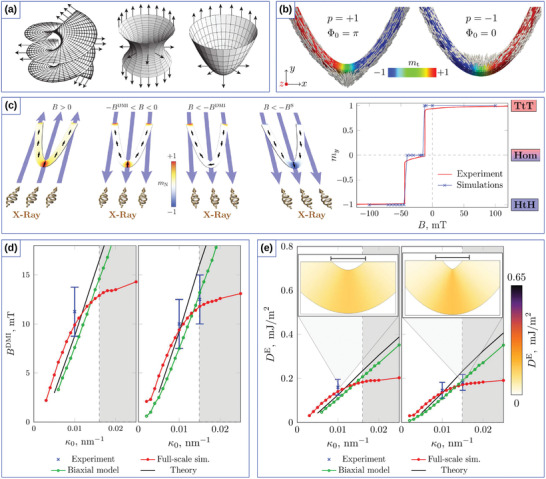
Exchange‐induced effects in simple curvilinear geometries. a) Spin configurations on different geometrical objects. Reproduced with permission.^[^ ^89^ ^]^ Copyright 2010, IOP Publishing. b) Domain wall pinning at localized wire bends. Reproduced with permission.^[^ ^37^ ^]^ Copyright 2015, American Physical Society. c) Schematic illustration of the four main magnetic states appearing during the field reversal: saturation, tail‐to‐tail domain wall (TtT), homogeneous state (Hom) along the parabola, and again saturation after the nucleation of a head‐to‐head domain wall (HtH) in the reversed field (right). The blue arrows mark the external field direction, the small black arrows indicate the magnetization direction. In color code, the component of the magnetization perpendicular to the parabola's long axis is shown. Reducing the field from the positive saturation to zero, a head‐to‐head domain wall is forming at the apex of the parabolic stripe. To remove the domain wall pinned by the curvature‐induced DMI at the apex, a certain negative field −*B* ^DMI^ has to be applied. Further increase of the field in the negative direction at *B* ^S^, results in the nucleation of a domain wall at the end of the parabola, which will move to the apex, thus reversing the magnetization. A comparison of the experimentally and numerically obtained hysteresis loops for parabolic stripe *W* = 135 nm κ_0_ = 0.015 nm^−1^ (left). The blue crosses correspond to the experimentally observed magnetic contrast change via XMCD‐PEEM imaging, see below. Red lines correspond to results of full‐scale micromagnetic simulations. d) Depinning fields as a function of the vertex curvature κ_0_ are shown for parabolic stripes with the widths of 125 and 135 nm, respectively. e) The dependencies of the exchange‐driven DMI *D* ^E^ constants on κ_0_ for parabolic stripes with the widths of 125 and 135 nm, respectively. c–e) Reproduced with permission.^[^ ^24^ ^]^ Copyright 2019, American Physical Society.

The research field of curvilinear magnetism investigates the influence of the object's geometry (i.e., curvature and torsion for quasi‐1D systems and principal curvatures for curved shells) on statics and dynamics of magnetic textures. In this respect, it addresses: (i) the interconnection between the magnetic and geometrical topologies, (ii) magnon propagation and excitation, and (iii) spin transport properties without taking into consideration the modification of the electronic band structure of the material. The first theoretical works, that considered curvature‐induced effects on magnetic systems, were simplified to the specific set of curvilinear geometries,^[^ ^89^, ^147^, ^148^, ^149^, ^150^, ^151^, ^152^, ^153^, ^154^, ^155^, ^156^, ^157^ ^]^ see **Figure** **2**a. In 2014, Gaididei et al.^[^ ^36^ ^]^ introduced a general theoretical approach to treat curvilinear effects in magnetic geometries of arbitrary shape for local micromagnetic interactions. Very recently, this approach was extended by Sheka et al.^[^ ^61^ ^]^ by taking into account curvature‐induced effects of nonlocal magnetostatic interaction. These studies established the theoretical curvilinear magnetism as a research field of the modern magnetism, which is studying curvature‐induced effects and their impact on magnetic responses.

It is instructive to show the influence of a curvilinear geometry on the magnetic system using a thin 1D nanowire as a case study. In the case when the magnetization is uniform along the cross‐section, the geometry of the magnetic wire can be described by a space curve γ(s). When taking into account the exchange and anisotropy interactions only, the transition to the local orthogonal curvilinear frame of references simplifies the description of the magnetic sample with curvilinear geometry, because the magnetic anisotropy term becomes translational invariant.

The geometrically broken symmetry of the curvilinear object leads to the reshaping of all energy terms containing spatial derivatives due to the coordinate dependence of the local curvilinear frame of reference. In the case of the exchange interaction with the TNB‐parameterization of the magnetization $m=m_{T}e_{T}+m_{N}e_{N}+m_{B}e_{B}$, one can rewrite the exchange energy as follows:^[^ ^158^ ^]^

(14) $$E_{\text{ex}}=S\int dsA\left[{m^{\prime }}_{\alpha }{m^{\prime }}_{\alpha }+F_{\alpha \beta }(m_{\alpha }{m^{\prime }}_{\beta }-{m^{\prime }}_{\alpha }m_{\beta })+K_{\alpha \beta }m_{\alpha }m_{\beta }\right]$$

where *S* is a cross‐section area of a wire. The first term is the isotropic part of the exchange, which formally has the same form as for a straight wire. The second term in Equation (14) is the chiral term, which has the form of the set of Lifshitz invariants and describes the geometrical symmetry breaking. This set of Lifshitz invariants in the curvilinear frame of reference can be referred to as a curvature‐induced DMI, which is linear with respect to the local curvature κ(*s*) and torsion τ(*s*). The third term in Equation (14) describes the curvature‐induced anisotropy. The corresponding coefficients are determined by the components of the tensor *K*_*αβ*_ = *F*_*αν*_ *F*_*βν*_. They are bilinear with respect to κ(*s*) and τ(*s*):

(15) $$\left\|K_{\alpha \beta }\right\|=\left\|\begin{array}{ccc} \kappa {\left(s\right)}^{2} & 0 & -\kappa (s)\tau (s)\\ 0 & \kappa {\left(s\right)}^{2}+\tau {\left(s\right)}^{2} & 0\\ -\kappa (s)\tau (s) & 0 & \tau {\left(s\right)}^{2} \end{array}\right\|$$

It is also convenient to represent the exchange energy Equation (14) in the following form:

(16) $$\begin{array}{c} E_{\text{ex}}=S\int dsA{m^{\prime }}_{\alpha }{m^{\prime }}_{\alpha }-S\int ds\varepsilon_{\alpha \beta \gamma }D_{\alpha }^{E}(s)m_{\beta }{m^{\prime }}_{\gamma }\\ +\;S\int dsA{\left[\tau (s)m_{T}+\kappa (s)m_{B}\right]}^{2} \end{array}$$

where the property $\left|m\right|=1$, ε_*αβγ*_ is the Levi–Civita symbol and Frenet–Serret formulae are utilized. In the Equation (16), vector $D^{E}(s)=-2A\tau (s)e_{T}-2A\kappa (s)e_{B}$ denotes the reduced vector of the extrinsic curvature‐driven DMI.^[^ ^36^, ^158^, ^159^ ^]^ In contrast to the intrinsic DMI, whose strength is dependent on the strength of the SOC, the amplitude of the extrinsic DMI is determined by local curvatures.^[^ ^24^, ^34^ ^]^ The vector of the extrinsic DMI is always lying in the rectifying plane to a curved geometry.^[^ ^160^ ^]^ This results in the appearance of local pinning potentials and thus to the localization of spin textures at the bending regions of wires^[^ ^37^ ^]^ (Figure 2b), geometrically induced motion of magnetic domain walls in curved nanostripes,^[^ ^161^, ^162^ ^]^ asymmetric spin‐wave dispersions in helical magnetic nanowires,^[^ ^159^ ^]^ local magnon modes in curved magnetic nanowires,^[^ ^163^ ^]^ which leads to the creation of curvature‐induced magnonic crystals^[^ ^164^ ^]^ and to the appearance of geometrical or Berry phases.^[^ ^128^, ^165^ ^]^

Despite that the described curvature‐induced chiral effects stemming from the exchange interaction are generic, there were challenges in their rigorous experimental confirmation because these geometry‐related effects can be shadowed by other effects or interactions. Only recently the curvature‐induced chiral responses were reported experimentally in flat parabolic stripes made from a soft ferromagnetic material (Permalloy).^[^ ^24^ ^]^ Such system is not only the mathematically simplest possible curve with well‐defined geometrical parameters, but also contains a shape‐induced anisotropy, which reflects the parabolic shape due the magnetostatic or dipole–dipole interaction. It was predicted theoretically that the magnetization reversal of flat parabolic stripes shows a two‐step process due to the presence of curvature‐induced exchange‐driven DMI, see Figure 2c. The first switching event occurs due to the expelling of the transversal domain wall from a pinning potential induced by the curvature‐induced DMI stemming from the exchange interaction, while the second one corresponds to the nucleation of another transversal domain wall with the opposite chirality. Experimental investigations of static magnetization states appearing during the hysteresis loops confirmed the predicted switching mechanism. Supporting these experimental results with micromagnetic simulations and analytical calculations, it became possible to quantify the strength of the curvature‐induced DMI constant *D* ^E^ based on the analysis of the first switching field *B* ^DMI^, see Figure 2c:

(17) $$D^{E}=B^{\text{DMI}}M_{s}l\sqrt{\frac{2\pi W}{h\left[2\ln(W/h)+3\right]}}$$

where ℓ is the exchange length, with *W* and *h* being stripe width and thickness, respectively. The curvature‐induced DMI was found to be remarkably strong and in its strength comparable to the surface‐induced DMI in asymmetric Co sandwiches. Furthermore, the strength of the curvature‐induced DMI can be tuned by tailoring the local curvature of the parabola, see Figure 2d.

The geometrically broken symmetry also implies the restructuring of the spatial derivatives in the case of the intrinsic DMI, which for the case of a curvilinear nanowire reads^[^ ^160^ ^]^

(18) $$E_{\text{DMI}}=-S\int dsD^{I}\cdot [m\times m^{\prime }]$$

where $D^{I}=D_{T}^{I}e_{T}+D_{N}^{I}e_{N}+D_{B}^{I}e_{B}$ is the intrinsic DMI vector in the TNB reference frame. It should be noted, that in a general case, its direction can be arbitrary. The geometrically broken symmetry leads to the appearance of an additional DMI‐induced anisotropy:^[^ ^33^, ^160^ ^]^

(19) $$E_{\text{DMI}}=S\int ds\left[F_{\alpha \beta }^{\text{DMI}}(m_{\alpha }{m^{\prime }}_{\beta }-{m^{\prime }}_{\alpha }m_{\beta })+K_{\alpha \beta }^{\text{DMI}}m_{\alpha }m_{\beta }\right]$$

Here, the first term describes the intrinsic DMI in a curvilinear magnetic system with the following chiral tensor

(20) $$\left\|F_{\alpha \beta }^{\text{DMI}}\right\|=\left\|\begin{array}{ccc} 0 & -D_{B}^{I}/2 & D_{N}^{I}/2\\ D_{B}^{I}/2 & 0 & -D_{T}^{I}/2\\ -D_{N}^{I}/2 & D_{T}^{I}/2 & 0 \end{array}\right\|$$

The second term corresponds to the DMI‐induced anisotropy and contains the following anisotropy tensor

(21) $$\begin{array}{l} \left\|K_{\alpha \beta }^{\text{DMI}}\right\|=\|\begin{array}{cc} -D_{\text{B}}^{\text{I}}\kappa (s) & D_{\text{N}}^{\text{I}}\tau (s)\text{/}2\\ D_{\text{N}}^{\text{I}}\tau (s)\text{/}2 & -D_{\text{B}}^{\text{I}}\kappa (s)-D_{\text{T}}^{\text{I}}\tau (s)\\ \left[D_{\text{T}}^{\text{I}}\kappa (s)+D_{\text{B}}^{\text{I}}\tau (s)\right]\text{/}2 & D_{\text{N}}^{\text{I}}\kappa (s)\text{/}2 \end{array}\\ \begin{array}{c} \left[D_{\text{T}}^{\text{I}}\kappa (s)+D_{\text{B}}^{\text{I}}\tau (s)\right]\text{/}2\\ D_{\text{N}}^{\text{I}}\kappa (s)\text{/}2\\ -D_{\text{T}}^{\text{I}}\tau (s) \end{array}\| \end{array}$$

The interplay between the intrinsic spin‐orbit‐driven and the extrinsic curvature‐driven DMI terms leads to the appearance of the so‐called mesoscale DMI.^[^ ^160^ ^]^ In the case of 1D helimagnetic curvilinear wires, it has the following form:^[^ ^160^ ^]^

(22) $$D=D^{I}+D^{E}(s)=\left[D_{T}^{I}-2A\tau (s)\right]e_{T}+D_{N}^{I}e_{N}+\left[D_{B}^{I}-2A\kappa (s)\right]e_{B}$$

where D is a vector of the mesoscale DMI. It is defined by a vector sum of the DMI vectors of the intrinsic and the extrinsic types, respectively. Thus, for a curved 1D object, the vector D determines a new direction of the effective DMI in the system. It should be emphasized that D is dependent on the both geometrical and material properties of the sample.

It is instructive to illustrate this general approach to the mesoscale DMI using a specific example of a helical wire and compare these results to the case of a straight wire with the same micromagnetic properties. A helix is the simplest curvilinear system with both non‐zero curvature and torsion. It can be parameterized as $\gamma (s)=\hat{x}R\cos(s/s_{0})+\hat{y}R\sin(s/s_{0})+\hat{z}CPs/(2\pi s_{0})$. Here, *R* is the helix radius, *P* is the pitch of the helix, C=±1 is the helix chirality, namely, C=−1 for the clockwise (right) helix and C=1 for the counter‐clockwise (left) one and $s_{0}=\sqrt{R^{2}+P^{2}/{\left(2\pi \right)}^{2}}$. The helix is characterized by the constant curvature $\kappa =R/s_{0}^{2}$ and torsion $\tau =CP/(2\pi s_{0}^{2})$.

**Figure 3:**
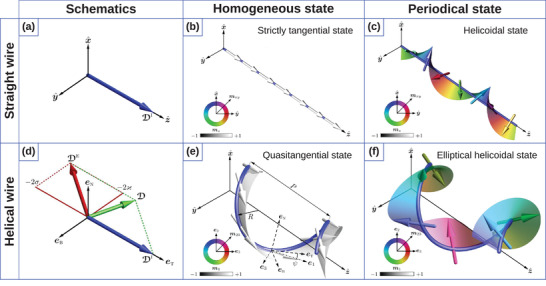
Schematic illustration of the interplay between the intrinsic and extrinsic DMI and the resulting magnetization distributions in a wire. a) The vector of *i*DMI in the Cartesian frame of reference for a straight wire. b,c) Tangential homogeneous (κ = 0, τ = 0, $D_{T}^{I}=0$) and periodical helicoidal (κ = 0, τ = 0, $D_{T}^{I}=2.7$) states in a straight wire with the easy‐tangential anisotropy and *i*DMI. d) Vectors of the *i*DMI and *e*DMI in the TNB reference frame. e,f) Quasitangential (κ = 0.8/l, τ = 0.5/l, $D_{T}^{I}=0$, C=+1) and periodical (κ = 0.8/l, τ = 0.5/l, $D_{T}^{I}=2.7$, C=+1) states in a helical wire with the easy‐tangential anisotropy, with $l=\sqrt{A/K}$ being the magnetic length. Color arrows correspond to the magnetic moments. The Cartesian, the Frenet–Serret $\left\{e_{T},e_{N},e_{B}\right\}$ and the rotated $\left\{e_{1},e_{2},e_{3}\right\}$ reference frames are shown with solid, dashed and dashed‐dot lines, respectively in (e). a–f) Reproduced under the terms of the CC‐BY Creative Commons Attribution 4.0 International license (https://creativecommons.org/licenses/by/4.0).^[^ ^160^ ^]^ Copyright 2018, The Authors, published by Springer Nature.

Let us consider a homogeneous intrinsic DMI with the vector along the tangential direction of a wire. Namely, $D^{I}=D_{T}^{I}\hat{z}$ for the straight wire, which is similar to those in a cubic non‐centrosymmetric magnets,^[^ ^166^ ^]^ **Figure** **3**a, and $D^{I}=D_{T}^{I}e_{T}$ for the helix wire. In the latter case, the vector of the mesoscale DMI $D=(D_{T}^{I}-2A\tau )e_{T}-2A\kappa e_{B}$ lies in the TB‐plane, Figure 3d. Note, that for the specific value of the torsion $\tau =D_{T}^{I}/(2A)$ the direction of the mesoscale DMI vector becomes perpendicular to the initial tangential direction. This can be interpreted as the change of the type of the DMI from the bulk one to the interfacial one.^[^ ^167^ ^]^

In the case of a weak intrinsic DMI or mesoscale DMI, in both geometries homogeneously magnetized states will appear, see Figure 3b,e. However, in the case of a straight wire the equilibrium homogeneous state will be always tangential, while in the case of a helical wire, the magnetization will always have a tilt, resulting in a quasitangential state. It should be noted that a magnetization tilt is present even in helical wires with strong tangential anisotropy, due to the curvature‐induced anisotropies, see Figure 3e. In the case of a strong DMI, a simple helicoidal magnetization state (Figure 3c) will develop in a straight wire. In contrast, in the case of a helical wire, the equilibrium state will be an elliptical helicoidal, due to the influence of the mesoscale DMI, which imposes a new direction of the magnetochirality,^[^ ^160^ ^]^ see Figure 3f.

Curvature‐induced effects are not only specific for 1D curvilinear magnetic systems, but also appear in 2D curvilinear magnetic shells. The general description of curvilinear magnetic shells utilizes formalism of covariant derivatives to separate effects of geometry from peculiarities of the symmetry of the reference frame itself. It is convenient to introduce the so‐called tangential derivatives:^[^ ^61^ ^]^

(23) $${ð}_{\alpha }m_{\beta }=\frac{\partial_{\alpha }m_{\beta }+\varepsilon_{\beta \gamma }\left(e_{1}\cdot \;\partial_{\alpha }e_{2}m_{\gamma }\right)}{\sqrt{g_{\alpha \alpha }}},\;\;{ð}_{\alpha }m_{n}=\frac{\partial_{\alpha }m_{n}}{\sqrt{g_{\alpha \alpha }}},\;\;\alpha ,\beta =1,2$$

where $m=m_{\alpha }e_{\alpha }+m_{n}\hat{n}$ is the unit magnetization vector in a curvilinear frame of references, *g*_*αβ*_ is the metric tensor and ε*_βγ_* is the totally antisymmetric tensor. Expression (23) allows to collect all terms, related to the symmetry of the in‐surface reference frame (e.g., cylindrical‐like one) within the symbol ð_α_ regardless of principal curvatures. Here, ð_α_ *m*_β_ is the covariant derivative of the in‐plane magnetization components and ð_α_ *m*_*n*_ is the common derivative of the normal component of magnetization with taking into account metrics ‖*g*_*αβ*_‖ of the surface.

In terms of the definition (23), the exchange interaction acquires the additional anisotropic and chiral parts, in a way, similar to nanowires:^[^ ^61^ ^]^

(24) $$\begin{array}{l} E_{\text{ex}}=h\int dSA[\underset{\text{Regular}}{\underset{︸}{\left({ð}_{\alpha }m_{i})({ð}_{\alpha }m_{i}\right)}}\\ +\underset{\text{Chiral part}}{\underset{︸}{2\kappa_{\alpha }(m_{i}{ð}_{\alpha }m_{j}-m_{j}{ð}_{\alpha }m_{i})}}+\underset{\text{Anisotropic part}}{\underset{︸}{K_{ij}m_{i}m_{j}}}] \end{array}$$

where *i*, *j* = 1, 2, *n* denote the components of a vector field. The first term in the exchange energy Equation (24) represents the regular isotropic part, the second one defines a chiral part and the last one denotes a curvature‐induced biaxial anisotropy with $\left\|K_{ij}\right\|=\text{diag}(\kappa_{1}^{2},\kappa_{2}^{2},\kappa_{1}^{2}+\kappa_{2}^{2})$. The curvature‐induced DMI in the case of thin tubular shells leads to the modification of the critical DMI value and appearance of a novel type of inclined domain walls.^[^ ^170^ ^]^

**Figure 4:**
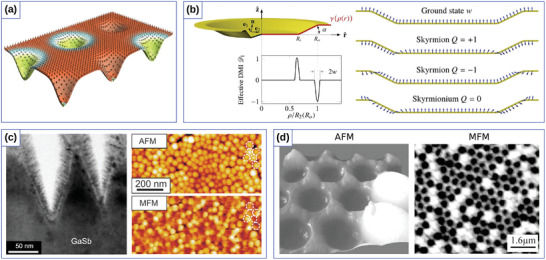
Exchange‐induced curvilinear effects of curved thin shells. a) Schematic representation of two skyrmion states with big and small radii, stabilized by principal curvatures in a square lattice of Gaussian concave bumps. Reproduced with permission.^[^ ^38^ ^]^ Copyright 2018, American Physical Society. b) Sample geometry and magnetization patterns stabilized by the gradient of curvature. Reproduced with permission.^[^ ^39^ ^]^ Copyright 2018, American Physical Society. c) Cross‐section TEM, AFM and MFM images of the geometrically stabilized magnetic states in CoCrPt:SiO_2_ thin films deposited on corrugated template. Reproduced with permission.^[^ ^168^ ^]^ Copyright 2014, IOP Publishing. d) AFM and MFM images of magnetic spherical indentations that reveal curvature‐stabilized magnetic states. Reproduced with permission.^[^ ^169^ ^]^ Copyright 2007, AIP Publishing.

Similarly to the 1D case, 2D curvilinear shells also allow to stabilize nontrivial magnetic textures due to the curvature‐induced DMI stemming from the exchange interaction. Namely, it was predicted theoretically that a chiral Néel skyrmion can form as a ground state when its radius is comparable with the size of curvilinear defect,^[^ ^38^ ^]^ see **Figure** **4**a. Thus, it is possible to stabilize magnetic skyrmions at local maxima or minima of principal curvatures without any intrinsic chiral interactions.^[^ ^33^, ^38^ ^]^ Also, it was demonstrated that, even in the absence of an intrinsic DMI, the gradient of the local curvature is an efficient means to stabilize chiral localized magnetic objects, allowing to manipulate their size at will.^[^ ^39^ ^]^ Engineering the geometry of a circular nanoindentation to have a defined curvature and distance between bends allows one to form skyrmion states with winding numbers *Q* = ±1 and skyrmionium states with *Q* = 0, whose diameter is determined by the diameter of the nanoindentation, see Figure 4b. Interesting to note, that in contrast to the planar case, where the skyrmion solution has |*Q*| = 1, on a spherical shell the skyrmion state is topologically trivial with *Q* = 0.^[^ ^33^ ^]^ In addition, the localized curvature of a thin shell influences the spin‐wave spectrum of magnetic skyrmions by inducing modes with higher azimuthal numbers, that are absent in rectilinear films.^[^ ^171^ ^]^ The translational mode of the skyrmion is transformed into the gyromode by the curvature and has a nonzero frequency proportional to the second derivative of the mean curvature at the bump center.^[^ ^171^ ^]^

These theoretical predictions are in line with experimental studies of magnetic responses from multilayer thin films deposited on the corrugated templates.^[^ ^101^, ^168^, ^169^, ^172^ ^]^ Namely, self‐organized patterns of GaSb nanocones induced by the ion erosion, see Figure 4c, and spherical indentations, see Figure 4d, were used as templates for subsequent deposition of magnetic films with out‐of‐plane anisotropy. Investigations of magnetic states and structure topography revealed a strong correlation between the position of magnetic textures and spatial corrugations. In this respect, these experimental geometries reveal a promising playground for the validation of the theoretical predictions of the formation of skyrmion and skyrmionium states.

Following the formalism of the tangential derivatives (Equation (23)),^[^ ^61^ ^]^ it is possible to build the micromagnetic theory of curvilinear shells.^[^ ^61^ ^]^ To rewrite the magnetostatic energy in the translationally invariant form of its magnetization multipliers in the magnetostatic potential, it is convenient to restructure the common volume magnetostatic charge as follows:^[^ ^61^ ^]^

(25) $$-\nabla \;\cdot \;m=\rho (r)+g(r),\;\;\rho (r)=-{ð}_{\alpha }m_{\alpha },\;\;g(r)=\left[\kappa_{1}(r)+\kappa_{2}(r)\right]m_{n}$$

Terms ρ and *g* represent the so‐called tangential and geometrical magnetostatic charges, respectively. Note, that the latter one is determined by the mean curvature of the shell. This geometrical charge leads to the restructuring of the magnetostatic energy as follows:^[^ ^61^ ^]^

(26) $$\begin{array}{l} E_{\text{ms}}=M_{s}^{2}\int dSw_{\sigma \sigma }(r)+M_{s}^{2}\int drw_{\rho \rho }(r)\\ +\;M_{s}^{2}\int dr\left[w_{gg}(r)+w_{g\sigma }(r)+w_{g\rho }(r)+w_{\sigma \rho }(r)\right] \end{array}$$

Here, the first term represents the interaction between the surface charges $\sigma^{\pm }=m\;\cdot \;{\hat{n}}^{\pm }$ at the top and bottom surfaces of the shell, while the second term represent the interaction between tangential charges:

(27) $$w_{\sigma \sigma }(r)=\frac{\sigma (r)}{2}\int dS^{\prime }\frac{\sigma (r\prime )}{\left|r-r\prime \right|},\;\;w_{\rho \rho }(r)=\frac{\rho (r)}{2}\int dr\prime \frac{\rho (r\prime )}{\left|r-r\prime \right|}$$

Thus, *w*_ρρ_ formally has the same form as the energy of volume magnetostatic charges in flat films. In addition to these standard terms, the new geometrical charge possesses the self‐interaction and interact with the surface charges:

(28) $$w_{gg}(r)=\frac{g(r)}{2}\int dr\prime \frac{g(r\prime )}{\left|r-r\prime \right|},\;\;w_{g\sigma }(r)=g(r)\int dS^{\prime }\frac{\sigma (r\prime )}{\left|r-r\prime \right|}$$

where both these terms provide nonlocal coupling between normal magnetization components. The interaction between the geometrical and tangential charges

(29) $$w_{g\rho }(r)=\rho (r)\int dS^{\prime }\frac{g(r\prime )}{\left|r-r\prime \right|}$$

favors the coupling between the normal magnetization *m*_*n*_ and spatial derivatives of the in‐surface components providing a chiral contribution to the energy functional. The last magnetostatic term of the similar symmetry

(30) $$w_{\sigma \rho }(r)=\rho (r)\int dS^{\prime }\frac{\sigma (r\prime )}{\left|r-r\prime \right|}$$

is caused by the interaction between surface and tangential charges, that appear in any curvilinear surface due to the difference in the area of the inner and outer surfaces. Note, that the magnetostatic kernel $1/\left|r-r\prime \right|$ does not involve the geometrical invariants of the shell explicitly.

**Figure 5:**
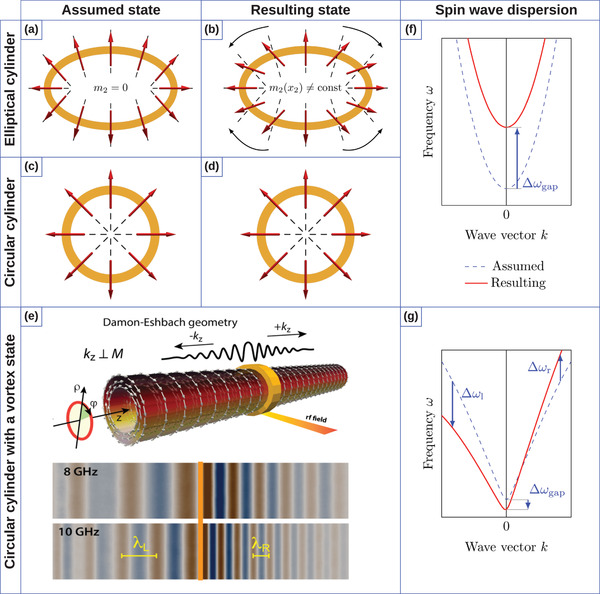
Schematics of the modification of the assumed equilibrium state and spin wave spectrum by curvature. a–d) Elliptic and circular cylinders with easy‐normal anisotropy affected by the curvature in different ways: magnetization pattern in the elliptic cylinder is modified due to the symmetry breaking. Magnetization is shown by the red arrows, normal direction is shown by the dashed lines. e) Curvature‐induced asymmetric spin‐wave dispersion in magnetic nanotubes. f,g) The dispersion of spin waves propagating along the cylinder axis $e_{1}$ is shown by black arrow in panel (e). While the dispersion curve is only shifted by Δω_gap_ for the case of easy‐normal anisotropy due to the contribution from exchange‐induced anisotropy term, it also becomes asymmetric for the vortex state due to *w*_gρ_ and *w*_σρ_.^[^ ^173^, ^174^ ^]^ The blue arrows show the change from the assumed to the actual dispersion curve with Δω_l_ and Δω_r_ being the frequency shifts for the left and right branches of the lowest radially symmetric mode. a–d,f,g) Reproduced under the terms of the CC‐BY Creative Commons Attribution 4.0 International license (https://creativecommons.org/licenses/by/4.0).^[^ ^61^ ^]^ Copyright 2020, The Authors, published by Springer Nature. e) Adapted with permission.^[^ ^173^ ^]^ Copyright 2016, American Physics Society.

The influence of the terms in Equation (26) is determined by the geometrical class of the surface (e.g., developable with one of the principal curvatures being trivial, or minimal with zero mean curvature) and symmetry of the magnetic texture. The terms (29) and (30) with the geometrically driven symmetry break in the magnetostatic energy lead to the static and dynamic nonlocal magnetochiral effects.^[^ ^61^, ^136^, ^173^, ^174^ ^]^ Developable surfaces include the family of circular and elliptic cylinders with κ_1_ = 0 and κ_2_ ≠ 0. The effect of curvature on the magnetic state can be illustrated for the case of the strong easy‐normal anisotropy. The latter forces to expect the magnetization being normal to the surface $\tilde{m}=\hat{n}$ (assumed state) as the first approximation. It is affected by the chiral exchange term, which includes κ_2_, and magnetostatic ones *w*_*g*ρ_ and *w*_σρ_, see **Figure** **5**a,b. The resulting equilibrium state shows an additional tilt of the magnetization to the normal direction due to the variation of the curvature, *∂*_2_. Such considerations allow to conclude that the assumed state along the normal direction will remain for the circular cylinder with κ_2_ = const, see Figure 5c,d.

This approach can be also used to describe the propagation of spin waves along the straight generatrix $e_{1}$ in the equilibrium state of cylinders. The analysis of the energy terms predicts the reciprocal propagation for the both cases of elliptic and circular cylinders: the exchange‐induced anisotropy as well as the magnetostatic terms *w*_gg_ and *w*_gσ_ are responsible for the shift of a magnon gap Δω_gap_, see Figure 5f. It should be noted that this effect is similar to the magnon gap shift for vortex domain walls in circular cylinders.^[^ ^175^ ^]^

**Figure 6:**
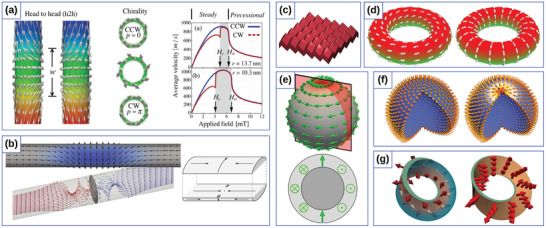
Theoretical curvilinear magnetism. a) Chirality symmetry breaking for vortex domain walls on cylindrical nanotube. Reproduced with permission.^[^ ^177^ ^]^ Copyright 2012, AIP Publishing. b) Bloch‐point domain wall in nanotubes. Reproduced with permission.^[^ ^181^ ^]^ Copyright 2014, American Physical Society. c) Perpendicular anisotropy engineered by periodic surface structures. Reproduced with permission.^[^ ^209^ ^]^ Copyright 2017, American Physical Society. d) Topologically defined magnetic states for toroidal nanoshells. Reproduced with permission.^[^ ^183^ ^]^ Copyright 2019, Elsevier. e) Topologically stabilized double‐vortex state on a spherical shell. Reproduced with permission.^[^ ^184^ ^]^ Copyright 2017, Elsevier. f) Topologically stable magnetization states on a spherical shell. Reproduced with permission.^[^ ^33^ ^]^ Copyright 2016, American Physical Society. g) Topologically protected domain walls in a Möbius band. Reproduced under the terms of the CC‐BY Creative Commons Attribution 3.0 Unported license (https://creativecommons.org/licenses/by/3.0).^[^ ^31^ ^]^ Copyright 2015, published by American Physics Society.

The prominent results attributed to the magnetochiral effects of dipolar origin are found in circular cylinders and curved magnetic membranes with the anisotropy along the $e_{2}$. The equilibrium state in this case is a vortex state, which follows the azimuthal anisotropy direction. In such systems there are no chiral effects for the equilibrium state. Still, in magnetization dynamics in nanotubes, there appear a curvature‐induced magnon gap Δω_gap_ and asymmetric spin‐wave dispersion,^[^ ^173^, ^174^ ^]^ due to the exchange‐induced anisotropy as well as magnetostatic terms *w*_gρ_ and *w*_σρ_, see Figure 5g. The magnetochiral effects also attribute: the decay length asymmetry of spin‐waves in curved magnetic membranes,^[^ ^176^ ^]^ chiral symmetry breaking of vortex domain wall motion in cylindrical nanotubes^[^ ^177^, ^178^, ^179^ ^]^ (**Figure** **6**a), chirality‐dependent vortex core switching on spherical caps^[^ ^180^ ^]^ and appearance of Bloch‐point domain walls in nanotubes^[^ ^181^ ^]^ (Figure 6b).

Based on the generalized theory of curvilinear magnetism,^[^ ^61^ ^]^ it was shown that all these effects do not rely on any specific modification of the intrinsic magnetic material properties and are always present in curvilinear systems. Moreover, the coupling of chiralities in spin space with the nontrivial topology of the physical space can lead to the appearance of magnetochiral effects in curvilinear magnetic systems, for example, topologically defined magnetic states for toroidal^[^ ^182^, ^183^ ^]^ (Figure 6d) and spherical^[^ ^33^, ^155^, ^184^ ^]^ nanoshells (Figure 6e,f), and topologically protected domain walls in Möbius stripes^[^ ^31^ ^]^ (Figure 6g).

#### 3.1.2 Computer Simulations

These theoretical explorations have been always complemented by numerical simulations. The micromagnetic simulations are unique and powerful tool to study equilibrium magnetization states, their dynamics and responses to external perturbations (e.g., magnetic fields or spin‐polarized currents) for arbitrary geometries, and in a wide range of time‐ and length‐scales. The micromagnetic simulation packages are mostly based on the numerical solution of the LLG equation of motion, either using a finite difference method (FDM, e.g., OOMMF^[^ ^185^, ^186^ ^]^ and MuMax^3^^[^ ^187^, ^188^ ^]^ for the submicrometer scale simulations and SLaSi,^[^ ^189^, ^190^ ^]^ Vampire^[^ ^191^, ^192^ ^]^ and Spirit^[^ ^193^, ^194^ ^]^ for atomistic simulations) or a finite element method (FEM, e.g., COMSOL Multiphysics^[^ ^195^ ^]^ with the LLG extension,^[^ ^196^ ^]^ Micromagnum,^[^ ^197^ ^]^ magnum.fe,^[^ ^198^, ^199^ ^]^ magpar,^[^ ^200^, ^201^ ^]^ FastMag,^[^ ^202^, ^203^ ^]^ Nmag^[^ ^204^, ^205^, ^206^ ^]^ and TetraMag^[^ ^207^ ^]^). While the FDM approach is faster than the FEM and it could be used for the simulation of flat 2D curvilinear geometries, the FDM approach introduces errors and artifacts that can hinder the curvature‐induced effects in the case of 3D curvilinear geometries, due to the step‐like boundaries. The magnetic samples with a general 3D geometry should be simulated using FEM approaches with a large number of discretization elements, especially when a precise reconstruction of the full‐scale 3D curvilinear system is required or the magnetization dynamics is in the focus of study. To significantly increase the calculation speed the massively parallel computation are used together with high‐performance graphics processing units (GPUs), which results in large speedups of both FDM^[^ ^188^, ^208^ ^]^ and FEM‐based^[^ ^207^ ^]^ computations and are widely used to simulate complex curvilinear architectures.

#### 3.1.3 Fabrication Methods

**Figure 7:**
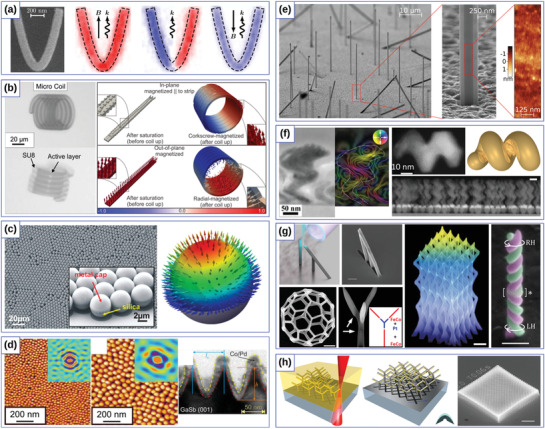
Experimental realization of curved magnetic objects. a) Parabolic stripe prepared by means of electron‐beam lithography. b) Rolled‐up magnetic tubular and helical architectures. c) Self‐assembled spherical particles capped with [Co/Pt]_8_ multilayer stack with an easy‐normal magnetic state. d) 3D template with GaSb nanocones coated with Co/Pd multilayer. e) Free‐standing magnetic hexagonal nanotubes prepared from single crystalline GaAs cores. f) Array of magnetic nanohelices made by means of GLAD technique. g) 3D self‐standing magnetic stripe by means of FEBID. h) Two‐photon fabrication methodology and resulting structures. a) Reproduced with permission.^[^ ^24^ ^]^ Copyright 2019, American Physical Society. b) (left part) Reproduced with permission.^[^ ^238^ ^]^ Copyright 2011, Royal Society of Chemistry. (right part) Reproduced with permission.^[^ ^23^ ^]^ Copyright 2011, American Physical Society. c) Reproduced with permission.^[^ ^45^ ^]^ Copyright 2012, American Chemical Society. d) Reproduced with permission.^[^ ^101^ ^]^ Copyright 2017, IOP Publishing. e) Reproduced with permission.^[^ ^239^ ^]^ Copyright 2018, American Chemical Society. f) (left part) Reproduced with permission.^[^ ^240^ ^]^ Copyright 2014, American Chemical Society. (right part) Reproduced with permission.^[^ ^47^ ^]^ Copyright 2014, Royal Society of Chemistry. g) (left top part) Adapted under the terms of the ACS Author Choice with CC‐BY license (https://pubs.acs.org/page/policy/authorchoice_ccby_termsofuse.html).^[^ ^241^ ^]^ Copyright 2017, American Chemical Society. (left bottom part) Reproduced with permission.^[^ ^242^ ^]^ Copyright 2018, The Authors, published by Beilstein‐Institut. (middle part) Reproduced with permission.^[^ ^278^ ^]^ Copyright 2017, American Chemical Society. (right part) Reproduced with permission.^[^ ^244^ ^]^ Copyright 2020, American Chemical Society. h) Reproduced under the terms of the CC‐BY Creative Commons Attribution 4.0 International license (https://creativecommons.org/licenses/by/4.0).^[^ ^245^ ^]^ Copyright 2019, The Authors, published by Springer Nature.

The appealing theoretical predictions together with potential applications in logic, memory and sensor devices^[^ ^210^, ^211^ ^]^ determine the recent rapid development of fabrication methods for the needs of curvilinear ferromagnetism. Flat curvilinear systems are prepared with standard lithographic techniques combined with thin film deposition. This allows to achieve high spatial resolution and ensures shape retention of the patterned geometry (**Figure** **7**a). This approach gave the opportunity to study magnetic processes in curved nanostripes^[^ ^212^, ^213^, ^214^ ^]^ and nanorings^[^ ^18^, ^215^, ^216^, ^217^, ^218^ ^]^ to address domain wall dynamics and automotion^[^ ^18^ ^]^ for prospective memory^[^ ^210^, ^211^, ^219^ ^]^ and logic^[^ ^14^, ^220^, ^221^, ^222^ ^]^ devices, as well as the concept of magnon‐based processing of binary data.^[^ ^131^, ^223^, ^224^, ^225^, ^226^ ^]^ The lithographic capabilities could be extended into 3D by utilizing strain engineering.^[^ ^227^, ^228^ ^]^ Once the deposited film is released from the substrate, mechanical strains tend to roll it up with a subsequent formation of micrometer‐size swiss‐rolls,^[^ ^23^, ^227^, ^228^ ^]^ that reveal significantly enhanced magnetoresistive/magnetoimpedance responses.^[^ ^229^ ^]^ Microtubes are explored as spin‐wave filters,^[^ ^230^, ^231^, ^232^ ^]^ magnetic sensors on fluids^[^ ^233^ ^]^ and catalytic jet engines.^[^ ^234^ ^]^ The fabrication of lithographic pattern before the rolling‐up procedure allows to fabricate microhelices of magnetic materials with different properties,^[^ ^23^, ^49^, ^235^ ^]^ see Figure 7b, that could be used as magnetic field sensors for magnetofluidic applications,^[^ ^49^, ^236^ ^]^ ratchet devices^[^ ^23^, ^237^ ^]^ and artificial helimagnets.^[^ ^23^ ^]^

An alternate way of obtaining controlled and scalable fabrication of 3D curvilinear nanostructures is the utilization of the curvature templates with the subsequent thin film coatings. Typically, spherical particles^[^ ^45^, ^169^, ^246^, ^247^ ^]^ (Figure 7c) and nanocylinders^[^ ^235^, ^248^ ^]^ are used as templates for the anisotropic coating, when only some part of the curvilinear structure is magnetically functionalized. This allows to study the formation of dipolar‐induced arrangement of out‐of‐plane^[^ ^249^ ^]^ and in‐plane magnetized^[^ ^9^, ^235^, ^250^ ^]^ thin magnetic shells and realization of the magnetically controlled Janus micromotors,^[^ ^45^, ^251^, ^252^, ^253^ ^]^ investigations of magnetoresistive effects on the magnetically capped monolayer of spherical particles.^[^ ^254^ ^]^

Curvilinear templates can be also formed by means of erosion‐based methods, which include the incident ion erosion of substrates^[^ ^255^, ^256^ ^]^ with a subsequent anisotropic coating,^[^ ^101^ ^]^ see Figure 7d. Varying substrate materials and changing irradiation strategies it is possible to tune a corrugated template aspect ratio, which influences the resulting magnetic properties of the deposited layer stack and allows to construct of tilted bit patterned media.^[^ ^101^ ^]^ To the same group of approaches should also be referred the usage of anodized aluminum oxide templates with conformal coating of magnetic materials.^[^ ^257^, ^258^ ^]^ This provides the fabrication of densely packed nanowire arrays, that could be utilized as magnetic memory devices,^[^ ^257^, ^258^, ^259^ ^]^ investigations of magnetic ratchets^[^ ^260^ ^]^ and investigation of the current‐induced motion of Bloch‐Point type domain walls.^[^ ^261^, ^262^ ^]^ These templates could be also used for the creation of multilayer core–shell nanostructures^[^ ^263^, ^264^ ^]^ and nanotubes^[^ ^265^ ^]^ arrays by means of atomic layer deposition. Another method for the fabrication of magnetic nanotubes is achieved by means of the specific material growing conditions of GaAs nanowires.^[^ ^266^, ^267^ ^]^ The conformal coating of these wires with ferromagnetic materials allows to prepare the core–shell ferromagnetic nanowires with a hexagonal cross section, see Figure 7e, whose magnetic states were studied recently.^[^ ^239^, ^268^ ^]^ Free‐standing curvilinear magnetic structures could be also fabricated using single intense femtosecond laser pulses through a glass substrate coated with a magnetic layer, which leads to the controlled thermo‐mechanical spallation and formation of nanocavities, nanomembranes or randomly shaped magnetic flakes depending on the pulse energies.^[^ ^269^ ^]^

The curvilinear magnetic nanostructures could be also formed by means of specific growing method, for example, the glancing angle deposition (GLAD), which employs oblique angle deposition and substrate motion.^[^ ^270^, ^271^, ^272^, ^273^ ^]^ Depending on the deposition rate and rotation speed of the substrate it is possible to fabricate 3D nanostructures with different morphologies (e.g., straight, inclined and bend pillars with variable diameter, zig‐zag, and fractal‐like structures), due to the competitive aggregation process of deposited atom accumulation on the substrate.^[^ ^273^ ^]^ By selecting GLAD parameters for ferromagnetic materials, it is possible to achieve the growing conditions for a large‐scale fabrication of periodic nanohelix arrays,^[^ ^46^, ^47^, ^240^, ^274^ ^]^ see Figure 7f. Due to their size and curvilinear nature they exhibit magneto‐chiral dichroism of light,^[^ ^274^ ^]^ which directly demonstrates coupling between their structural chirality and magnetism.^[^ ^240^ ^]^ The nonreciprocal shape of such magnetic nanohelices allows to create unique magnetic nanopropellers,^[^ ^46^ ^]^ by transferring these particles into a liquid, which is relevant for noninvasive biomedical applications for biopsies and targeted drug delivery^[^ ^47^, ^275^ ^]^ and enhanced optical properties of chiral magnetic nanohelices via magneto‐chiral dichroism (MCD).^[^ ^274^ ^]^

Recent progress in material science enabled the appearance of various microscale additive manufacturing technologies that provide the ability to fabricate 3D curvilinear geometries that are not limited by the conventional lithographic or specific layer growth techniques.^[^ ^276^ ^]^ One of the most prominent techniques of additive manufacturing of magnetic materials is focused electron‐beam induced deposition (FEBID)^[^ ^139^, ^141^, ^277^ ^]^ which in the recent years has reached a high level of maturity for the fabrication of complex‐shaped 3D nanoarchitectures.^[^ ^142^, ^144^, ^244^, ^278^, ^279^ ^]^ FEBID is based on the electron beam‐induced dissociation of a single or multiple precursor gas molecules, which results in a nonvolatile leftover shaped in accordance with the desired geometry. For instance, various ferromagnetic FEBID nanoarchitectures have already been demonstrated, such as nanovolcanoes,^[^ ^280^ ^]^ free‐standing nanostripes,^[^ ^241^ ^]^ see Figure 7g, nanoscale double‐helix structures,^[^ ^244^ ^]^ nanocubes and nanotrees,^[^ ^141^, ^142^, ^281^ ^]^ nanoamphoras,^[^ ^243^ ^]^ buckyballs,^[^ ^142^, ^243^ ^]^ and complex‐shape nanoarchitectures,^[^ ^282^ ^]^ see Figure 7g. The use of double precursor gases allows one to exploit the FEBID technique for material segregation, resulting in a one‐step fabrication of magnetic tubular structures with a nonmagnetic core at the scale of 100 nm.^[^ ^283^ ^]^ The two‐photon lithography is another additive technology that allows to create sub‐micrometer‐size self‐standing curvilinear objects of various shapes that could be covered with magnetic materials,^[^ ^140^, ^144^, ^245^, ^284^ ^]^ see Figure 7h. This enables “writing” of almost arbitrary geometries and magnetic materials, which leads to the rapid prototyping of complex magnetic curvilinear geometries.

#### 3.1.4 Characterization Methods

**Figure 8:**
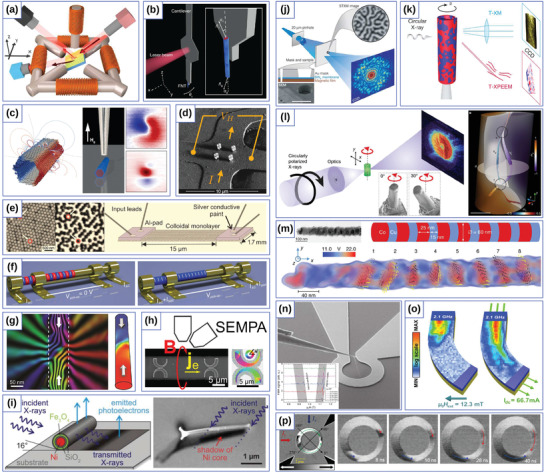
Advanced characterization techniques for curvilinear nanomagnets. a) Schematics of dark‐field MOKE. b) Dynamic cantiliver‐based magnetometry tip with magnetic nanotube at the end. c) Nano‐SQUID tip and reconstructed stray field of hexagonal magnetic nanotube. d) Micro‐Hall cross setup with nanocubes. e) Sketch of AMR measurements on self‐assembled magnetic nanoparticles. f) Schematics of an integrated GMI sensor operation. g) Electron holography of a domain wall in Ni nanocylinder. h) Sketch of SEMPA setup and color‐coded image from flat curvilinear structures. i) XMCD‐PEEM technique for 3D magnets with shadow contrast reconstruction. j) X‐ray spectro‐holography. k) Concept of MXT based on a combination of STXM and XMCD‐PEEM techniques for the 3D magnets. l) Hard X‐ray magnetic tomography setup based on ptychographic scans of a sample. m) Holographic vector field electron tomography of 3D nanomagnets. n) FMR measurements of a magnetic nanowire in Ω‐shaped resonator. o) BLS intensity distribution of spin waves in a curved stripe. p) Asymmetric ferromagnetic ring and STXM snapshots of automotive domain wall motion. a) Reproduced with permission.^[^ ^241^ ^]^ Copyright 2017, American Chemical Society. b) Reproduced with permission.^[^ ^285^ ^]^ Copyright 2018, American Physical Society. c) Reproduced with permission.^[^ ^286^ ^]^ Copyright 2018, American Chemical Society. d) Reproduced under the terms of the CC‐BY Creative Commons Attribution 4.0 International license (https://creativecommons.org/licenses/by/4.0).^[^ ^281^ ^]^ Copyright 2018, The Authors, published by MDPI. e) Reproduced with permission.^[^ ^254^ ^]^ Copyright 2010, AIP Publishing. f) Reproduced with permission.^[^ ^52^ ^]^ Copyright 2015, The Authors, published by Wiley‐VCH. g) Reproduced with permission.^[^ ^287^ ^]^ Copyright 2013, American Chemical Society. h) Reproduced with permission.^[^ ^288^ ^]^ Copyright 2020, American Chemical Society. i) Reproduced with permission.^[^ ^264^ ^]^ Copyright 2011, American Physical Society. j) Reproduced with permission.^[^ ^289^ ^]^ Copyright 2004, Springer Nature. k) Reproduced under the terms of the CC‐BY Creative Commons Attribution 4.0 International license (https://creativecommons.org/licenses/by/4.0).^[^ ^290^ ^]^ Copyright 2015, The Authors, published by Springer Nature. l) Reproduced with permission.^[^ ^291^ ^]^ Copyright 2017, Springer Nature. m) Reproduced under the terms of the CC‐BY Creative Commons Attribution 4.0 International license (https://creativecommons.org/licenses/by/4.0).^[^ ^292^ ^]^ Copyright 2015, The Authors, published by Wiley‐VCH. n) Reproduced under the terms of the CC‐BY Creative Commons Attribution 4.0 International license (https://creativecommons.org/licenses/by/4.0).^[^ ^293^ ^]^ Copyright 2019, The Authors. Published by Wiley‐VCH. o) Reproduced with permission.^[^ ^225^ ^]^ Copyright 2012, AIP Publishing. p) Reproduced with permission.^[^ ^18^ ^]^ Copyright 2017, American Physical Society.

The characterization of magnetic responses from curved structures utilizes the following methods:

**(i)** Magnetometry techniques that rely on measurements of magnetic responses from the curvilinear structures. These techniques could be divided for the simplicity into two groups:

**(i‐a)** Integral magnetometry techniques provide the magnetic responses over the large‐scale area and include the standard superconducting quantum interference device (SQUID) magnetometry, vibrating sample magnetometry (VSM) and magneto‐optic Kerr effect (MOKE) magnetometry,^[^ ^27^, ^144^, ^235^, ^248^, ^294^ ^]^ that recently was improved for high‐precision analysis of curvilinear structures by utilizing dark‐field MOKE magnetometry,^[^ ^241^ ^]^ see **Figure** **8**a.

**(i‐b)** Local magnetometry of the individual object quantitatively retrieve both the spatial distribution of the stray fields and magnetic properties. This group includes dynamic cantilever‐based^[^ ^267^, ^285^, ^295^, ^296^ ^]^ (Figure 8b) and nano‐SQUID magnetometries^[^ ^286^ ^]^ (Figure 8c). Here it should be referred the scanning reconfigurable magnetic force microscopy (MFM),^[^ ^245^, ^297^, ^298^ ^]^ that allows to quantify the 3D magnetization pattern of a magnetic nanoobject from a 2D data,^[^ ^299^ ^]^ and micro‐Hall magnetometry that was applied for magnetic characterization of complex‐shapes FEBID nanostructures^[^ ^142^, ^281^ ^]^ (Figure 8d).

**(ii)** Transport techniques, that provide the insight into the charge and spin transport properties of magnetic materials. These techniques include the group of magnetosensitive transport‐based electrical measurements, that employs: anisotropic magnetoresistance (AMR) effect for nanotubes,^[^ ^239^, ^300^ ^]^ monolayer of magnetic spherical caps^[^ ^254^ ^]^ (Figure 8e), ferromagnetic microhelices^[^ ^35^ ^]^ and nanomembranes;^[^ ^49^, ^301^, ^302^ ^]^ giant magnetoresistive (GMR) effect for nanomembranes;^[^ ^236^ ^]^ giant magnetoimpedance (GMI) effect^[^ ^52^ ^]^ Figure 8f; current‐induced spin torques.^[^ ^261^ ^]^

**(iii)** Magneto‐sensitive visualization techniques, that enable a direct reconstruction of magnetization textures of curvilinear objects. Valuable information can be obtained from the conventional “top‐view” imaging techniques, which provides access to complex magnetization patterns on curved magnetic systems. These techniques include scanning‐probe methods, for example, MFM, that provide a spatial resolution up to 50 nm for curved nanoobjects;^[^ ^9^, ^101^, ^169^, ^245^, ^247^, ^303^, ^304^, ^305^, ^306^, ^307^ ^]^ MOKE microscopy,^[^ ^27^, ^144^, ^235^, ^248^, ^294^ ^]^ that recently was enabled for pump‐probe time‐resolved measurements of micro‐tetrapod geometries;^[^ ^284^ ^]^ electron‐based methods, including Lorentz transmission electron microscopy (TEM)^[^ ^308^, ^309^ ^]^ and off‐axis electron holography,^[^ ^310^, ^311^ ^]^ that allows to perform the detail static reconstruction of magnetic textures in flat^[^ ^312^, ^313^ ^]^ and 3D^[^ ^240^, ^287^, ^314^ ^]^ curved geometries, see Figure 8g; scanning electron technique with polarization analysis (SEMPA),^[^ ^315^ ^]^ that is suitable for imaging flat curved magnetic geometries,^[^ ^288^, ^316^ ^]^ see Figure 8h; X‐ray‐based visualization methods, including X‐ray magnetic circular dichroism photoelectron emission microscopy (XMCD‐PEEM).^[^ ^9^, ^24^, ^25^, ^317^, ^318^ ^]^

While these methods are limited to the analysis of simple magnetic geometries, the visualization of complex 3D shape geometries requires the utilization of the tomographic‐based approaches. Thus, MOKE microscopy,^[^ ^248^ ^]^ that recently was enabled for tomography‐like screening of bulk samples;^[^ ^319^ ^]^ XMCD‐PEEM was successfully extended to image interior magnetization textures of complex curved magnetic nanoobjects by using transmission shadow contrasts,^[^ ^26^, ^181^, ^235^, ^261^, ^262^, ^264^, ^268^, ^320^, ^321^, ^322^ ^]^ see Figure 8i. X‐ray spectro‐holography^[^ ^289^ ^]^ could be also utilizes for the characterization of 3D curved magnetic nanostructures (Figure 8j), for example, magnetically capped nanospheres.^[^ ^323^ ^]^ Full field X‐ray microscopy, that combines magnetic transmission soft X‐ray microscopy (MXTM) with XMCD‐PEEM, was successfully applied to realize the concept of soft X‐ray magnetic tomography (MXT),^[^ ^290^ ^]^ see Figure 8k. Complementary, hard X‐ray magnetic tomography with ptychographic scans potentially allows for 10 nm resolution of 3D magnetic textures,^[^ ^291^ ^]^ see Figure 8l. The electron holography is extended to holographic vector field electron tomography,^[^ ^292^, ^324^ ^]^ see Figure 8m, that allows to reconstruct 3D magnetic textures with a sub 10 nm resolution.

**(iii)** Dynamic magnetization methods, that rely on time domain for magnetic characterization. These techniques include the ferromagnetic resonance (FMR) methods,^[^ ^325^ ^]^ whose sensitivity for the study of curved magnetic objects could be substantially improved by utilizing microresanator loops,^[^ ^293^ ^]^ see Figure 8n. Another valuable dynamic measurement method is Brillouin light spectroscopy (BLS),^[^ ^326^ ^]^ that recently was improved by using high‐sensitive BLS microscopy,^[^ ^225^, ^226^, ^327^ ^]^ see Figure 8o. Also, it should be mentioned the possibility to perform dynamic magnetic imaging using scanning transmission X‐ray microscope (STXM),^[^ ^119^, ^239^, ^318^, ^328^, ^329^, ^330^, ^331^ ^]^ see Figure 8p.

### 3.2 Perspectives of Curvilinear Ferromagnets

The main perspective of curvilinear magnetism is to address experimentally the available theoretical predictions and develop a fundamental understanding of curvature‐induced anisotropic and chiral magnetic responses. Despite numerous experimental trials in fabrication and characterization, magnetic responses from the 3D magnetic architectures and their interpretations are complex and ambiguous, while the key theoretical predictions^[^ ^31^, ^33^, ^38^, ^39^, ^161^, ^173^, ^332^ ^]^ remain not yet confirmed experimentally. To address these shortcomings, the incorporation of all mentioned theoretical and experimental approaches is necessary to bridge the gap between fundamental and applied approaches to the study of curvilinear ferromagnetism. Within this incorporation framework recently it was made the first experimental step to observe and quantify the influence of one of curvilinear parameters (local curvature) on magnetic responses.^[^ ^24^, ^25^ ^]^ Using the simplest curvilinear geometry with well‐defined shape of flat parabolic stripe and simple magnetic textures (e.g., transversal domain wall) along with theoretical predictions allowed to validate existence of chiral curvature‐induced DMI in flat curvilinear structures made of a conventional 10 nm thick Permalloy.^[^ ^24^ ^]^

#### 3.2.1 Theory

Despite the first attempt to build the comprehensive micromagnetic theory of curvilinear ferromagnetic shells by combining local and nonlocal interactions,^[^ ^61^ ^]^ the final theoretical framework still requires an extensive research to quantitatively reveal the predictions for the specific geometries and magnetic textures. The following topics should be brought in focus:

(i) Curvilinear magneto‐mechanical interaction should be addressed in the generalized theory of curvilinear magnetism. Due to a strong interconnection between the geometry and symmetry of the magnetic subsystem, the magnetostriction and magnetoelastic interactions^[^ ^99^, ^333^, ^334^ ^]^ are envisioned to be crucial for the curvilinear magnetic framework as they are defining additional binding interconnections between the magnetic and geometrical spaces. Namely, the magnetostriction interaction through even a small tensile deformation could change the equilibrium magnetization distribution in torsional spring nanowires.^[^ ^335^ ^]^ Contrary, a magnetization change could mediate the shape transformation of a flexible magnetic object,^[^ ^336^, ^337^ ^]^ for example, the transition from vortex to the onion state in a ferromagnetic elastic ring will cause elliptic‐shape deformations.
(ii) Curvilinear spintronics and spinorbitronics is yet to be developed. Due to the fact the geometrically broken symmetry in curvilinear magnetic systems plays a similar role as the SOC in multilayer systems, we envision the emergence of novel topologically related and curvature‐induced transport effects in both real and reciprocal spaces. Namely, the propagation of electron through a smoothly varying magnetization distribution give rise to the topological Hall effect.^[^ ^165^, ^338^ ^]^ Also, in the case of helical nanowires it is predicted the strong influence of the curvature and torsion on the motion of magnetic domain walls through the modification of anti‐damping and field‐like torques.^[^ ^34^, ^339^ ^]^
(iii) Thermodynamics of curvilinear magnetic systems: Thermodynamics properties of magnetic systems are crucial for fundamental studies of low‐dimensional magnets.^[^ ^340^ ^]^ The extension of thermodynamic approaches to the curvilinear magnetic systems is envisioned to be decisive for the determination of equilibrium magnetic states and curvature‐induced effects in complex‐shaped magnetic systems. Thus, the temperature stability of magnetic states pinned at curvature gradients, as well as, statistical distribution of magnon eigenstates in curved bodies should be addressed by expanding existing thermodynamic approaches on curved ferromagnets.
(iv) Curvilinear magnonics: In this field the spin degree of freedom is exploited to transport, detect and manipulate information using spin‐waves or magnons. This field requires a comprehensive micromagnetic theory.^[^ ^61^ ^]^ It has been shown experimentally that the propagation of spin waves through curved waveguides requires the connection between the spin‐wave propagation direction and the local curvilinear frame of reference.^[^ ^225^ ^]^ Moreover, the recent experimental and theoretical studies show that the nonlinear dynamics of spin waves, such as the three magnon scattering might allow for the usage of spin waves for reservoir computing. Thus, it is becoming important to extend the micromagnetic continuum theory with the quantum mechanical rate equation to study the multiple magnon scattering and temperature effects. The investigation of magnon–phonon interactions that possesses new degree of control for the spin‐wave modes is also a field for perspectives.

#### 3.2.2 Numerics

The development of micromagnetic simulations for the curvilinear magnetism is strongly correlated with the subsequent evolution of the FEM‐based packages. The individual approach for the development of various group‐related private micromagnetic packages seems to be time and resources inefficient due the lack of generalized and multifaceted development of these projects. In the inspiration from the FDM‐based micromagnetic open‐source projects like OOMMF and mumax3, that are now by default are standard computational tools for flat magnetic systems, the development of a powerful open‐source FEM‐based micromagnetic package is envisioned to be the first main milestone, that should be addressed. The following inclusion to this code of all magnetic interactions and effects is the next milestone, which is important not only for the curvilinear magnetism community, but also for the whole micromagnetic community. Namely, a self‐consistent implementation of the magneto‐mechanical interactions, the spatial dependent micromagnetic parameters, the extension of the spin‐transfer and spin‐orbit torques into curvilinear geometries are examples still not available in simulation codes. Moreover, the speed up of the micromagnetic simulations should not anymore be done with hardware accelerations, as most of the micromagnetic codes are already GPU‐accelerated, but rather must utilize novel numerical methods, which would be different from the time integration of the LLG equation of motion. For instance, the linear dynamics, such as the calculation of the spin‐wave modes could be drastically accelerated by using eigensolver‐based dynamic matrix approaches, where the LLG equation is linearized around a stable equilibrium configuration.^[^ ^341^ ^]^ Thus, it can directly result in the dispersion relation (eigenvalues) and the mode profiles (eigenvectors). In the following, such an approach in combination with the quantum mechanical rate equations could allow to numerically study nonlinear dynamics, for example, magnon–magnon scattering to high orders, including temperature effects. Also the full‐scale micromagnetic simulations that rely on LLG equation can only be feasible if they utilize highly efficient approaches for magnetostatic field calculations, which is the most time‐consuming part of any micromagnetic simulation. Thus, it should be mentioned the Fredkin–Koehler^[^ ^342^ ^]^ hybrid finite element/boundary element and recently developed $H^{2}$‐matrix compression methods^[^ ^343^ ^]^ and others,^[^ ^344^ ^]^ that allowed to dramatically speed up FEM‐based simulations.

#### 3.2.3 Fabrication

The evolution of the available methods and application of novel interdisciplinary approaches are the two main perspectives for the further development of magnetic fabrication methods. This will allow to achieve sub‐nanometer high‐resolution quality in all dimensions, which is necessary for the needs of curvilinear magnetism to separate effects due to the curvature from spurious defects like pinning of magnetic textures on inhomogeneities. Thus, it is necessary to perform the development across the following lengthscales:

(i) *Atomic Scale*: This milestone could be achieved by the use of dedicated bottom‐up approaches and novel materials. The low dimensional flat curvilinear magnetic structures can be fabricated by means of scanning tunneling microscopy (STM).^[^ ^345^, ^346^, ^347^, ^348^ ^]^ A tip of the STM can be used to position magnetic atoms one by one and thus to form curved chains of desirable atoms, for example, quantum corrals made of Fe atoms.^[^ ^349^ ^]^ Due to the extremely precise single‐atom manipulation this method has a low throughput of sample production, which may be used for fabrication of very specific flat curved nanowire and curved ribbons geometries. It should be noted here the possibility to use the 2D van der Waals ferromagnetic materials,^[^ ^350^ ^]^ for example, single‐layer CrI_3_^[^ ^351^, ^352^ ^]^ to realize curvilinear geometries.
(ii) *Nanoscale*: Magnetic nanowires can be fabricated by the pyrolysis of ferrocene, which produces iron‐filled carbon nanotubes.^[^ ^353^, ^354^ ^]^ Since this method of fabrication also leads to the formation of different curved bundles of nanowires, this makes them ideal to study different curvature‐ and torsion‐induced effects, as well as, magnetization dynamics in the linear and nonlinear regime. Recently it was shown,^[^ ^293^ ^]^ that pyrolysis allows to fabricate a high‐quality single‐phase (body‐centered cubic) crystal structure, that represents the overcoming of the quality problem, which was typical for magnetic nanowire fabrication. A further technique, up to now mostly applied to fabricate round nanowires of topological insulators is based on ion bombardment of a polymer material with ions at relativistic speeds.^[^ ^355^ ^]^ The resulting round holes of about 20–30 μm in length and several dozens of nanometer in diameter can be filled with magnetic material using chemical deposition to produce high‐quality core–shell nanotubes. Another perspective method for the fabrication of curvilinear nanowires is the DNA‐origami^[^ ^356^, ^357^, ^358^ ^]^ functionalized with magnetic nanostructures. The advantage of this method is the self‐assembly of DNA molecules, which results in a straightforward process where the designed long single‐stranded molecule is folding into the desirable shape. This process is rapid and potentially allows to fabricate scalable nanostructures.
(iii) *Mesoscale*: The established 2D lithographic methods, that allow to fabricate high quality magnetic nanostructures with nm‐scale resolution, is promising to extend in the 3D by using spatially corrugated curvilinear templates. Namely, it is possible to form the random textures by incident ion‐beam erosion of substrates^[^ ^101^, ^255^, ^256^ ^]^ or periodical textures by means particles self‐assembly.^[^ ^45^, ^169^, ^246^, ^247^ ^]^ Varying these approaches for the template fabrication will help to form 3D templates with predefined topographic and altitude profiles, which is the required developments that would presumably lead to the fabrication of 3D nanostructures with the same quality and precision as those obtained within the 2D lithography techniques. The utilization of the strain‐induced magnetic origami^[^ ^359^, ^360^, ^361^ ^]^ is another perspective direction, that allows to construct various predefined 3D magnetic designs in combination with standard lithography. The mesoscale magnetic fabrication methods also include direct writing methods:
  (iii‐a) Direct 2D writing through the irradiation of B2‐A2 phase alloys (e.g., FeAl alloy^[^ ^362^ ^]^) allows to reach the element spatial resolution at the same or better level than lithography‐based approaches. This method is based on thin films that are paramagnetic after preparation (B2 phase), but obtain ferromagnetic order after the irradiation due to the induced chemical disorder (A2 phase). As shown recently,^[^ ^313^ ^]^ using He^+^ or Ne^+^ irradiation in He‐ion microscope it is possible to write into FeAl flat curvilinear magnetic nanostructures with a 2 nm precision.
  (iii‐b) Direct 3D writing by means of FEBID allows to fabricate sub‐micrometer 3D structure with a nm resolution. This method allows both the fabrication of corrugated or curvilinear nonmagnetic templates, that can be covered using sputtering or electro‐deposition for core–shell nanostructures, and the direct writing of magnetic objects with arbitrary shape. The current limitations are the available precursor gasses to be used and thus the magnetic materials which can be fabricated. There is still room for improvement regarding the homogeneity and quality of the fabricated nano‐objects. However with subsequent heat treatment for Co and Fe samples significant quality improvements have already been shown.^[^ ^141^, ^363^, ^364^, ^365^ ^]^ In the case of fabrication scalable micrometer‐size curvilinear structure arrays, the two‐photon lithography technique combined with electrodeposition could be utilized.^[^ ^140^, ^284^ ^]^

#### 3.2.4 Characterization

##### Characterization Methods

The main perspectives here are lying in the extension of standard flat characterization methods into the 3D and the subsequent evolution of the available curvilinear methods in the direction of the resolution improvements in both time and spatial domain. Within this it should be mentioned the following perspective directions:

(i) *Static Characterization Methods*: Here it should be mentioned that such high‐resolution magneto‐sensitive microscopic methods like spin‐polarized STM (SP‐STM)^[^ ^345^, ^346^, ^347^, ^348^ ^]^ or nitrogen‐vacancy (NV) center microscopy^[^ ^366^, ^367^, ^368^, ^369^ ^]^ have not yet been applied to the field of curvilinear magnetism. Namely, the SP‐STM allows to make the atomic scale resolution scans of flat curved magnetic structures, while NV center microscopy allows to make static and dynamic 3D imaging of stray fields. The application of this methods will not only provide the detailed picture of magnetic textures in curvilinear magnets, but could also reveal novel magnetochiral effects at nanoscale. The further development of the magneto‐sensitive 3D visualization techniques, including soft and hard X‐rays, electron and neutron^[^ ^370^, ^371^ ^]^ tomographies, lays in the efficient software reconstruction and modifications of the sample environment at beamlines to enable the high‐quality tomographic scans at reasonable time. Also, it is expected that in the case of magnetic visualization of micrometer‐scale curved nanoobjects the conventional magneto‐optic‐based approaches will become applied more often.
(ii) *Dynamic Characterization Methods*: The main perspective here is the extension of tomographic‐based magnetic visualization methods in the time domain, in order to perform the 3D study of magnetization dynamics. The standard pump–probe approach could be used in the frame of available tomographic methods. This will require the subsequent evolution of measurement methodology from both hardware and software sides. X‐ray free‐electron laser opens another possibility to perform ultrafast dynamic studies with high spatial resolution by utilizing methods of X‐ray holography. Another possibility to obtain dynamic measurements of the magnetic systems is the development of ultrafast detectors. For instance, recently it was shown experimentally the possibility to perform dynamic magnetic imaging of flat curved geometries by means of scanning electron microscope with polarization analysis (SEMPA),^[^ ^288^ ^]^ that has a time resolution of less than 2 ns.^[^ ^315^ ^]^
(iii) *Transport Methods*: In touch with the prospective development of spintronics and spin‐orbitronics in flat magnetic systems, it is necessary to extend the transport‐based methods on curved magnetic architectures. Thus, the possibility to measure the field‐like and damping‐like spin‐orbit torques from the first and second harmonics of Hall signal^[^ ^372^ ^]^ provides a quantitative assessment for the intrinsic DMI constant,^[^ ^373^ ^]^ which could be adapted to the curvilinear magnetic systems and extrinsic or mesoscale DMI assessment. Moreover, the development of curvilinear spintronics and spin‐orbitronics will allow to incorporate the curvilinear effects into already known concepts of magnetic memory and logic devices.

##### Applications

On the application side, the key milestone is to realize device prototypes based on numerous exciting theoretical predictions of curvilinear magnetism. In this respect we could define the following perspective application realizations:

(i) *Data Storage and Logic Applications*: The studies of noncollinear magnetic structures such as domain walls, vorticies and skyrmions have attracted much attention because of the potential applications in future magnetic devices such as the race‐track memory^[^ ^210^, ^211^, ^219^ ^]^ and magnetic logic devices.^[^ ^14^, ^220^, ^221^, ^222^ ^]^ The robust domain walls in nanotubes with velocities exceeding the spin wave phase velocity are promising candidates for race‐track memory devices.^[^ ^177^, ^179^, ^374^, ^375^, ^376^, ^377^, ^378^ ^]^ Further nonvolatile memory devices could be realized using topologically protected states: skyrmions on magnetic spherical shells^[^ ^33^ ^]^ or curvilinear bumps^[^ ^38^, ^39^, ^379^ ^]^ and domain walls in Möbius rings.^[^ ^31^ ^]^ The curvature‐induced effects define the appearance of specific topological magnetic states in nanotori^[^ ^153^, ^182^, ^183^, ^380^ ^]^ and nanotubes^[^ ^381^, ^382^, ^383^ ^]^ as well as influence skyrmion propagation along magnetic nanotubes^[^ ^384^ ^]^ and curved racetracks.^[^ ^385^ ^]^ The curved geometries could be functional parts of artificial magnetoelectric media, which is based on curvilinear helimagnets sandwiched between two piezoelectric layers.^[^ ^335^ ^]^ The electric field induces tiny change of geometrical parameters, that cause a transition between homogeneous and periodic magnetic states and features the large converse magneto‐electric effect.^[^ ^335^ ^]^ Curvilinear magnetic geometries also provide the perspective possibility to control ultracold atoms through the dynamic reconfiguration of stray fields by means of magnetic texture change.^[^ ^59^, ^60^ ^]^ Thus, this will allow to develop quantum information processing application with a dynamic functionalization.
(ii) *Magnonic Applications*: The development in the field of magnonics has made huge efforts in the last decade to realize concepts to use spin waves for data processing. Many concepts have been proposed theoretically and experimentally, leading to prototype building blocks of spin‐wave‐based logic.^[^ ^129^, ^130^, ^386^, ^387^, ^388^ ^]^ Such effects as interference,^[^ ^128^, ^389^, ^390^ ^]^ self‐focusing,^[^ ^391^ ^]^ nonlinear damping,^[^ ^390^ ^]^ scattering on artificial defects,^[^ ^392^ ^]^ refraction,^[^ ^393^ ^]^ lenses,^[^ ^394^ ^]^ fibers,^[^ ^395^ ^]^ diffraction,^[^ ^396^ ^]^ modification of the spin wave wavelengths by magnetic fields^[^ ^397^, ^398^, ^399^ ^]^ and tunable microwave filters based on spin‐wave propagation in magnetic microtubes^[^ ^231^, ^232^ ^]^ were extensively studied. However, achieving a magnonic technology platform enabling wave‐based computing requires a drastic change of multilevel magnonic building blocks that can be achieved using 3D nano‐objects. This by default will require the combination of flat and curved magnonic waveguides. The magnetochiral effects provide a magnonic toolbox for the manipulation of spin waves, however at first the detailed understanding of it on the spin‐wave transport is necessary. Moreover, the already discussed effects (in flat geometries) such as the lensing, diffraction, or refraction has also be re‐evaluated in the presence of surface curvature and topology. We believe, that exploring the 3D nano‐objects key devices of a future magnon based computer can be realized in the near future, such as diodes, or repeaters, which would heavily rely on the nonreciprocal effects induced by the curvature. These devices enable an energy‐efficient multilevel magnonic technology platform and the path toward magnon computing.
(iii) *Curved Magnetic Field Sensors*: There are intensive application‐oriented activities on the use of mechanically compliant magnetic field sensors. This research field is known as shapeable magnetoelectronics^[^ ^40^, ^41^, ^400^ ^]^ and includes flexible,^[^ ^51^, ^401^, ^402^, ^403^, ^404^, ^405^, ^406^, ^407^, ^408^, ^409^, ^410^, ^411^, ^412^, ^413^, ^414^, ^415^ ^]^ printable,^[^ ^416^, ^417^, ^418^, ^419^, ^420^ ^]^ stretchable,^[^ ^409^, ^420^, ^421^, ^422^ ^]^ and mechanically imperceptible^[^ ^42^, ^43^, ^44^, ^409^, ^415^, ^422^, ^423^ ^]^ magnetosensitive elements. Thus, the magnetoelectronics is find itself in the following applications: tracking displacement and rotation of magnetic field in conventional machinery;^[^ ^409^, ^411^, ^424^, ^425^ ^]^ orientation in space;^[^ ^42^, ^43^ ^]^ accurate control of actuation;^[^ ^44^, ^409^, ^415^ ^]^ motion tracking and touchless human–machine interaction.^[^ ^42^, ^43^, ^44^, ^400^, ^420^ ^]^ Despite these numerous applications and relatively high technology level, commercial shapeable magnetoelectronic products are absent.
(iv) *Biomedical Applications*: Until recently, biomedical magnetic applications were limited by their empirical experimental studies of geometrically induced effects related only to the shape asymmetry and chirality of magnetic objects. Namely, the nature of helical magnetic objects defines their unidirectional motion in the external magnetic field. This enables their application for the targeted drug^[^ ^46^, ^47^, ^275^ ^]^ and sperm delivery for artificial fertilization.^[^ ^48^, ^53^, ^54^, ^55^, ^56^, ^57^, ^58^ ^]^ Also, asymmetric magnetic Janus particles could be also used for the drug delivery,^[^ ^45^, ^48^ ^]^ while the curved magnetic objects could be utilized for microfluidic applications.^[^ ^49^, ^50^, ^51^, ^52^ ^]^ With the emerging field of curvilinear magnetism, it is possible to establish design rules that allow to explicitly predict magnetic effects and textures for the specific curvilinear geometries. This unique toolbox opens broad opportunities for the utilization of curvilinear micro‐ and nanomagnetism for novel biomedical applications. For instance, spherical Janus particles with a 100‐nm‐thick Permalloy cap enable the stabilization of a topologically stable magnetic vortex state, which was used to induce guided thermophoresis motion of particles via simultaneous usage of AC and DC magnetic fields.^[^ ^253^ ^]^ Together with recent advances in nanofabrication it becomes possible to create various nanomachines with different magnetic and mechanical properties for specific biomedical applications. Still, this novel field requires subsequent investigation of its potential for nanoscale biomedical applications.

## 4 Curvilinear Antiferromagnets

Compared to the rather well established topic of curvilinear ferromagnetism, the field of curvilinear antiferromagnetism represents a terra incognita in establishing the role of geometry on the magnetic responses of geometrically curved antiferromagnetic wires and shells.

### 4.1 State of the Art

#### 4.1.1 Theory and Computations

Antiferromagnets represent a reach class of technologically promising materials^[^ ^426^, ^427^, ^428^, ^429^, ^430^, ^431^ ^]^ with a specific internal magnetic structure. The key difference between ferro‐ and antiferromagnets is the sign of the exchange integral between the neighboring spins. Depending on the structure of the lattice unit cell, this leads to drastic changes of static and dynamic magnetic responses and predetermines a variety of magnetic configurations including different types of domain walls, spin spirals, and skyrmions (see for review^[^ ^427^, ^432^, ^433^, ^434^, ^435^ ^]^ and references therein).

The state‐of‐the‐art model of a general antiferromagnet utilizes the Heisenberg Hamiltonian with the spin operators $\hat{S}$ localized at the lattice sites^[^ ^426^, ^436^ ^]^

(31) $$\hat{H}=\sum_{ij}J_{ij}{\hat{S}}_{i}\;\cdot \;{\hat{S}}_{j}$$

Here, the sum running over the nearest neighbors and J_*ij*_ is the exchange integral between *i*th and *j*th sites. The exchange interaction itself may not lead to an antiferromagnetic ordering at zero temperature and manifests subtle differences between quantum and semi‐classical approaches of low‐dimensional systems.^[^ ^426^, ^432^, ^436^ ^]^ If applicable, the latter description treats spins as classical vectors placed in the given spatial positions. In the simplest case, the Hamiltonian for the classical magnetic moments $\mu_{i}$ reads^[^ ^104^ ^]^

(32) $$\begin{array}{l} \frac{H}{S^{2}}=\underset{ij}{\sum }{\text{J}}_{ij}\mu_{i}\;\cdot \;\mu_{j}+\underset{i}{\sum }{\text{K}}_{i}{\left(\mu_{i}\;\cdot \;e_{\text{A}}\right)}^{2}\\ +\;2\mu_{\text{B}}^{2}\underset{i\neq j}{\sum }\left[\frac{\mu_{i}\cdot \mu_{j}}{r_{ij}^{3}}-3\frac{\left(\mu_{i}\;\cdot \;r_{ij}\right)\left(\mu_{j}\;\cdot \;r_{ij}\right)}{r_{ij}^{5}}\right] \end{array}$$

where *S* is the spin length and $\left|\mu_{i}\right|=1$. Here, the first term represents the Heisenberg exchange with the exchange integral *J*_*ij*_ measured in units of energy. The second term is the single‐ion anisotropy with the anisotropy coefficient K_*i*_ at *i*th site. The last term is the dipolar interaction with μ_B_ being the Bohr magneton and *g*‐factor assumed to be equal to two. Additional terms not written here may include intrinsic DMI, and other types of anisotropy.^[^ ^104^ ^]^ Note, that the expression in Equation (32) is valid also for curvilinear antiferromagnets.

A conventional way to describe antiferromagnets within a phenomenological approach is to introduce a set of coupled vector fields, combined into primary and driving vector order parameters.^[^ ^104^ ^]^ The simplest model of a bipartite antiferromagnet with sublattice magnetizations $m_{1}$ and $m_{2}$ (e.g., NiO, Mn_2_Au,^[^ ^437^ ^]^ Cr_2_O_3_, MnF_2_,^[^ ^438^ ^]^ Ba_2_CuGe_2_O_7_ or K_2_V_3_O_4_^[^ ^433^ ^]^) introduces a Néel vector (staggered magnetization) $n=(m_{1}-m_{2})/2$ and a ferromagnetic vector $m=(m_{1}+m_{2})/2$, which can be considered to be small compared to n for many practically relevant cases. Some materials (e.g., triangular antiferromagnet Mn_3_Ir^[^ ^439^ ^]^) require several order parameters each possessing a sizable vector length. The director nature of the order parameter (vector ***n*** is physically equivalent to ‐***n*** according to its definition above) leads to a higher symmetry of ground states and new types of antiferromagnetic domain walls in comparison with ferromagnets.^[^ ^440^ ^]^ We note, that rigorous definition of an antiferromagnet implies the presence of crystallographic symmetry, permuting the sublattices, but not a compensated total magnetization of the sample, see discussion in ref. ^[^ ^436^ ^]^ and ref. ^[^ ^441^ ^]^.

The statics and dynamics of magnetic textures in two‐sublattice bulk antiferromagnets can be described using the following Lagrangian^[^ ^426^, ^432^ ^]^

(33) $$L=\int \bar{L}dr=-\frac{2M_{s}}{\gamma_{0}}\int m\;\cdot \;\left[n\times \frac{\partial n}{\partial t}\right]dr-E$$

where *M*_s_ is the saturation magnetization of each sublattice, *E* is the phenomenological energy functional corresponding to the spin Hamiltonian Equation (32) and integration is performed over the sample's volume. In the case of $\left|m\right|\ll \left|n\right|$, the ferromagnetic vector can be eliminated, which leads to the Lorenz‐invariant equation of motion for the Néel vector in uniaxial materials in absence of external fields.^[^ ^442^, ^443^ ^]^

**Figure 9:**
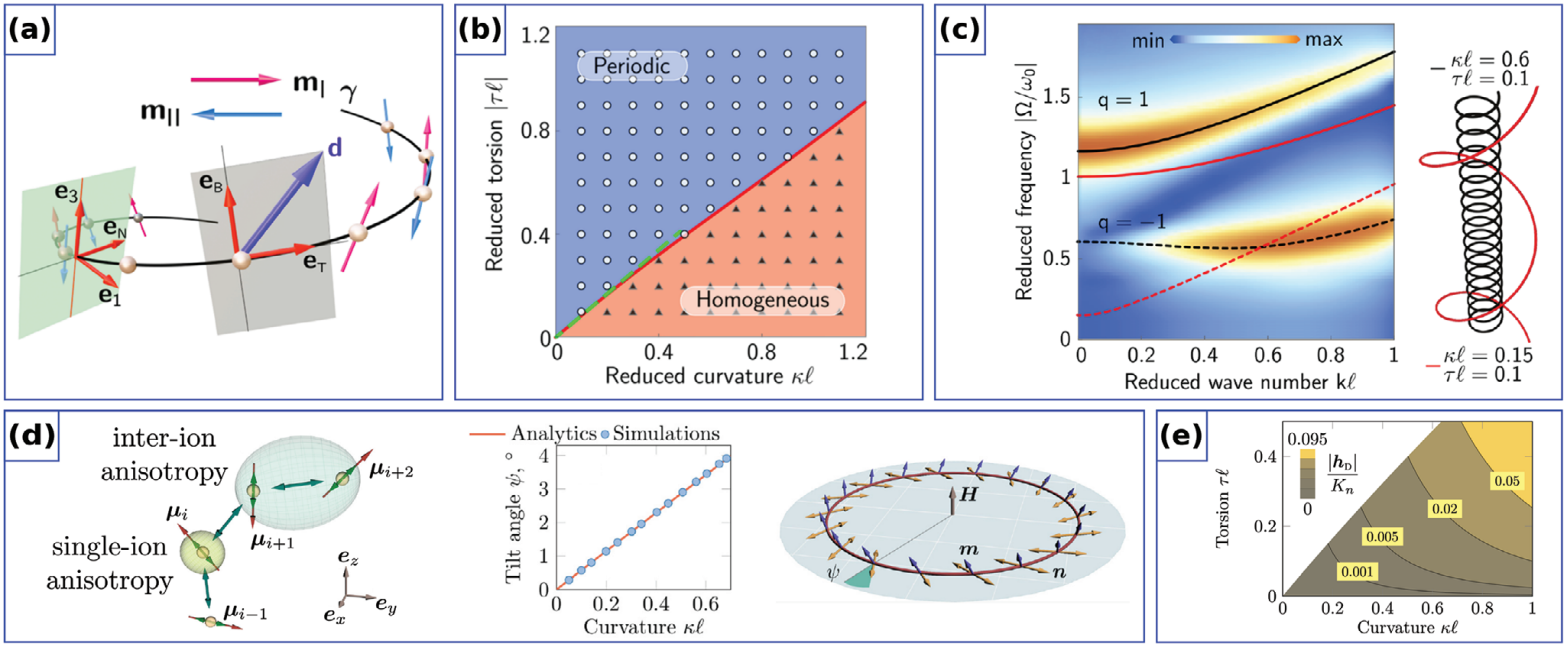
Statics and dynamics of curvilinear antiferromagnetic spin chains. a) Magnetic response of a curvilinear spin chain γ with the antiferromagnetic exchange and dipolar coupling. Two sublattices $m_{\text{I,II}}$ are represented by magenta and light‐blue arrows. Vector d of the curvature‐induced DMI is represented by dark‐blue arrow. The effective hard and easy axes are labeled by $e_{1}$ and $e_{3}$, respectively. b) Diagram of ground state of helix spin chain. The solid red (dashed green) line shows the helimagnetic phase transition between homogeneous and periodic ground states obtained by spin‐lattice simulations (analytically). c) Example of spin wave dispersion in reduced units (left) for two antiferromagnetic helical chains (right). Here, ω_0_ is the frequency of the antiferromagnetic resonance and ℓ is the magnetic length. The lower branch possesses a region with the negative group velocity for sufficiently large curvature κ and torsion τ. a–c) Reproduced with permission.^[^ ^444^ ^]^ Copyright 2020, American Chemical Society. d) Schematics of the single‐ and inter‐ion anisotropies in spin chain: the red and green arrows correspond to magnetic moments $\mu_{i}$ and anisotropy axes, respectively (left), curvature‐driven tilt angle ψ of anisotropy (center), and antiferromagnetic ring in spin‐flop state (right). e) Strength of the curvature‐induced homogeneous DMI with $h_{D}=D_{0}(n_{N}e_{T}+n_{T}e_{N})$ and *K*_*n*_ being the anisotropy constant. d,e) Reproduced under the terms of the CC‐BY Creative Commons Attribution 4.0 International license (https://creativecommons.org/licenses/by/4.0).^[^ ^445^ ^]^ Copyright 2021, The Authors, published by AIP Publishing.

We are interested in the influence of the geometry (i.e., curvature and torsion for quasi‐1D systems, or principal curvatures for thin shells) on magnetic textures in curvilinear antiferromagnets. There are first steps in the understanding of curvilinear antiferromagnets, especially for spin chains.^[^ ^444^, ^446^ ^]^ It can be rigorously shown that in the absence of intrinsic anisotropy, K_*i*_ = 0, the dipolar coupling between spins in Equation (32) leads to the effective hard axis anisotropy with the axis along the tangential direction.^[^ ^444^ ^]^ This allows to present the linear density of Larganian Equation (33) for the intrinsically achiral and isotropic 1D spin chain in the long‐wave approximation as^[^ ^444^ ^]^

(34) $$\begin{array}{l} \bar{L}=\frac{M_{s}^{2}}{\gamma_{0}\Lambda }{\left(\frac{\partial n}{\partial t}\right)}^{2}-[A{n^{\prime }}_{\alpha }{n^{\prime }}_{\alpha }+AF_{\alpha \beta }(n_{\alpha }{n^{\prime }}_{\beta }-{n^{\prime }}_{\alpha }n_{\beta })\\ +\;AK_{\alpha \beta }n_{\alpha }n_{\beta }+K^{\text{eff}}n_{T}^{2}],\;\;\alpha ,\beta =T,N,B \end{array}$$

where Λ is the constant of the uniform exchange and $K^{\text{eff}}\approx 10.8\mu_{B}^{2}S^{2}/a^{4}$ with *a* being the lattice constant is the coefficient of the effective hard‐axis anisotropy. The curvature and torsion determine tensors *F*_*αβ*_ (2) and *K*_*αβ*_ (15). They lead to the appearance of the geometry‐driven anisotropy and inhomogeneous DMI, see **Figure** **9**a. The second term in square brackets in Equation (34) determines the geometry‐driven DMI and can be presented by an alternative expression $E_{\text{DMI}}^{\text{afm}}=\varepsilon_{\alpha \beta \gamma }d_{\alpha }n_{\beta }{n^{\prime }}_{\gamma }$ with *ε_αβγ_* being the Levi–Civita symbol and the vector $d=2A(\tau e_{T}+\kappa e_{B})$ acting as the Dzyaloshinskii vector, confer Equations (16) and (18). This form of DMI is allowed in crystals with *C*_*n*_ and *S*_4_ symmetry groups for 1D textures. Since d is linear in curvature and torsion, one can expect strong chiral effects of curvature in these systems. In contrast to ferromagnets, the dipolar interaction locally defines the easy plane for the Néel vector and the direction of n within this plane is determined by the geometry‐driven anisotropy *K*_*αβ*_ ∝ κ^2^, τ^2^ only.^[^ ^444^ ^]^

In the case of spin chains arranged along flat curves, this gives the antiferromagnetic ground state in the direction perpendicular to the chain plane determined by the geometry‐driven easy axis anisotropy.^[^ ^444^ ^]^ The latter can also contribute to the intrinsic anisotropy of spin‐orbit origin,^[^ ^446^ ^]^ observed, for example, in Cr wheels.^[^ ^447^, ^448^ ^]^ The interplay between the hard axis of dipolar‐induced anisotropy with the extrinsic easy axis and chiral interaction stemming from the exchange interaction due to curvature renders intrinsically isotropic achiral spin chains as biaxial chiral helimagnets, see Figure 9b. The emerging curvature‐induced Dzyaloshinskii–Moriya interaction is geometrically tunable and is responsible for the helimagnetic phase transition.^[^ ^444^ ^]^

The local magnetization in spin chains along straight lines can appear as a dynamical effect, either when the system is exposed to the external magnetic field ***H*** or due to the presence of noncollinear spin textures.^[^ ^449^, ^450^ ^]^ For example, for the case of a uniaxial spin chain with the easy axis anisotropy with a coefficient *K*_ea_, it reads

(35) $$m\approx -\frac{a_{0}}{2l}n\times \left[\omega_{0}\frac{\partial n}{\partial t}+\left(lm\prime -\frac{H}{H_{0}}\right)\right]$$

where *a*_0_ is the lattice constant, $l=\sqrt{A/\left|K_{\text{ea}}\right|}$, $H_{0}=\sqrt{\Lambda \left|K_{\text{ea}}\right|}/M_{s}$ is the spin‐flop field and ω_0_ = γ_0_ *H*_0_ is the frequency of the antiferromagnetic resonance. Here, the pre‐factor determines the ratio between the effective fields of the anisotropy and exchange. The expression (35) is valid for curvilinear spin chains as well, however the meaning of *H*_0_ and ω_0_ can be changed to some characteristic quantities. The spatial derivative n′ in Equation (35) originates from the exchange and, in addition to ${n^{\prime }}_{\alpha }$, α = T, N, B, is determined by κ and τ. This leads to the weak ferromagnetic response for any spin chain with $n\neq e_{B}$.^[^ ^445^ ^]^

The common models of the anisotropy in antiferromagnets are the single‐ion and inter‐ion anisotropies, see Figure 9d. The first one originates from the spin‐orbit interaction in systems with *S* ≥ 1, see the second sum in Equation (32). The Hamiltonian of the inter‐ion anisotropy involves pairs of neighboring spins similarly to the second term in the dipolar sum in Equation (32) (see also the discussion of magnetostatics in Section 3.1). It can be determined by the anisotropic exchange, spin‐orbit interaction and includes the local part of the dipolar interaction.^[^ ^104^, ^436^ ^]^ The tracking of the geometry by the anisotropy axis and two magnetic sublattices in the antiferromagnetic spin chain leads to the family of curvature‐induced effects, stemming from the anisotropy itself, which is different from the case of ferromagnets. In both Hamiltonians of the single‐ and inter‐ion anisotropy, appears an additional secondary anisotropy axis, which can determine the direction of n in a spin‐flop phase, see Figure 9d. The single‐ion anisotropy leads to the additional nonchiral DMI of the homogeneous type, $E_{\text{DM}}^{\text{hom}}=D_{0}(n_{N}m_{T}+n_{T}m_{N})$ with *D*_0_ = *κK* *S* ^2^/2 and *K* = *K*_*i*_ for all magnetic sites, see Figure 9e.^[^ ^445^ ^]^

**Figure 10:**
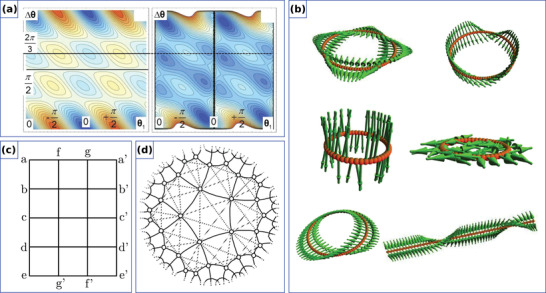
Thermodynamics and statistical physics at spin lattices with nontrivial topology. a) Internal (left, *E*) and Gibbs free (right, *G*) energies of the ring consisting of four sites. θ_1_ and Δθ are the angle of the first spin and deviation of between the neighboring spins. b) Equilibrium configuration in antiferromagnetic spin chain shaped as a planar ring: a knot soliton due to geometrical frustration and Bloch wall (top), easy‐axis and easy‐plain excitations (center), antiferromagnetic state shown in ring and in a chain with open ends for clarity. c) Schematics of boundary conditions for the lattice with topology of Klein bottle. Pairs of sites marked as • and •′ are connected. d) Hyperbolic lattice drawn in the Poincaré disk. The nearest‐neighbor and next‐nearest neighbor exchange bonds between magnetic sites are shown by black and dashed lines, respectively. a,b) Reproduced with permission.^[^ ^446^ ^]^ Copyright 2017, American Physical Society. d) Reproduced with permission.^[^ ^451^ ^]^ Copyright 2008, American Physical Society.

Closed spin chains described by the Hamiltonian (Equation (32)) with $e_{A}=e_{B}$ along the binormal direction exhibit a variety of long‐living metastable states stabilized by the boundary conditions as well as interplay between the exchange, anisotropy, and dipolar interaction.^[^ ^446^ ^]^ The number of spins in the ring brings about an additional degree of freedom for the magnetic sublattice. While even number of spins support the pure antiferromagnetic ordering,^[^ ^444^, ^446^ ^]^ the additional, odd magnetic moment breaks the antiferromagnetic state and leads to an appearance of the spiral ordering. **Figure** **10**a represents an example of the energy landscape for a chain consisting of four spins with a strong single‐ion anisotropy and antiferromagnetic exchange. The internal energy *E* in Equation (33) and the free energy *G* reads^[^ ^446^ ^]^

(36) $$G=E-k_{B}T\log\Gamma_{E}$$

with *k*_B_ being the Boltzmann constant, *T* temperature, and Γ_*E*_ the number of states with the energy *E* are shown in Figure 10a, left and 10a, right. The angle θ_1_ corresponds to the deviation of the first spin from $e_{B}$ and Δθ characterizes the phase shift of the each next spin. Nonzero Δθ leads to the spin spiral state with three configurations Δθ = π/4, π/2 and 3π/4 represented by the low‐energy saddle points. Additionally, there are twelve local energy minima (depicted by blue regions). Dark‐ and light‐blue regions correspond to the collinear and noncollinear metastable states, respectively. The stability of the latter ones is determined by the strength of the anisotropy and can be enhanced by the effective anisotropy from the dipolar coupling between spins. Taking into account entropy, splitting of the observed states appears, leading to other possible noncollinear states.^[^ ^446^ ^]^

Depending on the ring size, Möbius‐like and kink textures appear to satisfy the boundary conditions.^[^ ^446^ ^]^ The free energy landscape of rings exhibits a variety of long‐living metastable states stabilized by the boundary conditions as well as the interplay between the exchange, anisotropy and dipolar interactions,^[^ ^446^ ^]^ see Figure 10b. Spin spirals represent a reach family of lowest excited states. Depending on the easy‐ or hard‐axis type of anisotropy, complex noncollinear states are possible. They include multi‐domain textures and even the Möbius state in the ring with even number of spins.^[^ ^446^ ^]^

Curvature results in the drastic modification of dynamic responses of curvilinear antiferromagnets, see Figure 9c. In the particular case of helical spin chains in the homogeneous state, the dispersion law reads^[^ ^444^ ^]^

(37) $$\Omega^{2}=c^{2}k^{2}+\omega_{0}^{2}(K_{0}+K_{3})+\omega_{0}^{2}\frac{q}{2}\sqrt{{\left(K_{0}-K_{3}\right)}^{2}+4D_{3}^{2}k^{2}l^{2}},\;\;q=\pm 1$$

where *c* is the characteristic magnon speed and ω_0_ is the frequency of antiferromagnetic resonance. In the limit of small curvatures and torsions, analytical expressions for the coefficients are *K*_0_ ≈ 1 + *ℓ* ^2^(κ^2^ − τ^2^), *K*_3_ ≈ κ^2^ *ℓ* ^2^ and *D*_3_ ≈ 2*κℓ*. The finite curvature turns the lower dispersion branch from acoustic to the low‐frequency optical (*q* = −1) one introducing a gap $\Omega_{\text{gap}}^{\text{low}}\approx c\kappa$. In spin chains with large enough curvature and torsion, this branch possesses regions with negative group velocity in the dispersion curve,^[^ ^444^ ^]^ which makes these objects potentially promising to study correlated and long‐living magnon states, also for magnon condensates.^[^ ^452^, ^453^, ^454^, ^455^, ^456^, ^457^, ^458^ ^]^

In addition to micromagnetics, the community already addressed thermodynamics of curvilinear antiferromagnets within Ising models at specific graphs, see Figure 10c,d. The topology of connection between Ising spins determines critical behavior of the lattice. In particular, the partition function for Potts antiferromagnets of cylindrical, toroidal and Möbius boundary conditions is calculated.^[^ ^459^, ^460^ ^]^ The hyperbolic geometry of spin space leads to specific phases in frustrated lattices.^[^ ^451^ ^]^ On the numerical side, the Monte Carlo techniques are applied to describe critical phenomena and thermodynamic properties of curvilinear geometries like tubes and Möbius rings^[^ ^460^, ^461^, ^462^, ^463^, ^464^ ^]^

Numerical tools used for the investigation of planar antiferromagnetic samples are directly applied to address the curvilinear ones.^[^ ^444^, ^465^ ^]^ Typically, spin‐lattice simulators are used, which allow to take into account a rapid change of the direction of spins on the atomic level. Historically, their development started from Monte Carlo simulations.^[^ ^191^, ^192^, ^446^, ^466^, ^467^, ^468^ ^]^ At present, available tools involve a direct solution of LLG equations for spin vectors in time domain^[^ ^96^, ^189^, ^191^, ^192^, ^193^, ^194^, ^469^, ^470^, ^471^, ^472^, ^473^ ^]^ and the stochastic LLG modeling.^[^ ^474^, ^475^, ^476^ ^]^ This allows to address dynamic and thermodynamic effects in samples with a complex lattice structure,^[^ ^477^ ^]^ core–shell nanoparticles^[^ ^478^ ^]^ and finite size phenomena.^[^ ^477^, ^479^ ^]^ The speed‐up of the calculations due to a high number of variables is achieved using GPU.^[^ ^189^, ^191^, ^192^, ^193^, ^194^ ^]^

#### 4.1.2 Experimental Studies

**Figure 11:**
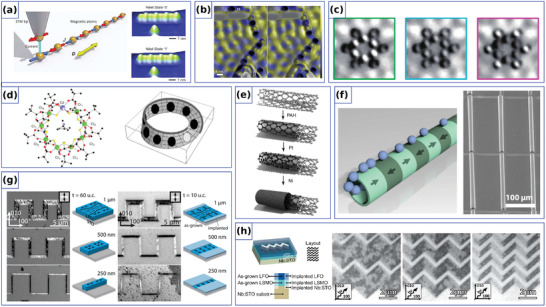
Experimental realization of flat and 3D curvilinear antiferromagnetic samples. a) Schematics of antiferromagnetically coupled array of atoms with the SP‐STM tip (left), and two states of Fe atoms on Cu substrate. b) Atomic logic gate with gates “α” and “β”, which changes the state of the sixth atom from “1” to “0”. c) Artificial kagomé lattice of antiferromagnetically coupled Fe atoms at fields −1 T (left), 0 T (center) and +1 T (right). d) Molecular wheels: Cr_8_Cd (left), and frustrated Cr_7_Ni planar ring supporting a magnetic Möbius state (right). e) Schematics of synthesis of Ni and NiO shells on carbon nanotubes. f) Schematics of a transport of super‐paramagnetic microbeads though magnetic channel (left) and SEM image of exchange biased rolled‐up tubes (right). g) X‐PEEM images of free‐standing and embedded square wave shaped nanostripes of LaFeO_3_ with the corresponding magnetic textures. h) Schematics and XMLD‐PEEM imaging of embedded zig‐zag LaFeO_3_ stripes with the regular domain pattern. a) Reproduced with permission.^[^ ^480^ ^]^ Copyright 2012, AAAS. b) Reproduced with permission.^[^ ^481^ ^]^ Copyright 2011, AAAS. c) Reproduced with permission.^[^ ^482^ ^]^ Copyright 2012, Springer Nature. d‐left) Reproduced under the terms of the CC‐BY Creative Commons Attribution 4.0 International license (https://creativecommons.org/licenses/by/4.0).^[^ ^448^ ^]^ Copyright 2015, The Authors, published by Springer Nature. d‐right) Reproduced with permission.^[^ ^483^ ^]^ Copyright 2004, Wiley‐VCH. e) Reproduced with permission.^[^ ^484^ ^]^ Copyright 2007, Wiley‐VCH. f) Reproduced with permission.^[^ ^294^ ^]^ Copyright 2016, American Chemical Society. g) Reproduced with permission.^[^ ^20^ ^]^ Copyright 2019, AIP Publishing. h) Reproduced with permission.^[^ ^19^ ^]^ Copyright 2011, American Physical Society.

Even in the absence of a systematic theoretical description of the influence of the geometry on curvilinear antiferromagnets, these systems are already accessible experimentally. STM and SP‐STM are versatile techniques to assemble and visualize atom‐by‐atom designed structures, which represent artificial quantum spin chains arranged along flat curves. The antiferromagnetic ordering can be stabilized by the strong enough anisotropy. Namely, chains of Fe atoms possess in‐plane^[^ ^480^ ^]^ or out‐of‐plane^[^ ^481^, ^482^ ^]^ easy axis, see **Figure** **11**a–c. The inter‐atom exchange of the Ruderman–Kittel–Kasuya–Yosida nature allows to vary the coupling type by means of the inter‐atomic distance and build antiferromagnetic chains as well as frustrated ones.

Antiferromagnetically coupled spin chains are intensively studied within the topic of organic magnets^[^ ^485^, ^486^, ^487^ ^]^ where the exchange coupling constants can vary in a wide range.^[^ ^488^ ^]^ Molecular rings indirectly involve geometry into the observed properties via the number of spins and periodic boundary conditions. For example, Cr_*x*_ molecular magnets (also studied by DFT techniques^[^ ^489^, ^490^, ^491^ ^]^) behave in a different way being open and closed into wheels.^[^ ^448^ ^]^ The change between odd and even number of magnetic ions (V_7_, Cr_7_ or Cr_8_Cd arranged into wheels) allows to address geometrically frustrated system with the Möbius topology of the magnetic state,^[^ ^446^, ^448^, ^483^, ^492^ ^]^ see Figure 11d. Copper‐based organic antiferromagnetic compounds can be of helix shape with a large curvature and torsion with respect to the distance between atoms.^[^ ^493^, ^494^ ^]^

Examples of 2D antiferromagnetic shells are represented by cylindrical surfaces, like tubes with a single antiferromagnetic layer or coupled ferro‐ and antiferromagnetic bilayers. Such samples are created, for example, using carbon cylindrical matrices (NiO, see Figure 11e),^[^ ^484^ ^]^ porous alumina templates (core–shell Co/CoO)^[^ ^495^, ^496^ ^]^ or being grown individually (arrays of multiferroic BiFeO_3_ tubes).^[^ ^497^ ^]^ The latter exhibits a weak ferromagnetism due to the break of the spin spiral in a confined geometry.^[^ ^497^ ^]^ Imprinting of the magnetic texture from the ferromagnetic core and antiferromagnetic shell explains measured hysteresis loops in nanowires.^[^ ^496^ ^]^ Tubular architectures of larger dimensions are created by strain engineering techniques,^[^ ^227^, ^228^, ^359^, ^498^ ^]^ which allow to obtain rolled‐up tubes with ferromagnet/antiferromagnet bilayers^[^ ^294^ ^]^ with the highest quality films, see Figure 11f.

Although experimentally available, the responses of these curvilinear antiferromagnets are not analyzed from the positions of curvilinear magnetism. For instance, curvature‐induced chiral effects and anisotropy are still to be reported and quantified.

Effects of the sample boundaries on magnetic textures represent a special case of interplay between the geometry and the magnetic order parameter. In ferromagnets, the significant contribution of the magnetostatics in the energy functional is versatile, leading to the stabilization of specific textures, for example, vortices in nanodots^[^ ^12^ ^]^ and influencing the dynamics of magnetic topological solitons by the topography of the sample boundaries.^[^ ^15^, ^18^, ^499^, ^500^ ^]^ In antiferromagnets, the shape of the boundaries can alter the order parameter via magnetoelastic coupling^[^ ^501^ ^]^ and surface anisotropy.^[^ ^502^ ^]^ This anisotropy of the shape‐like behavior can play a significant role in specific materials as it was reported for Lanthanum oxides, where the magnetoelastic interaction contributes to the domain configuration.^[^ ^21^, ^503^ ^]^

Epitaxially grown perovskite LaFeO_3_ thin films possess the in‐plane easy axes determined by the substrate: SrTiO_3_ imprints its 〈100〉 axis into overlying layer,^[^ ^503^ ^]^ while La_0.7_Sr_0.3_MnO_3_ leads to the appearance of two easy axes 〈110〉 and 〈100〉 in pseudocubic notation.^[^ ^503^ ^]^ This allows to create planar curvilinear ribbons and observe a highly regular domain pattern in zig‐zag geometries, free standing or drawn by a local disruption of the structural and magnetic ordering by Ar^+^ ion implantation, see Figure 11g,h.^[^ ^19^, ^20^ ^]^ The domains are arranged with respect to the ribbon period. The orientation of the Néel vector is determined by the surrounding. In the case of free‐standing narrow enough structures, n is aligned along the boundary (“easy‐tangential” like anisotropy). In contrast, embedded ribbons possess the “easy‐normal” like anisotropy with the orientation of n perpendicular to the ribbon within the film plane. The effect of boundary is confirmed by analysis of wide ribbons: the boundary ordering of the Néel vector is the same as for narrow stripes, while the internal area possesses a random domain distribution. Similar effects are reported for Lanthanum‐based oxides islands of different shape imprinted into nonmagnetic matrix.^[^ ^21^ ^]^

Effects stemming from curvilinear geometries in ferromagnets are often accompanied by the boundary effects, which are active even in planar structures. It is important to develop the respective understanding of the boundary effects also for antiferromagnets to be able to distinguish them from the curvilinear effects and to utilize for applications. As it was recently shown for α‐Fe_2_O_3_, antiferromagnetic domain walls can effectively reflect spin waves and act as boundaries of dynamically reconfigurable curvilinear magnonic waveguides.^[^ ^504^ ^]^ The longer propagation length of magnons is reported for $\left(1\bar{1}02\right)$ films below the Morin temperature, which support large domains and translational domain walls.^[^ ^504^ ^]^ The relation between the pinning potential *E*_pin_ and the domain wall energy *E*_dw_ determines the maximal wave vector, which can be still reflected^[^ ^504^ ^]^

(38) $$q_{\text{crit}}=4\frac{E_{\text{pin}}}{E_{\text{dw}}}\frac{1}{\Delta }$$

with Δ being the domain wall width.

The boundary conditions for the Néel vector n in a chiral antiferromagnet read^[^ ^505^ ^]^

(39) $$n\times \left[2A(\nu \;\cdot \;\nabla )n-D_{1}\nu \times n\right]=0$$

for the DMI energy of the bulk type $E_{\text{DM1}}^{\text{afm}}=D_{1}\int n\;\cdot \;\nabla \times ndr$ with ν being the surface normal, and^[^ ^505^, ^506^ ^]^

(40) $$n\times \left\{2A(\nu \;\cdot \;\nabla )n+D_{2}\left[n_{z}\nu -(\nu \;\cdot \;n)e_{z}\right]\right\}=0$$

for the DMI of the interfacial type $E_{\text{DM2}}^{\text{afm}}=D_{2}\int [n_{z}(\nabla \;\cdot \;n)-(n\;\cdot \;\nabla )n_{z}]dr$, where *D*_2_ is the DMI constant and $e_{z}$ direction is along the film normal. Note, that in the presence of the homogeneous DMI of the chiral type, $E_{\text{DM3}}=2d_{3}\int e_{z}\cdot (m\times n)dr$ with the coefficient *d*_3_, the boundary conditions should take into account the ferromagnetic vector $m=(d_{3}/\Lambda )e_{z}\times n$.^[^ ^506^ ^]^ In thin films with *d*_3_ ≠ 0 and *D*_2_ ≠ 0, this leads to the boundary magnetization.^[^ ^506^ ^]^ In confined samples with *D*_1_ ≠ 0, such antiferromagnetic textures, like domain walls between phase domains and skyrmions, possess sizeable shape distortions of up to five magnetic lengths in depth, which determine the minimal sample size to have bulk‐like properties.^[^ ^505^ ^]^

**Figure 12:**
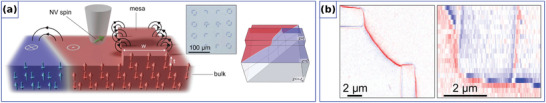
Domain and surface topography acting on linear and nonlinear excitations. a) Schematics of Cr_2_O_3_ single crystal with nontrivial surface topography imaged by NV microscopy (left), and schematic of 3D domain wall plane under the mesa (right). b) Gradual transition of the domain walls between the mesa boundaries due to the pinning at the topographic landscape. a,b) Reproduced with permission.^[^ ^22^ ^]^ Copyright 2021, The Authors, published by Springer Nature.

The specific example of interplay between the geometry and antiferromagnetic texture illustrating the role of Equation (39) is characteristic for the samples with patterned surface, see **Figure** **12**a. This can be effectively combined with the nitrogen vacancy microscopy for imaging individual domain walls in Cr_2_O_3_^[^ ^22^ ^]^ as well as noncollinear textures in BiFeO_3_.^[^ ^507^ ^]^ A magnetoelectric collinear antiferromagnet Cr_2_O_3_ supports translational domain walls, which have a high mobility in bulk single crystals. Very recently it is shown a possibility to move them by laser dragging and pin them at the surface by litographically patterned rectangular mesas of width *w* and thickness *t*.^[^ ^22^ ^]^ The domain wall crossing a mesa experiences a distortion, which can be explained by the exchange‐driven Neumann boundary conditions for the Néel vector (39) with *D*_1_ = 0. This forces the domain wall plane within the mesa to be perpendicular to its sides and results in the bend of the domain wall plane under the mesa at the characteristic length of about 0.34*w*, see Figure 12a. The domain wall shape within the mesa can be described by the ansatz^[^ ^22^ ^]^

(41) $$y(x,z)=\frac{4k}{w}\sum_{i=0}^{\infty }\frac{{\left(-1\right)}^{i}}{\lambda_{i}^{2}}\mathrm{sech}\lambda_{i}t\cosh\lambda_{i}(t-z)\sin\lambda_{i}x$$

with λ_*i*_ = (1 + 2*i*)π/*w* and line *y*(*x*, 0) = *k*_0_ *x* determining the orientation of the domain wall just below the mesa.

The geometrically driven domain wall pinning at edges of mesas is determined by the energy gain, related to the change of the domain wall's area during dragging. The nontrivial topography of the sample's surface creates an artificial map of pinning centers via the boundary conditions (39). This leads to the formation of curvilinear states of the domain walls at equilibrium,^[^ ^22^ ^]^ see Figure 12b. The latter ones are of technological interest. A possibility to determine the domain wall position, for example, by electrical gates to switch it from one side of the mesa to another one opens perspectives to utilize the surface magnetization at the mesa as a single bit of information in a low‐energy consuming random access memory.^[^ ^22^ ^]^

### 4.2 Perspectives of Curvilinear Antiferromagnets

The further development of the field of curvilinear antiferromagnetism should involve both theory and experiment to address the impact of geometry on responses of curved antiferromagnets. In addition, in a number of materials antiferromagnetism coexists with superconductivity so that both, ferromagnetic^[^ ^508^, ^509^, ^510^, ^511^, ^512^, ^513^, ^514^, ^515^ ^]^ and antiferromagnetic^[^ ^516^, ^517^, ^518^, ^519^, ^520^, ^521^, ^522^, ^523^ ^]^ materials can host curvature effects on magnetism and superconductivity (see also Section 5) at the same time.

#### 4.2.1 Theory

In ferromagnets, the link between the geometry and magnetic texture is created by the anisotropy^[^ ^23^, ^101^, ^209^ ^]^ and/or magnetostatics, following the shape of wires^[^ ^524^, ^525^ ^]^ or shells.^[^ ^107^, ^108^, ^526^ ^]^ In bulk antiferromagnets^[^ ^104^ ^]^ and curvilinear spin chains,^[^ ^444^ ^]^ the dipolar interaction acts as anisotropy. Its role in more complex curvilinear lattices (including other sources of anisotropy specific to antiferromagnets) is yet to be understood which requires relevant energy terms to be included in the theory of curvilinear antiferromagnetism. At the same time, antiferromagnets might offer other ways of linkage of the geometry and magnetic subsystem. For instance, magnetoelasticity, which is expected to be strong in antiferromagnets, where the mechanical strain is one of the mechanisms leading to the domains formation,^[^ ^527^, ^528^, ^529^ ^]^ for example, in NiO samples.^[^ ^530^ ^]^

*Quantum Systems*: Even for spin chains, current theories^[^ ^444^, ^446^ ^]^ treat them classically. Still, as effectively 1D systems immersed into 3D space, they exhibit specific quantum properties^[^ ^531^ ^]^ and may not have a long‐range magnetic order.^[^ ^486^ ^]^ The effects of curvature in quantum systems are studied as a result of boundary conditions^[^ ^479^, ^532^, ^533^ ^]^ and in problems of electron transport in confined geometries.^[^ ^5^, ^534^, ^535^ ^]^ Recently the spin transport^[^ ^536^ ^]^ and quantum spin dynamics in a curved space was described.^[^ ^537^, ^538^ ^]^ These activities continue a step toward curvilinear spintronics and spin‐orbitronics in both, ferro‐ and antiferromagnetic systems, while influence of the geometry on magnetization statics in quantum chains is still to be understood.

*Spin Chains*: The applicability of the approach, based on the dynamics of spin vectors in chains^[^ ^444^ ^]^ is justified for the case of strong anisotropy^[^ ^432^ ^]^ which suppresses the impact of quantum effects. These classically treated systems represent a useful playground, possessing all main features of more complex systems, for example, shells, and should be developed further by addressing effects stemming from different sources of the intrinsic anisotropies (such as single‐ion or inter‐ion one in addition to the dipolar interaction) and the intrinsic Dzyaloshinskii–Moriya interaction. A significant influence of curvature and torsion on the magnetic responses is expected in particular on the spin‐flop transition^[^ ^436^ ^]^ and soliton dynamics driven by strong magnetic fields.^[^ ^32^, ^435^ ^]^

Curvilinear antiferromagnetic shells offer larger flexibility in manipulating sample responses compared to their ferromagnetic counterparts. The microscopic difference arises from the various arrangement of crystal lattices (A‐, C‐, and G‐type antiferromagnets). Within the micromagnetic formalism, the free energy functional contains similar terms as for ferromagnets, related to the isotropic exchange, chiral, and anisotropic interactions, applied magnetic fields as well as homogeneous^[^ ^104^ ^]^ and frustrated^[^ ^539^, ^540^, ^541^, ^542^, ^543^ ^]^ DMI. The analytical representation of the free energy functional is dependent on the lattice symmetry and temperature.^[^ ^104^, ^544^ ^]^ These interactions can be written in a curvilinear reference frame to access the effects, stemming from the geometry. To avoid spurious effects from the reference frame, it is crucial to build the theory using tangential derivatives as was recently done for ferromagnets.^[^ ^61^ ^]^ Qualitatively, in curvilinear antiferromagnetic shells one can expect additional extrinsic anisotropic and chiral terms entering the energy functional, whose strength will be proportional to the principal curvatures. The realistic models should also involve the break of antiferromagnetic ordering at the interfaces^[^ ^545^ ^]^ and provide a route to describe curvilinear exchange‐biased multilayers, where the additional intrinsic surface DMI can appear.^[^ ^430^ ^]^ Furthermore, the impact of lattice imperfections at the sample's boundary or in polycrystalline media resulting magnetically disordered phases^[^ ^426^, ^546^ ^]^ should be clarified.

**Figure 13:**
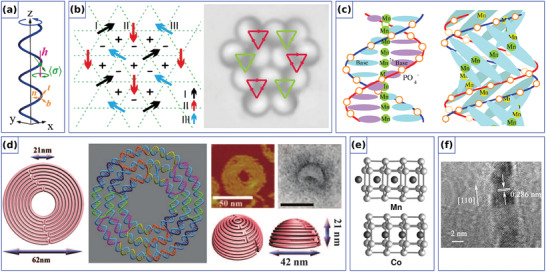
Perspectives of curvilinear antiferromagnetism. a) Evolution of the orientation of electron spin during motion along helix. Reproduced with permission.^[^ ^538^ ^]^ Copyright 2020, American Physical Society. b) Frustrated cluster of magnetic colloidal Janus particles (right) and arrangement of their magnetic moments (left). Reproduced with permission.^[^ ^249^ ^]^ Copyright 2008, American Physical Society. c) Mn‐DNA of B‐form (left) and A‐form (right). Reproduced with permission.^[^ ^570^ ^]^ Copyright 2007, The Physical Society of Japan. d) DNA origami arranged in a complex 3D shape: design, AFM image of nine‐layer concentric DNA ring and TEM image of the hemispherical cap. Reproduced with permission.^[^ ^356^ ^]^ Copyright 2011, AAAS. e) Doped silicon nanotubes in ferro‐ (Co) and antiferromagnetic (Mn) states. Reproduced with permission.^[^ ^571^ ^]^ Copyright 2003, American Physical Society. f) TEM image of a single crystal CoO wire with a diameter of 7 nm. Reproduced with permission.^[^ ^545^ ^]^ Copyright 2011, AIP Publishing.

Antiferromagnets are promising materials for spintronics and spin‐orbitronics devices due to staggered spin‐orbit torques^[^ ^547^, ^548^, ^549^, ^550^ ^]^ allowing an effective switching of the magnetic state in the absence of demagnetizing fields. While these torques are dependent on the lattice structure and symmetry, it is important to know, how the curvature effects will influence spin‐orbit torques. A motion of electrons confined in curvilinear geometries is followed by the geometry‐governed precession of the spin polarization,^[^ ^537^, ^538^ ^]^ see **Figure** **13**a. This may affect the symmetry of spin‐orbit torques and alter current‐driven dynamics of antiferromagnetic domain walls and skyrmions in curvilinear shells.

The peculiarity of magnetic systems, possessing antiferromagnetic coupling is the phenomenon of frustration, leading to the incommensurate textures as well as topological solitons.^[^ ^551^, ^552^ ^]^ In this respect, curvilinear analogues for the classical ANNI (axial next‐neighbor Ising) or Frenkel–Kontorova^[^ ^553^ ^]^ models and the corresponding mean‐field models should be developed. One can expect the modification of modulated phases^[^ ^544^, ^554^ ^]^ due to curvature. To study effects of frustration, soft matter model systems could be of value,^[^ ^249^, ^555^, ^556^ ^]^ see Figure 13b. For example, Janus particles as interacting macrospins can form clusters and chains of complex shape.^[^ ^557^, ^558^, ^559^, ^560^, ^561^, ^562^, ^563^ ^]^ An interesting aspect is that these systems allow to study effects due to the pure dipole–dipole interaction without exchange effects.

Scaling phenomena and critical behavior are extensively studied by Monte Carlo techniques with respect to system topology and curvature of the spin space for Ising models with ferromagnetic coupling.^[^ ^564^, ^565^, ^566^, ^567^, ^568^, ^569^ ^]^ The effects of antiferromagnetic exchange and frustration, related to the curvature are far less represented and require further understanding. These effects are dependent on the sign of the Gauss curvature $\tilde{K}$. This prediction is in contrast to ferromagnets, where the mean curvature $\tilde{H}$ determines the curvature effects in magnetostatics and principal curvatures govern chiral and anisotropic effects, stemming from the exchange.^[^ ^61^ ^]^

#### 4.2.2 Numerics

Following the discussion of ferromagnets, there is a need for a single platform for numerical studies of antiferromagnets. Again, an open source platform seems to be useful for further intensive development of the code which should include magnetoelastic interactions and a bending of crystal lattices. The progress in simulation tools for antiferromagnets should be also accompanied by the formulation of standard problems for their verification, similarly to the standard μMAG problems^[^ ^572^ ^]^ and their extension for chiral ferromagnets.^[^ ^573^ ^]^

Practically, at the moment there are only spin‐lattice simulations, which are extensively used to describe differently shaped curvilinear antiferromagnets. Their limitation is the number of interacting spins, corresponding to the nanometer‐size samples. Full scale micromagnetic FEM solvers for the antiferromagnetically coupled magnetization sublattices is the way to address this limitation and describe arbitrary sample shapes as it is already done for ferromagnets.^[^ ^186^, ^188^, ^201^, ^205^, ^206^, ^207^, ^343^, ^574^ ^]^ To the best of our knowledge, there is no micromagnetic simulation packages for antiferromagnets up to now. Still, there are intensive developments, for instance, in the group of Prof. Sinova at the Johannes Gutenberg University Mainz.^[^ ^575^ ^]^ The key difference with ferromagnetic solvers lies in the simultaneous solution of coupled LLG equations for each of the sublattices of the sample. The description of realistic samples will require an implementation of proper models of magnetoelasticity^[^ ^501^, ^576^, ^577^, ^578^ ^]^ and stray fields. The magnetoelastic coupling potentially can utilize numerical methods for the evaluation of magnetostatics in ferromagnets.^[^ ^343^ ^]^ The dipole–dipole interaction is important for comparison of simulations with imaging techniques utilizing stray fields, for example, NV microscopy^[^ ^22^, ^507^, ^579^, ^580^ ^]^ and for the description of the sublattice magnetization dynamics at the sample boundaries.^[^ ^506^ ^]^

In ferromagnetism, the combination of spin lattice and micromagnetic approaches, the so‐called multiscale techniques have established themselves as a promising tool to investigate dynamics of topological singularities.^[^ ^581^, ^582^, ^583^, ^584^ ^]^ This approach would be even more important for the numerical description of antiferromagnets because the micromagnetic approach alone cannot address effects, related to disclinations and dislocations in crystal lattices. These structural defects lead to the stabilization of specific magnetic solitons, which are absent in ferromagnets.^[^ ^435^ ^]^ The multiscale description would be a convenient method to take into account the dipolar interaction for inhomogeneous textures, mentioned above.

#### 4.2.3 Fabrication

The already applied methods for the fabrication of curvilinear antiferromagnets can be complemented by techniques chosen with respect to the desired scale and shape of the objects.

*Atomic Scale*: Planar yet geometrically curved 1D antiferromagnetically coupled chains can be created atom by atom using STM.^[^ ^349^, ^480^, ^481^, ^482^ ^]^ To realize 1D antiferromagnets ordered in planar or space curves, available techniques of the DNA origami could come of area of relevance.^[^ ^585^, ^586^, ^587^ ^]^ DNA molecules are flexible building blocks for complex nanostructures in 3D,^[^ ^356^, ^357^, ^358^, ^588^, ^589^ ^]^ see Figure 13d, and support an embedding of magnetically coupled Mn,^[^ ^570^, ^590^ ^]^ Cu^[^ ^591^, ^592^, ^593^ ^]^ or Fe^+3^ ions,^[^ ^593^ ^]^ see Figure 13c.

*Nanoscale*: Magnetically filled carbon nanotubes with embedded antiferromagnetically ordered structures^[^ ^571^, ^594^, ^595^ ^]^ possess a high aspect ratio and support planar bends as well as twists resulting in curvilinear flat or space quasi‐1D architectures, see Figure 13e. The stabilization of the long‐range antiferromagnetic order is possible in quasi‐1D structures: nanowires, which are grown from the antiferromagnetic compounds and core–shell structures, for example, NiO^[^ ^484^ ^]^ or CoO^[^ ^495^, ^496^, ^545^ ^]^ of different size, see Figure 13f. The latter also can be produced with antiferromagnetic core of 7 nm diameter with a ferromagnetic surface state.^[^ ^545^ ^]^

FEBID and focused ion beam induced deposition (FIBID) are already established as powerful techniques for the creation^[^ ^138^, ^139^ ^]^ of ferro‐ and antiferromagnetic materials in the same structure, as it was shown by fabrication of Co/CoF_3_.^[^ ^596^ ^]^ The fabrication of curvilinear antiferromagnets should be possible as well, although not yet demonstrated. For instance, FEBID/FIBID‐processed ferromagnetic structures can be oxidized after growing as it was shown for Ni by transformation into homogeneous NiO deposits.^[^ ^597^ ^]^ One should note that the structural defects can significantly alter magnetic states and should be also addressed theoretically.

*Mesoscale*: Thin curvilinear shells can be produced using high quality antiferromagnetic thin films deposition on modulated substrates. The modulation can be realized either by self assembled spherical particles as was already done for exchange biased [Co/Pt]/IrMn multilayers^[^ ^598^, ^599^, ^600^, ^601^ ^]^ or by strain engineering.^[^ ^23^ ^]^ For these studies, not only thin films, but also 2D van der Waals crystals (e.g., CrI_3_ with antiferromagnetically coupled layers,^[^ ^352^ ^]^ MnPS_3_ with a honeycomb lattice,^[^ ^602^, ^603^ ^]^ layered GdTe_3_^[^ ^604^ ^]^) can be used. They support a number of exotic phenomena and long‐range magnetic ordering. It is important, that these materials reveal the anisotropy axis perpendicular to layer planes,^[^ ^602^, ^605^, ^606^, ^607^ ^]^ which is the key aspect to “enable” curvilinear effects by coupling of geometry and magnetic system. In this respect of particular advantage could be strain engineering technique^[^ ^41^, ^238^, ^608^, ^609^, ^610^ ^]^ as it allows to process thin films in conventional planar geometries followed by 3D structures in the spirit of origami approaches.^[^ ^359^, ^361^ ^]^ In addition, the two‐photon lithography^[^ ^140^, ^144^ ^]^ should be validated for the realization of curvilinear antiferromagnets at mesoscale.

#### 4.2.4 Characterization

Very little is done in the establishing of magnetic responses of curvilinear antiferromagnets.

*Magnetometry*: Integral measurements of antiferromagnets can be routinely applied to get such material information as spin‐flop/spin‐flip fields and temperature dependency of magnetic susceptibility.^[^ ^104^, ^426^ ^]^ These techniques could be readily applied to measure curvilinear antiferromagnets. Furthermore, the use of scanning SQUID,^[^ ^611^ ^]^ cantilever magnetometry,^[^ ^267^, ^285^, ^286^, ^612^ ^]^ or NV magnetometry^[^ ^352^, ^579^ ^]^ can provide similar measurements even of individual curvilinear antiferromagnetic objects. The validation of these measurements is still pending.

*Tomography*: In contrast to ferromagnets,^[^ ^291^, ^613^, ^614^ ^]^ the complete apparatus of the vector tomography is not established for antiferromagnets taking into account the director property of the order parameter. This concerns not only experimental methods on how to make slices for the tomographic reconstruction, but also a reconstruction software. Promising is to use the X‐ray spectro‐holography,^[^ ^289^, ^323^ ^]^ which can be extended also to time resolved measurements.

*Microscopy*: The choice of the imaging technique of curvilinear antiferromagnetic textures is dependent on the type of the ordering of ***n***. Optical methods represent a powerful technique to access antiferromagnetic domains.^[^ ^615^ ^]^ The orientational domain walls (***n*** are turned by 90°) can be accessed via X‐ray magnetic linear dichroism (XMLD).^[^ ^530^ ^]^ The so‐called phase domains (the domain wall texture is different in one layer shift of the magnetic lattice) can be detected by this technique indirectly only if the wide enough domain walls are present. This can be solved precise measurements using SP‐STM providing direct access to the direction of magnetic moments of individual atoms^[^ ^468^, ^616^, ^617^ ^]^ or by the detection of surface stray fields via single NV microscopy.^[^ ^352^, ^579^ ^]^ One can expect that both, XMLD and NV‐based imaging methods will be useful for the visualization of 2D corrugated antiferromagnetic shells where both types of n‐domains are expected. Although potentially feasible the electron vortex beam technique^[^ ^618^, ^619^ ^]^ can be extended to the curvilinear antiferromagnetic architectures.

The methods of using magnetoresistance (e.g., planar, anomalous, and spin Hall effects) are expected to be directly applied to metallic antiferromagnets.^[^ ^127^, ^620^, ^621^, ^622^, ^623^, ^624^ ^]^ Still, many antiferromagnets are insulating oxides. In this case, the magnetic states can be accessed by transport measurements exploring the proximity of a heavy metal (titanium or platinum), as it already done for planar samples,^[^ ^579^, ^621^, ^625^, ^626^, ^627^, ^628^, ^629^, ^630^, ^631^ ^]^ or by applying voltage‐induced magnetic anisotropy gradient.^[^ ^632^ ^]^

## 5 Curvilinear Superconductors

Superconductors are materials which are characterized by vanish of electrical resistance and expulsion of magnetic flux from their interior when cooled below some characteristic temperature *T*_c_, which is called the superconducting transition temperature. Like ferromagnetism, superconductivity is a phenomenon which can only be explained by quantum mechanics. At the same time, the expulsion of magnetic field lines from the interior of the material in the superconducting state—the Meissner effect^[^ ^67^ ^]^—makes the magnetic response of superconductors very distinct from the response of the materials considered in previous sections.

More precisely, the complete expulsion of magnetic flux only occurs in the bulk of so‐called type I superconductors, in which the external magnetic field penetrates (and decays in) a relatively thin sheath near their surface. The spatial scale of the decay of the external magnetic field is determined by the magnetic penetration depth λ, which also sets the length scale within which the Meissner currents circulate to screen the interior of the superconductor from the applied magnetic field. Nevertheless, superconductors at the nanoscale and the majority of technologically relevant superconductors are so‐called type II superconductors. In the presence of moderately strong magnetic fields, they exhibit a very special phase which is called the mixed state or the Shubnikov phase.^[^ ^633^ ^]^ In this phase, the superconductor is thread by an array of magnetic flux lines (Abrikosov vortex lattice^[^ ^634^ ^]^) which can be put in motion by the action of an external transport current.^[^ ^633^ ^]^ Each vortex (fluxon) carries one magnetic flux quantum *Φ*_0_ = 2.068 × 10^−15^ T m^2^ so that vortices can be viewed as tiny cylinders of the material in the normally conducting state (vortex cores), which are surrounded by whirls of the supercurrent and which interact with each other, with structural imperfections, with sample boundaries, and so on.^[^ ^64^ ^]^

Within the framework of the Ginzburg–Landau theory,^[^ ^67^ ^]^ superconductivity is described with the complex order parameter ψ = |ψ|*e* ^*i*θ^ with the phase θ and the squared modulus |ψ|^2^ indicating the fraction of electrons which can transfer electric charge without dissipation. The reduction of |ψ| to zero at the normal‐state vortex cores makes the phase θ indefinite and allows the phase to slip by 2π in dynamic resistive states.^[^ ^67^ ^]^ In other words, both vortices and phase slips can be viewed as topological defects of the superconducting order parameter whose evolution in space and time determines the magneto‐resistive properties of superconductors.

The extension of superconductors to curvilinear geometries brings about geometry‐induced magnetic field variations and topological transitions in patterns of the circulating currents, which do not occur in planar systems.^[^ ^63^ ^]^ Mapped to the magneto‐resistive response, this allows one to anticipate fundamentally novel topology‐ and geometry‐induced phenomena and potential applications of curvilinear superconductors.^[^ ^76^ ^]^

In a broader context, the interaction of topological defects in thin films on curvilinear substrates is a fascinating venue to develop and examine sophisticated theoretical models in various fields of science beyond superconductivity, ranging from superfluid helium over biological structures to symmetry‐ and topology‐driven design of interfaces with prescribed functionality.^[^ ^81^ ^]^ It should be noted that nontrivial topology considered in what follows occurs due to a special geometry of the structures or fields in the real space.^[^ ^635^ ^]^ Exemplary structures include superconducting rings, cylinders, spheres, Möbius strips, and tori. At the same time, topologically protected surface/edge states in materials can appear due to Dirac physics and/or topologically nontrivial electronic structure in the momentum space giving rise to topological superconductivity. While topological superconductivity represents a topical area of quantum matter which currently attracts great attention, this domain is beyond our consideration and the reader is referred to dedicated reviews^[^ ^636^, ^637^, ^638^ ^]^ and original works.^[^ ^639^, ^640^, ^641^, ^642^, ^643^, ^644^ ^]^ In what follows, a brief mentioning of topological superconductors will be restricted to those in curvilinear geometries.

### 5.1 State of the Art

The combination of reduced dimensionality with curvilinear geometry and nontrivial topology has been proved to be a rich source of emerging physics. In this respect, multiply connected systems are valuable platforms for investigations of quantum phenomena such as gauge invariance and quantum interference in conducting and superconducting systems. The Aharonov–Bohm^[^ ^645^ ^]^ and Little‐Parks^[^ ^646^, ^647^ ^]^ effects are prominent examples of coherent quantum phenomena occurring in multiply connected systems.^[^ ^648^ ^]^ The Aharonov–Bohm effect leads to deflections of circular orbits of vortices and antivortices on the surface of hollow superconducting cylinders, giving rise to resistance oscillations.^[^ ^649^ ^]^ Recently, Little–Parks resistance oscillations have provided evidence that superconductivity can be induced by electrochemical doping in WS_2_ chiral nanotubes.^[^ ^650^ ^]^ In this system, superconductivity is not reciprocal as the forward and backward supercurrent flows are not equivalent because of the broken inversion symmetry.

**Figure 14:**
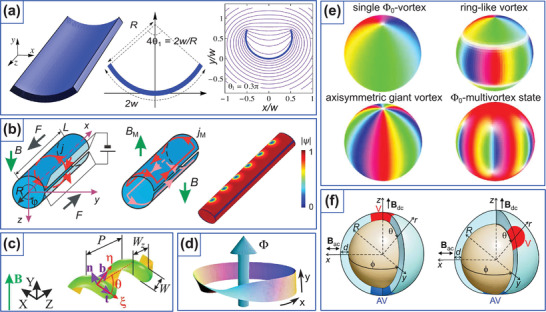
Schematics of curvilinear superconductors. a) Curved tape and the magnetic field lines around it. Reproduced with permission.^[^ ^654^ ^]^ Copyright 2009, American Physical Society. The cross section of the tape has a shape of a circular arc of radius *R* with the central angle 4θ_1_ = 2*w*/*R*, where 0 < θ_1_ = *w*/2*R* < π/2. b) Left: Scheme of the open superconducting tube. The red arrows indicate the direction of the transport current. The electrodes, which are connected to both sides of a slit, are shown semitransparent. Middle: Scheme of two systems of the Meissner currents $j_{M}$ in the tube. Right: Equilibrium distribution of the superconducting order parameter at Φ/*Φ*_0_ = 8.5 for a tube of radius *R* = 280 nm and length *L* = 3.5 μm. Reproduced with permission.^[^ ^62^ ^]^ Copyright 2012, American Chemical Society. c) Schematics and geometrical parameters (see the text) of a helical spiral coil. Reproduced with permission.^[^ ^678^ ^]^ Copyright 2017, IOP Publishing. d) A superconducting Möbius strip with flux *Φ* threading the ring. Reproduced with permission.^[^ ^692^ ^]^ Copyright 2005, American Physical Society. e) Vortex structures on a spherical shell. Reproduced with permission.^[^ ^685^ ^]^ Copyright 2008, American Physical Society. The amplitude and phase of the order parameter are shown as the saturation and hue of the color scale, respectively. The vortex core regions correspond to white areas. f) Nanoshells with a uniform (left) and nonuniform (right) thickness. The red and blue shadows schematically indicate a vortex (V) and an antivortex (AV). Reproduced with permission.^[^ ^686^ ^]^ Copyright 2012, Elsevier.

In the following, we will outline some of the effects peculiar to particular curvilinear geometries, as illustrated in **Figure** **14**. For an overview of the theoretical approaches and experimental techniques employed for investigations of curvilinear superconductors we refer to **Table** **1**.

**Table 1:** A selection of theoretical approaches and experimental techniques employed for investigations of curvilinear superconductors. TDGL: Time‐dependent Ginzburg–Landau (equation); LAMH: Langer‐Ambegaokar––McCumber–Halperin (theory); MOD: metal–organic decomposition; MOCVD: metal–organic chemical vapor deposition; RABITS: rolling‐assisted biaxial texturized substrates; HFSS: high‐frequency structure simulations; FIBID: focused ion beam induced deposition

**Geometry**	**Type of study**	**Technique/approach, features**	**References**
Ring	Theory	Usadel equation, S/F structure	[651]
	Theory and experiment	TDGL equation	[652]
	Theory and experiment	Tinkham's approach, e‐beam lithography	[653]
Curved strip	Theory	Complex field approach	[654, 655]
	Theory	TDGL equation	[656]
Cylinder	Theory	TDGL equation	[649, 656, 657]
	Theory	TDGL equation, cylinder with slit	[62, 63, 658]
	Theory	TDGL and London equations, LAMH theory	[659]
	Theory	TDGL equation, Runge–Kutta method	[660]
	Theory	Complex field approach	[661]
	Theory	COMSOL	[662]
	Experiment	He^+^ FIBID	[663]
	Experiment	Ionic gating in chiral nanotubes of WS_2_	[650]
	Experiment	MOD, MOCVD of LZO and YBCO on RABITS	[664]
	Experiment	Rolled‐up technology, hybrid rolled‐up microtubes	[28, 665]
	Experiment	Evaporation on rotating filaments	[666]
	Theory and experiment	Maxwell equations	[667]
	Theory and experiment	TDGL equation, evaporation on rotating filaments	[668]
	Theory and experiment	London equation, BCS theory, evaporation on cylindrical formers	^[^ ^669^ ^]^
Spiral	Experiment	E‐beam lithography	[670]
	Experiment	E‐beam lithography	[671]
	Theory and experiment	HFSS, photolithography	[672, 673]
	Theory and experiment	Impedance matrix, photolithography	[674, 675]
Helix	Theory	The model of Garber et al.^[^ 676 ^]^	[677]
	Theory	TDGL equation	[678]
	Experiment	Rolled‐up technology, COMSOL for self‐heating	[29]
	Theory and experiment	TDGL equation, He^+^ FIBID	[30]
Sphere	Theory	Bardeen–Stephen equation	[679]
	Theory	London equation	[680]
	Theory	Monte‐Carlo simulations	[681]
	Theory	TDGL equation	[10, 682, 683, 684, 685, 686, 687]
	Experiment	Seeded‐melt growth	[688]
	Experiment	Binding of microparticles in strong electric fields	[689]
	Experiment	Solvothermal synthesis	^[^ ^690^ ^]^
Möbius strip	Theory	TDGL equation	[691, 692]
	Experiment	Crystal growth using a spherical droplet as a spool	[693]

Among curvilinear superconductors, the most extensively studied systems are represented by thin‐walled cylinders.^[^ ^28^, ^62^, ^63^, ^649^, ^650^, ^656^, ^657^, ^658^, ^659^, ^660^, ^661^, ^662^, ^663^, ^664^, ^665^, ^666^, ^667^, ^668^, ^669^ ^]^ There are a few works addressing helices^[^ ^29^, ^30^, ^677^, ^678^ ^]^ and Möbius strips^[^ ^691^, ^692^, ^693^, ^694^, ^695^ ^]^ while an extensive series of theoretical works is concerned with spherical shells.^[^ ^10^, ^679^, ^680^, ^681^, ^682^, ^683^, ^684^, ^685^, ^686^, ^687^, ^690^, ^696^, ^697^, ^698^, ^699^, ^700^ ^]^

- Curvilinear superconducting tapes: From an application point of view, great attention has been devoted to curvilinear superconducting tapes.^[^ ^701^ ^]^ The reason for this is that in power transmission cables, superconducting tapes are wrap around a cylindrical former and the tapes can be curved conforming the shape of the cylinder.^[^ ^655^, ^664^ ^]^ In particular, theoretical investigations revealed that bending a superconducting tape leads to a reduction of the magnetic field acting perpendicular to the tape that has been suggested as a route to reduction of ac losses.^[^ ^654^, ^655^ ^]^ For a thin‐film superconducting tubular wire, analytical expressions for the magnetic‐field and current distributions have been obtained^[^ ^661^ ^]^ and time‐dependent electric fields with a trapped vortex have been shown to be very similar to the ones exhibited by resistively shunted Josephson junctions.^[^ ^657^ ^]^ Curved thin sub‐micrometer‐sized stripes conforming to cylindrical shells have been theoretically investigated with emphasis on nucleation^[^ ^660^ ^]^ and thermal activation^[^ ^659^ ^]^ of phase‐slip lines. Phase separation near a quantum phase transition^[^ ^666^ ^]^ and destruction of the global phase coherence^[^ ^668^ ^]^ have been studied in ultrathin hollow cylinders.
- Spiral‐shaped planar structures find applications in superconducting nanowire single‐photon detectors in the optical and near‐infrared range,^[^ ^670^, ^671^, ^702^ ^]^ as well as in superconducting resonators and metamaterials.^[^ ^673^, ^703^, ^704^ ^]^ The absence of sharp corners in the detector layout reduces current‐crowding effects while the spiral geometry allows the detection efficiency to be independent of the polarization of light.^[^ ^670^, ^671^, ^702^ ^]^ Spiral planar elements produce a flat spectral response of hot‐electron bolometers in the THz frequency range^[^ ^705^ ^]^ and allow for building low‐loss radio frequency metamaterials and resonators.^[^ ^673^, ^704^ ^]^ Macroscopic superconducting tapes helically wound around a cylinder have also been intensively explored for improving the performance of power transmission cables.^[^ ^676^, ^677^ ^]^
- Topological superconductors in curvilinear geometries: In the recent years, a great deal of experimental and theoretical work has been concerned with topological superconductors, see for example, recent works^[^ ^639^, ^640^, ^641^, ^642^, ^643^, ^644^ ^]^ and references therein. This interest is largely driven by the possibility to generate Majorana modes at the ends of 1D topological superconductors.^[^ ^637^ ^]^ Majorana quasi‐particles possess a property of being their own antiparticles, that makes Majorana modes topologically protected and particularly attractive for decoherence‐immune and fault‐tolerant quantum computing.^[^ ^706^ ^]^ In 2D superconductors, the presence of such Majorana modes is determined by the amount of flux piercing the superconductor at a vortex: an odd number of superconducting flux quanta (unit flux) gives a Majorana, while an even number does not. Since Majorana modes can only come in pairs, an odd total number of unit flux vortices requires the presence of a Majorana mode at the boundary of the material. Recently, it has been shown that, in the absence of any flux, the ground state on the annulus does not support Majorana modes while the one on the cylinder does.^[^ ^695^ ^]^ A nonorientable Möbius strip, which has only one edge, has been shown to necessarily have a defect line along the center of the Möbius band to support edge Majorana modes.^[^ ^695^ ^]^ Conceptually, this is similar to topologically protected domain walls in ferromagnetic Möbius strips.^[^ ^31^ ^]^ Recent proposals have predicted the possibility to realize topological superconductivity by hybridizing ordinary superconductors with helical materials, with the help of magnetic perturbations. The curved geometric profile also allows for tuning the spin correlations of the superconducting state via the induced inhomogeneity of the spin‐orbit coupling that affects the Josephson critical current^[^ ^707^ ^]^ and, in curved nanostructures with Rashba spin‐orbit coupling, leads to nontrivial textures of spin‐triplet pairs.^[^ ^11^ ^]^ Due to the geometric Meissner effect, 2D chiral superconductors on curved surfaces have been shown to spontaneously develop a magnetic flux.^[^ ^708^ ^]^ This effect has been employed to shed light on the location of zero‐energy Majorana modes and it provides an unequivocal signature of chiral superconductivity.^[^ ^708^ ^]^

#### 5.1.1 Theoretical Description: TDGL Equation

Theoretical description of curvilinear superconductors requires the analysis of the evolution in space and time of screening currents and topological defects of the superconducting order parameter.^[^ ^67^ ^]^ An overview of the analytical models and numerical methods applied to curvilinear superconductors is given in Table 1. For most of the curvilinear geometries illustrated in Figure 14, numerical simulations can be done relying upon the time‐dependent Ginzburg–Landau (TDGL) equation^[^ ^709^ ^]^ and render this approach as the most versatile one. The TDGL represents a reasonable compromise between an approximate/phenomenological and an exact/microscopic description of the dynamics of topological defects in superconductors and provides a “mesoscopic bridge” between micro and macro scales.^[^ ^710^ ^]^ The TDGL catches the most essential features of vortex matter, taking into account all interaction energies for arbitrarily shaped vortices as well as the interaction of vortices with pinning sites.^[^ ^710^ ^]^ Far from the critical temperature *T*_c_, the GL equations do not correctly reproduce the physics in the vortex core, but still describe the interaction between vortices correctly.^[^ ^711^ ^]^ We note that while the microscopic derivation of the TDGL was originally done for a gapless superconductor with paramagnetic impurities,^[^ ^712^ ^]^ the TDGL is also widely used for studying various aspects of current‐driven vortex matter in gapped superconductors with nonmagnetic impurities^[^ ^710^, ^711^ ^]^ including the vortex dynamics at high vortex velocities^[^ ^713^, ^714^ ^]^ and at GHz ac frequencies.^[^ ^715^, ^716^ ^]^ For in‐depth discussions of the applicability of the TDGL and its mathematical aspects we refer to refs. ^[^ ^710^, ^711^, ^715^, ^717^ ^]^.

In this subsection we outline the most essential features of the TDGL equation following the monograph by Fomin^[^ ^76^ ^]^ on topological and geometrical effects in self‐rolled micro‐ and nanoarchitectures.

The TDGL equation is a differential equation which relates the spatial variation of the superconducting order parameter ψ(r,t) to the magnetic vector potential A(r) and the current with the density j(r) in a superconductor. The equation reads

(42) $$\frac{\hbar^{2}}{2m^{*}D}\frac{\partial \psi }{\partial t}-\frac{\hbar^{2}}{2m^{*}}{\left(\nabla +\frac{2e}{\hbar }iA\right)}^{2}\psi +\alpha \psi +\beta {\left|\psi \right|}^{2}\psi =0$$

In Equation (42), *ℏ* is the reduced Planck constant, m* is the effective mass and (−2*e*) is the charge of a particle (Cooper pair) carrying the superconducting current, *e* is the elementary electron charge, *D* is the diffusion coefficient, and ***A*** the vector potential of the magnetic field. The functions α and β depend on the temperature *T*

(43) $$\begin{array}{l} \alpha (T)=\alpha_{0}\left(\frac{T}{T_{c}}-1\right),\;\;\alpha_{0}>0\\ \beta (T)>0,\;\;\;\;\;\;\beta (T)≅\beta \end{array}$$

such that α(*T*) vanishes at the superconducting transition temperature *T*_*c*_ and is negative at *T* < *T*_c_.

The function α(*T*) can be expressed in terms of the coherence length ξ(*T*)

(44) $$\alpha (T)=-\frac{\hbar^{2}}{2m^{*}}\xi^{2}(T)$$

and the coefficient β is represented as

(45) $$\beta =\frac{2e^{2}\mu_{0}\hbar^{2}\xi^{2}(T)\lambda^{2}}{{\left(m^{*}\right)}^{2}}$$

where μ_0_ is the magnetic permeability of free space and λ is the London penetration depth

(46) $$\lambda^{2}=-\frac{m^{*}\beta }{4e^{2}\mu_{0}\alpha (T)}\equiv \frac{{\left(m^{*}\right)}^{2}\beta }{2e^{2}\mu_{0}\hbar^{2}\xi^{2}(T)}$$

It is convenient to use a dimensionless form of Equation (42), such as

(47) $$\frac{\partial \psi }{\partial t}={\left(\nabla -iA\right)}^{2}\psi +2\kappa^{2}\psi (1-{\left|\psi \right|}^{2})$$

where κ = λ/ξ is the Ginzburg–Landau parameter, and the dimensional units for the physical quantities are defined after:^[^ ^718^, ^719^ ^]^ Time is measured in units of 2λ^2^/*D*, length in $\sqrt{2}\lambda$, magnetic field in *Φ*_0_/(4*πλ*), magnetic flux in *Φ*_0_, and current density in $\Phi_{0}c/(8\sqrt{2}\pi^{2}\lambda^{3})$.

The TDGL must be completed with the appropriate boundary conditions, which include the geometry of confinement in the analysis. In this connection, a proper choice of the coordinate system is an essential issue. In what follows, the application of the TDGL for numerical simulations of superconducting systems with nontrivial topology will be illustrated by two examples.

*Rolled‐Up Superconducting Nanomembrane*: The first example is an open superconductor nanotube (with a narrow paraxial slit along the tube) of length *L* and of radius *R*, as shown in Figure 14b. When the thickness of the membrane is significantly smaller than the effective penetration depth, the 2D approximation can be applied. The magnetic field inside the membrane is approximated by the applied external magnetic field ***B***. The component of the magnetic field perpendicular to the surface is strongly inhomogeneous: *B*_*n*_(φ) = −*B*sin(φ), where φ is the azimuthal angle with φ = 0° at the position of the slit. The effective magnetic flux is equal to *Φ* = 2*RLB*. The TDGL equation is invariant under the gauge transformation. The chosen Landau gauge sets the vector potential to $A=Ae_{x}$ with *A* = −*By*.

The absence of the supercurrent component perpendicular to the surface of the membrane determines the first boundary condition

(48) $$(\nabla -iA)\psi {|}_{n,\text{boundary}}=0$$

Since two electrodes are attached to the sides of the slit in order to generate a current passing through the circumference of the nanotube, the supercurrent component perpendicular to the surface of the membrane has to match the generated transport current $j_{tr}$ at the edges of the slit. This imposes the second boundary condition

(49) $$\left[\nabla -i\left(A+\frac{j_{tr}}{{\left|\psi \right|}^{2}}\right)\right]\psi {|}_{n,\text{electrode}}=0$$

**Figure 15:**
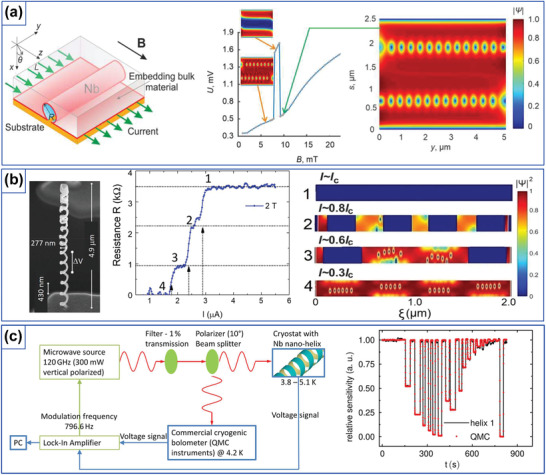
Simulations, electrical resistance and microwave measurements of curvilinear superconductors. a) Left: Geometry of the open superconducting tube. Middle and right: Simulated current–voltage curve of the tube with the different configurations of the topological defects. a) Reproduced under the terms of the CC‐BY Creative Commons Attribution 4.0 International license (https://creativecommons.org/licenses/by/4.0).^[^ ^63^ ^]^ Copyright 2020, The Authors, published by Springer Nature. b) Left: SEM image of a W–C nanohelix. Middle: Experimental resistance versus current curve for the nanohelix. Right: Simulated order parameter distribution plotted over the 2D surface of the helical structure for the magnetic field 2 T. Different vortex and phase‐slip patterns correspond to steps of resistance. b) Adapted with permission.^[^ ^30^ ^]^ Copyright 2019, American Chemical Society. Further permission related to the figure excepted should be directed to the ACS. c) Left: Sketch of the measurement setup for microwave detection at 120 GHz using a Nb microhelix shown in Figure 16c. One microwave path is going to the nanohelix and the other is illuminating the QMC bolometer. Comparison of time‐dependent measurements of the nanohelix with the QMC sensor using normalized sensitivity (1 is equal to 300 mW output power of the microwave source). c) Reproduced with permission.^[^ ^29^ ^]^ Copyright 2019, American Chemical Society.

Various TDGL custom codes and solvers (e.g., COMSOL^[^ ^715^ ^]^) can be employed for the implementation of numerical simulations. In what follows we are going to illustrate simulation results obtained in a 2D approximation with a mesh of the nanotube by using an adaptive finite‐difference calculation method.^[^ ^62^ ^]^ Application of the transport current allows one to proceed from the static configuration of topological defects to their dynamics under the action of the transport current.^[^ ^62^, ^63^ ^]^ This implies the addition of the term −*iκφψ* in the right‐hand side of Equation (47), where φ is the electric scalar potential which is found as a solution of the Poisson equation coupled with the TDGL equation

(50) $$\Delta \varphi =\frac{1}{\sigma }\nabla \cdot j_{sc}$$

where the superconducting current density is defined as $j_{sc}=\frac{1}{2i\kappa }\left(\psi^{*}\nabla \psi -\psi \nabla \psi^{*}\right)-A|\psi {|}^{2}$ and σ is the normal conductivity. An exemplary magnetic‐field‐voltage curve simulated for a Nb tube of radius *R* = 280 nm and length *L* = 3.5 μm^[^ ^63^ ^]^ is presented in **Figure** **15**a. The generated voltage reveals a sequence of different order parameter states occurring with increase of the magnetic field: a pure superconducting state, a mixed state (vortices and the superconducting state), a phase‐slips state (voltage peak regime) and, again, a mixed state. The voltage jumps are associated with the topological transitions between the phase‐slip and vortex‐chain regimes. The topological transitions are induced by the nontrivial topology of superconducting (Meissner) currents,^[^ ^63^ ^]^ as shown in the middle panel in Figure 14b.

*Superconducting Helical Coil*: The second example is a superconducting helical coil of radius *R*, width *W*, length *L*, and pitch *P*. In order to define the boundary conditions and enable an easier way to solve the TDGL equation, the coordinate system (ξ, η) is considered, see Figure 14c. One notes that in the Cartesian coordinates *X*, *Y*, *Z*, the position vector at any point on a 1D helix (*W* = 0) is defined as

(51) $$r(\xi )=\left\{R\cos\left(\frac{2\pi }{l}\xi \right),R\sin\left(\frac{2\pi }{l}\xi \right),\frac{P}{l}\xi \right\}$$

where the length of a single turn is $l=\sqrt{{\left(2\pi R\right)}^{2}+P^{2}}$ and ξ is the longitudinal coordinate along the helix. The introduction of the helix angle θ=arctan(2πR/P) between the axis running through the middle of the helix and the tangential vector on the helix surface with 2π*R*/*l* = sinθ and *P*/*l* = cosθ allows one to define (see Figure 14c) the tangential ***t***, normal ***n***, and binormal ***b*** unit vectors

(52) $$\begin{array}{l} t(\xi ,\eta )=\frac{\partial r(\xi ,\eta )}{\partial \xi }=\left\{-\sin\theta \sin\left(\frac{2\pi }{l}s\right)\;\;,\sin\theta \cos\left(\frac{2\pi }{l}s\right)\;\;,\cos\theta \right\}\\ n(\xi ,\eta )=\frac{\partial r(\xi ,\eta )}{\partial R}=\left\{\cos\left(\frac{2\pi }{l}s\right)\;\;,\sin\left(\frac{2\pi }{l}s\right)\;\;,0\right\}\\ b(\xi ,\eta )=\left\{\cos\theta \sin\left(\frac{2\pi }{l}s\right)\;\;,-\cos\theta \cos\left(\frac{2\pi }{l}s\right)\;\;,\sin\theta \right\} \end{array}$$

in the new (ξ, η) coordinate system

(53) $$\begin{array}{l} \xi =R\phi \sin\theta +z\cos\theta \\ \eta =-R\phi \cos\theta +z\sin\theta \end{array}$$

Introducing the longitudinal *A*_ξ_ = *A*cosθ and transverse *A*_η_ = *A*sinθ vector potential components, the Laplace operator in this coordinate system reads

(54) $${\left(\nabla -iA\right)}^{2}={\left(\frac{\partial }{\partial \xi }-iA_{\xi }\right)}^{2}+\;\;{\left(\frac{\partial }{\partial \eta }-iA_{\eta }\right)}^{2}$$

The boundary conditions written in the terms of *A*_ξ_ and *A*_η_ are now explicitly independent from each other

(55) $$\begin{array}{c} \left(\frac{\partial }{\partial \eta }-iA_{\eta }\right)\psi (\xi ,\pm W/2){|}_{\text{boundary}}=0\;\;\text{at}\;\;\text{any}\;\;\xi \\ \left(\frac{\partial }{\partial \xi }-iA_{\xi }\right)\psi (0\;\text{or}\;L,\eta ){|}_{\text{boundary}}=0\;\;\text{at}\;\;\text{any}\;\;\eta \end{array}$$

This property of the boundary conditions makes the coordinates introduced by Equation (53) particularly useful for solving the TDGL equation on a helical stripe.

An interesting feature of the measured resistance‐current characteristics of a superconductor nanohelix^[^ ^30^ ^]^ is that the resistance transition occurs in a series of steps, as observed in measurements under fixed perpendicular magnetic field, see Figure 15b. The TDGL simulation results reveal several patterns of the order parameter corresponding to the spatial distribution of the normal to the surface component of the magnetic field over the surface of the helical nanostructure. These patterns are represented in the right panel of Figure 15b. The experimentally observed jumps (larger than 100 Ω) in the resistance–current characteristics are about two orders of magnitude larger than the resistance induced by an individual vortex, which follows from the numerical simulations. The most probable reason for the observed jumps is therefore the occurrence of phase slips, which start to appear at transport current values indicated by the arrows in Figure 15b. The transition of two half‐turns of the nanohelix into the full phase‐slip regime causes the resistance to increase by about 1 kΩ, while the presence of the phase slip in all half‐turns without the vortex dynamics results in a resistance of about 2.25 kΩ. In this way, in addition to the description of the series of jumps in the resistance‐current characteristics, TDGL simulation results reveal the coexistence of topological defects of different types in superconducting 3D nanohelices.

#### 5.1.2 Fabrication and Characterization

**Figure 16:**
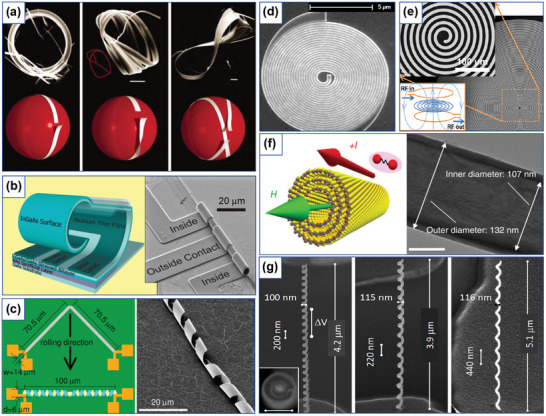
Examples of curvilinear superconductors. a) SEM images of three types of topology of NbSe_3_ crystals (white ribbons) twisted around selenium droplets (red spheres). Ring structure (0π twist), Möbius strip (1π twist) and figure‐of‐eight strip (2π twist). Scale bars are 10 μm. Reproduced with permission.^[^ ^693^ ^]^ Copyright 2002, Springer Nature. b) A 3D schematic of the rolled up Josephson junction (left) and the exposed metal structure after etching away the semiconductor layer (right). Reproduced with permission.^[^ ^28^ ^]^ Copyright 2010, American Chemical Society. c) Niobium stripe before rolling (upper part) and rolled up (lower part). SEM image of a rolled‐up superconducting nanohelix (right). Reproduced with permission.^[^ ^29^ ^]^ Copyright 2019, American Chemical Society. d) SEM image of a spiraling NbTiN detector. Reproduced with permission.^[^ ^670^ ^]^ Copyright 2008, AIP Publishing. e) SEM image of a spiral‐shaped superconducting resonator made of a NbN film. Reproduced with permission.^[^ ^704^ ^]^ Copyright 2019, AIP Publishing. f) Illustration of unidirectional electric transport and an TEM image of a single WS_2_ chiral nanotube. Adapted under the terms of the CC‐BY Creative Commons Attribution 4.0 International license (https://creativecommons.org/licenses/by/4.0).^[^ ^650^ ^]^ Copyright 2017, The Authors, published by Springer Nature. g) 3D W–C nanohelices fabricated by focused He^+^ ion beam induced deposition. Adapted with permission.^[^ ^30^ ^]^ Copyright 2019, American Chemical Society. Further permission related to the figure excepted should be directed to the ACS.

Fabrication and characterization of curvilinear superconductors at the micro‐ and nanoscale has so far been limited to a few experimental techniques, most of them being rather delicate, see **Figure** **16**. While ring‐shaped and cylindrical samples of rather large dimensions were extensively investigated in early works, more sophisticated techniques have been developed to make accessible samples of more complex geometries. For instance, millimeter‐sized spherically shaped YBCO granules can be obtained by, for example, seeded‐melt growth^[^ ^688^ ^]^ or be formed by binding of millions of micrometer‐sized particles in strong electric fields.^[^ ^689^ ^]^ At the same time, much less experimental work has so far been concerned with hollow micro‐ and nanosized superconducting samples. In particular, multi‐crystalline isotropic Pb hollow sphere‐like samples have been produced by a solvothermal synthesis.^[^ ^690^ ^]^ Magnetic measurements revealed that the produced samples with an inner diameter of 400 nm are superconducting with a transition temperature *T*_c_ of 11.05 K.^[^ ^690^ ^]^ For the characterization of such submicrometer‐large samples, micro‐Hall magnetometry,^[^ ^281^ ^]^ nano‐SQUID,^[^ ^364^ ^]^ SQUID‐on‐tip,^[^ ^720^ ^]^ and microwave inductive sensing^[^ ^293^, ^721^, ^722^, ^723^ ^]^ techniques should, in principle, be applicable. However, reports on the characterization of micro‐ and nanoscale curvilinear superconductors remain rather rare.^[^ ^29^, ^650^, ^663^ ^]^

### 5.2 Perspectives of Curvilinear Superconductors

#### 5.2.1 Self‐rolled and Direct‐Write 3D Nanoarchitectures

At present, experimental investigations of nanoscale curvilinear superconductors remain a challenge. This is caused by the lack of fabrication techniques for the realization of 3D shell structures with a wall width of the order of the superconducting coherence length being in the range of a few to a few tens of nm for most of thin‐film low‐temperature superconductors. Therefore, the few advanced techniques which can be employed for the fabrication of curvilinear superconductors at the micro‐ and nanoscale should be mentioned especially. Among these techniques, high‐tech self‐assembly based on the strain‐driven self‐rolling of semiconductor nanomembranes^[^ ^228^, ^665^ ^]^ has demonstrated a strong potential for the experimental realization of microscale superconducting cylindrical^[^ ^28^ ^]^ and helical systems.^[^ ^29^ ^]^ Alternative techniques are direct‐write nanofabrication technologies relying upon fabrication and post‐growth processing of nanostructures by focused electron and ion beams.^[^ ^139^, ^141^, ^721^, ^724^ ^]^ In the recent years, these techniques have reached a high level of maturity needed for the fabrication of 3D nanoarchitectures.^[^ ^142^, ^143^, ^243^, ^244^, ^277^, ^278^, ^279^ ^]^ In particular, fabrication of hollow superconducting nanoscale cylinders as small as 32 nm in diameter using focused He^+^ ion beam induced deposition (FIBID) has recently been demonstrated.^[^ ^663^ ^]^ The obtained wall thicknesses down to about 12 nm represent an important achievement in the direct‐write 3D nanofabrication. The availability of superconducting nanoscale cylinders provides access to experimental investigations of numerous theoretical predictions lacking experimental examination so far. For instance, the effects of surface curvature on vortex dynamics have recently been analyzed for curved stripes^[^ ^656^ ^]^ and hollow microtubes.^[^ ^62^ ^]^ Cylindrical sections exhibiting asymmetric transport properties have been suggested as superconducting current rectifiers^[^ ^656^ ^]^ and a tunable generation of correlated vortices in superconductor tubes with nanoslits has been predicted.^[^ ^62^ ^]^ In the latter system, intervortex correlations lead to an attraction between vortices moving at opposite sides of a tube^[^ ^725^ ^]^ and the vortex nucleation period can be branched in the presence of an inhomogeneous transport current.^[^ ^658^ ^]^ Experimental examination of these effects may lead to the development of 3D fluxonic circuits and novel fluxon‐based computing approaches.

There has been a series of works addressing the role of the system topology on the superconducting properties in curvilinear geometries. For instance, in nonorientable systems such as a Möbius strip, unusual vortex states have been predicted.^[^ ^692^ ^]^ Though an elegant approach for synthesizing rings, Möbius strips and figure‐of‐eight strips of single crystal NbSe_3_ by chemical‐vapor transport was demonstrated back in 2002,^[^ ^693^ ^]^ experimental realization of Möbius nanostrips represents a challenge yet to be met. Very recently, ferromagnetic Möbius nanostrips have been successfully fabricated by focused electron beam induced deposition (FEBID)^[^ ^279^ ^]^ suggesting that the direct‐write nanofabrication technologies of FEBID and FIBID are likely to become the techniques of choice for the fabrication of superconducting spheres and Möbius nanostrips. At the same time, further advancements in materials science are required to make accessible superconducting spherical nanoshells.

#### 5.2.2 Applications of Superconducting 3D Nanoarchitectures

Investigations of 3D helical superconducting nanoarchitectures remain challenging for both theory and experiment. While TDGL simulations of microsized helical stripes are at the edge of the current computation capabilities, various aspects of TDGL simulations in 3D geometries are extensively discussed.^[^ ^715^, ^726^, ^727^ ^]^ Recently, vortex patterns in nanostructured microhelices have been considered and quasi‐degeneracy of vortex patterns has been revealed in the helical coils when the number of vortices is incommensurable with the total number of half‐turns.^[^ ^678^ ^]^ Experimentally, free‐standing superconducting Nb nanohelices have recently been fabricated from a 50 nm‐thick Nb film rolled up into helices with diameters down to 6 μm,^[^ ^29^ ^]^ see Figure 16c. Importantly, such nanohelices have a negligible thermal contact to the substrate that has allowed for their use as sensitive transition‐edge sensors for the detection of microwave radiation. In the measurement setup presented in Figure 15c, the lock‐in amplifier gives a modulation frequency to the microwave source. The microwave source has an output of 300 mW at 120 GHz. The power of the vertical polarized and modulated microwave is reduced to 1% by a filter and then split by the polarizer into two directions. One path is going to the nanohelix and the other is illuminating a commercially available QMC bolometer. The voltage signal from both devices is measured with the lock‐in amplifier. The best working point (highest sensitivity) for the nanohelix detector, which works as a transition‐edge sensor (TES), corresponds to the steepest change of its resistance as a function of the applied current. The right panel in Figure 16c shows the time‐dependent measurements of the normalized response signal of the nanohelix in comparison to the QMC sensor. Both detectors (nanohelix and QMS sensor) exhibit the same relative response with microwave power variation. Given the nanohelix footprint area 600 μm^2^ with that of the QMC (25 mm^2^), a detailed analysis^[^ ^29^ ^]^ reveals that although the nanohelix has a worse signal‐to‐noise ratio compared to the QMC detector, the noise‐equivalent‐power for the nanohelix is about four orders of magnitude smaller than that for the QMC sensor. In addition, the time scale of current fluctuations which are needed to heat the nanohelix is at least one order of magnitude smaller than 20 μs response times of TES used in sub‐millimeter wave astronomy.

Very recently, superconducting W–C helical nanowires with nanohelix diameters down to 100 nm and nanowire diameters of about 50 nm have been successfully fabricated using He^+^ FIBID and revealed well‐defined resistance steps in their current–voltage (*I*–*V*) curves.^[^ ^30^ ^]^ The observed resistance steps are associated with different vortex and phase‐slip patterns according to the TDGL simulation results.^[^ ^30^ ^]^ The experimental realization of superconducting helical nanoarchitectures^[^ ^29^, ^30^ ^]^ sets the stage for the extension of vortex matter^[^ ^65^ ^]^ toward curvilinear chiral geometries, whose experimental and theoretical investigations represent an essentially novel research direction in Abrikosov fluxonics.^[^ ^66^ ^]^ In particular, individual nanohelices can be considered as building blocks for metamaterials whose microwave response should be tunable by the choice of working points in the *I*–*V* curves of individual nanohelices, by analogy with planar nanostructured superconductors.^[^ ^728^ ^]^ Furthermore, the intrinsic periodicity of the nanohelices allows one to anticipate commensurability effects in the presence of external magnetic fields,^[^ ^71^, ^729^ ^]^ which can be considered as yet another degree of freedom for a fine‐tuning of the system response.

#### 5.2.3 Toward Vortex Matter on Spherical Nanoshells

In mesoscopic superconductors, where the volume‐to‐surface area ratio is small,^[^ ^730^ ^]^ strong lateral confinement may cause the formation of multiquanta (“giant”) vortices,^[^ ^731^, ^732^ ^]^ while the shape of the boundary determines the symmetry of the final vortex configuration. In this regard, the definition of giant‐ and multivortex states becomes more complicated if the problem is extended into the third dimension, where vortices experience a complicated 3D interaction with the sample boundary, giving rise to rich vortex phase diagrams.^[^ ^685^, ^699^, ^700^ ^]^ In particular, in superconducting spherical nanoshells the surface curvature is expected to lead to a Magnus–Lorentz force, which pushes the vortices and antivortices toward the opposite poles of the shell. This can be considered as an effective pinning of vortices and antivortices at the poles, which strongly affects both the equilibrium distributions of vortices and their dynamics.^[^ ^10^, ^682^, ^685^ ^]^ For instance, superconducting spherical nanoshells have been predicted to be promising candidates for realizing giant vortex states, and for engineering phase transitions between those states and a vortex lattice.^[^ ^685^ ^]^ Such nanoshells are expected to allow the coexistence of Meissner and vortex states in equilibrium on one and the same superconducting film, that drives the phase transition to higher magnetic fields.^[^ ^10^ ^]^ In addition, the real part of the ac magnetic permeability of nanoshells with nonuniform thickness, arranged in a 3D array, is predicted to be tunable from large positive values at high dc fields to negative values at lower dc fields.^[^ ^686^ ^]^ Furthermore, a magnetic inclusion inside a superconductor sphere is expected to give rise to a complex evolution of confined vortex loops and external vortex pairs.^[^ ^683^ ^]^ However, the fabrication of such structures is at the edge of current fabrication capabilities. We anticipate that the fabrication of core–shell and, probably, thin‐walled superconducting shells by template‐assisted FIBID and FEBID will become possible in the years to come.

### 5.3 Summary and Outlook

In all, given the rapid development of nanofabrication technologies in conjunction with the plethora of exciting theoretical predictions awaiting their experimental examination, one can anticipate that curvilinear superconductors will be extensively investigated in the next years. At present, W–C^[^ ^30^, ^663^, ^733^ ^]^ and Nb–C^[^ ^714^, ^734^ ^]^ direct‐write superconductors are materials for which 3D nanofabrication has been demonstrated by FIBID. Remarkably, ultrafast vortex velocities have been recently demonstrated for Nb–C‐FIBID^[^ ^714^ ^]^ providing access to studying rich physics generic to non‐equilibrium collective systems. This allows for the Cherenkov‐like generation of sound^[^ ^735^, ^736^ ^]^ and spin^[^ ^737^, ^738^ ^]^ waves by moving fluxons and opens up novel routes to excite waves in magnon spintronics.^[^ ^739^, ^740^ ^]^ At the same time, the availability of novel precursor gases for the fabrication of 3D nanoarchitectrues by FEBID is anticipated.^[^ ^277^ ^]^ An important finding is that extending 2D superconductor spiral structures into the third dimension essentially improves the performance of helical microwave bolometers.^[^ ^29^ ^]^ This allows one to expect examination of curvilinear 3D superconducting structures in other domains of science and technology such as magnetoelectronics,^[^ ^40^, ^741^ ^]^ spintronics,^[^ ^742^, ^743^ ^]^ magnonics^[^ ^131^, ^744^, ^745^, ^746^ ^]^ and metamaterials with 3D superconductors and chirality.^[^ ^747^ ^]^ Furthermore, the combination of superconductors with ferromagnets in curvilinear geometries should open novel prospects for superconducting spintronics,^[^ ^748^ ^]^ proximity‐induced spin‐triplet superconductivity,^[^ ^651^, ^749^ ^]^ magnetic cloaking,^[^ ^667^, ^750^ ^]^ hybrid superconductor‐ferromagnetic metamaterials^[^ ^751^, ^752^, ^753^ ^]^ and the improvement of current‐carrying ability of superconductors.^[^ ^754^, ^755^ ^]^ In addition, ferromagnetic decoration of curvilinear superconductors is expected to modify the signatures of vortex guiding^[^ ^756^, ^757^ ^]^ and ratchet effects,^[^ ^758^ ^]^ flux‐flow instability,^[^ ^722^, ^756^, ^759^ ^]^ microwave‐stimulated superconductivity in the vortex state,^[^ ^716^, ^760^ ^]^ thereby advancing the development of 3D fluxonic circuits.^[^ ^29^, ^30^ ^]^

## 6 Conclusions

Exploration of curvilinear effects is a cross‐disciplinary research field, which covers different topics of soft matter, living, and condensed matter systems, including semiconductors, 2D van der Waals materials, plasmonics, optics, magnetism, and superconductivity. In this work we focused on curvilinear effects in magnetism and superconductivity aiming to show different perspective research directions both for fundamental and application communities. Much attention was dedicated to the development of theoretical and numerical methods in the description of curvilinear effects. In the fabrication and characterization parts, we summarized the cross‐scale methods and tools allowing to address curvilinear architectures from atomic to mesoscale. Many methods for fabrication (e.g., STM, DNA‐origami, 2D materials) and characterization (e.g., NV center microscopy) are mentioned acknowledging their strong relevance for the curvilinear magnetism community. Still, their use to produce and study responses of curved objects are yet to be validated. Great attention has been brought to various application proposals that arise from curvilinear geometries and are at different levels of readiness for technological implementation. Most of them require further deepening of theoretical framework and wait for experimental realization in the form of device prototypes. At the same time, there are already mature technologies, especially those related to shapeable magnetoelectronics, that reached the sufficiently high technological readiness level to anticipate rapid industrial implementation of flexible and printable sensor technologies. We expect that this review will stimulate further developments in curvilinear magnetism and superconductivity, their interconnections with other research communities addressing curvature‐induced effects in live science, soft, and condensed matter, as well as, industrial explorations by high‐tech spin‐offs and research and development‐oriented companies.

## Conflict of Interest

The authors declare no conflict of interest.
